# Optogenetics with Atomic Precision—A Comprehensive Review of Optical Control of Protein Function through Genetic Code Expansion

**DOI:** 10.1021/acs.chemrev.4c00224

**Published:** 2025-02-10

**Authors:** Maura Charette, Carolyn Rosenblum, Olivia Shade, Alexander Deiters

**Affiliations:** Department of Chemistry, University of Pittsburgh, Pittsburgh, Pennsylvania 15260, United States

## Abstract

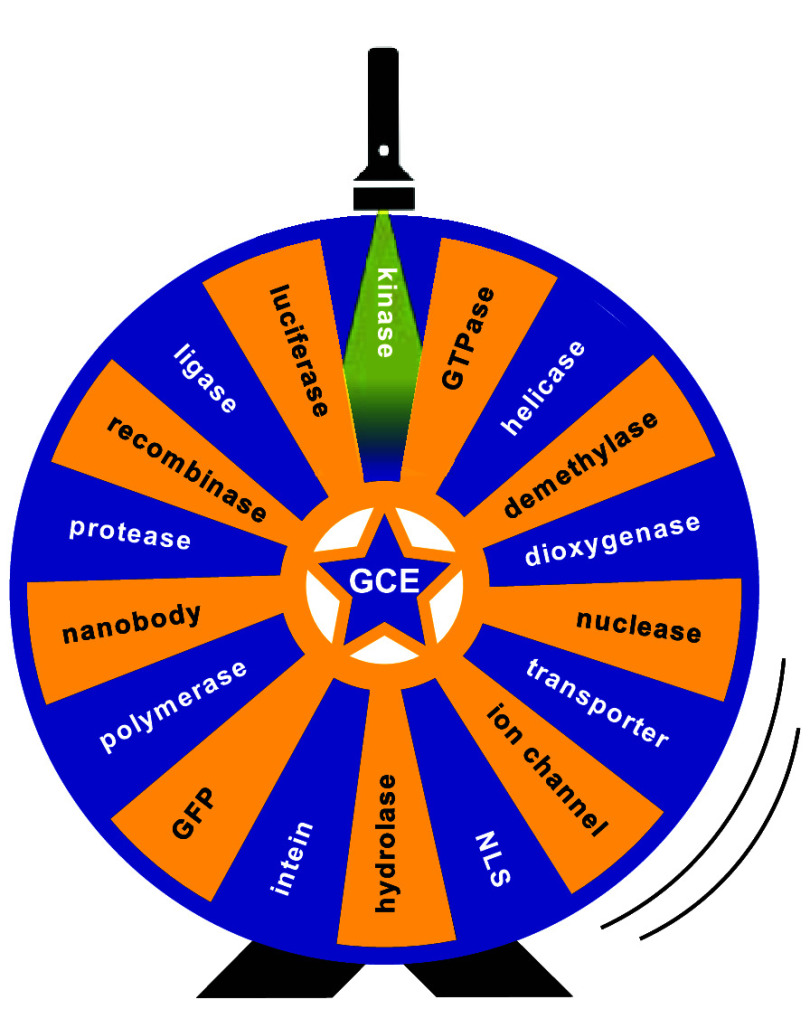

Conditional control of protein activity is important in order to elucidate the particular functions and interactions of proteins, their regulators, and their substrates, as well as their impact on the behavior of a cell or organism. Optical control provides a perhaps optimal means of introducing spatiotemporal control over protein function as it allows for tunable, rapid, and noninvasive activation of protein activity in its native environment. One method of introducing optical control over protein activity is through the introduction of photocaged and photoswitchable noncanonical amino acids (ncAAs) through genetic code expansion in cells and animals. Genetic incorporation of photoactive ncAAs at key residues in a protein provides a tool for optical activation, or sometimes deactivation, of protein activity. Importantly, the incorporation site can typically be rationally selected based on structural, mechanistic, or computational information. In this review, we comprehensively summarize the applications of photocaged lysine, tyrosine, cysteine, serine, histidine, glutamate, and aspartate derivatives, as well as photoswitchable phenylalanine analogues. The extensive and diverse list of proteins that have been placed under optical control demonstrates the broad applicability of this methodology.

## 1 Introduction

Conditional control over protein activity is a critical goal in the study of the functions and interactions of proteins. Optical control has several advantages over other control modalities, as it enables both spatial and temporal control over protein activity, is noninvasive in cells and animals, and is easily tunable both in wavelength and amplitude.^1−3^ Photoregulation is also minimally toxic, allowing for use in living organisms.^3^ A variety of approaches have been used to introduce optical control over protein activity.

Traditional optogenetics utilizes naturally occurring photosensitive domains genetically fused to proteins of interest.^1,2^ These domains can be linked to protein activity in a variety of ways. Upon irradiation, these domains can undergo a conformational change that affects protein activity, can dimerize to bring two halves of a split protein together, or can release a protein from steric inhibition.^1,2^ Commonly utilized photosensitive domains include light-oxygen-voltage (LOV) domains, cryptochromes, phytochromes, and opsin ion channels.^1,2,4^ These systems have been used in a wide variety of applications, including introducing photocontrol over ion channels, recombinases, GTPases, and several other proteins.^1,5−9^

Photopharmacology takes an alternate approach to linking protein activity to light exposure.^2,3,10,11^ It utilizes photoactive protein antagonists or agonists that feature either photolabile or photoswitchable motifs to optically regulate binding. Light irradiation decages or reversibly isomerizes the ligand, thereby increasing or decreasing binding affinity. Photoswitchable ligands can also be tethered to the protein itself.^2,12−15^ Photopharmocological methods have been applied to a wide range of small molecule ligands, including cofactors, neurotransmitters, kinase and microtubule inhibitors, as well as proteolysis targeting chimeras (PROTACs).^16−19^

A third method for optical control of protein activity is the incorporation of photoactive noncanonical amino acids (ncAAs) into proteins via genetic code expansion in cells and animals.^20−25^ These ncAAs include photocaged amino acids, which feature photolabile protecting groups–so-called caging groups–and photoswitchable ncAAs, which undergo configurational changes upon irradiation.^2,21,26,27^ Genetic code expansion allows for the incorporation of small (<300 Da), minimally disruptive caging groups^28^ with genetically determined specificity, allowing for temporary perturbation of protein activity without major changes to the overall protein structure, as is the case of fusions with photoactive domains. Identification of sites for caging can generally be placed on a rational foundation based on structural or mechanistic information, such as key residues required for catalytic activity, substrate binding, protein–protein interactions, etc.^2,5,7,29^ Decaging through light exposure generates the wild-type protein, allowing for the study of protein activity with spatial and temporal precision, without the need for fusion constructs.^2,30,31^ While caging groups have also been introduced into proteins and peptides synthetically, genetic code expansion eliminates the need for delivery of the caged protein into cells or animals and dramatically reduces synthetic requirements.^30,32−34^

A wide range of photoactive ncAAs have been genetically incorporated into proteins in a number of systems, including bacterial cells, mammalian cells, yeast, zebrafish, nematodes, frogs, and mice.^20,35−37^ A number of amino acids, including lysine, tyrosine, cysteine, serine, histidine, glutamate, and aspartate have been caged with photolabile groups, while phenylalanine derivates containing photoswitchable motifs have also been generated. This review aims to comprehensively summarize the development and application of genetic code expansion with photocontrolled amino acids.

## 2 Genetic Code Expansion

**Figure 1:**
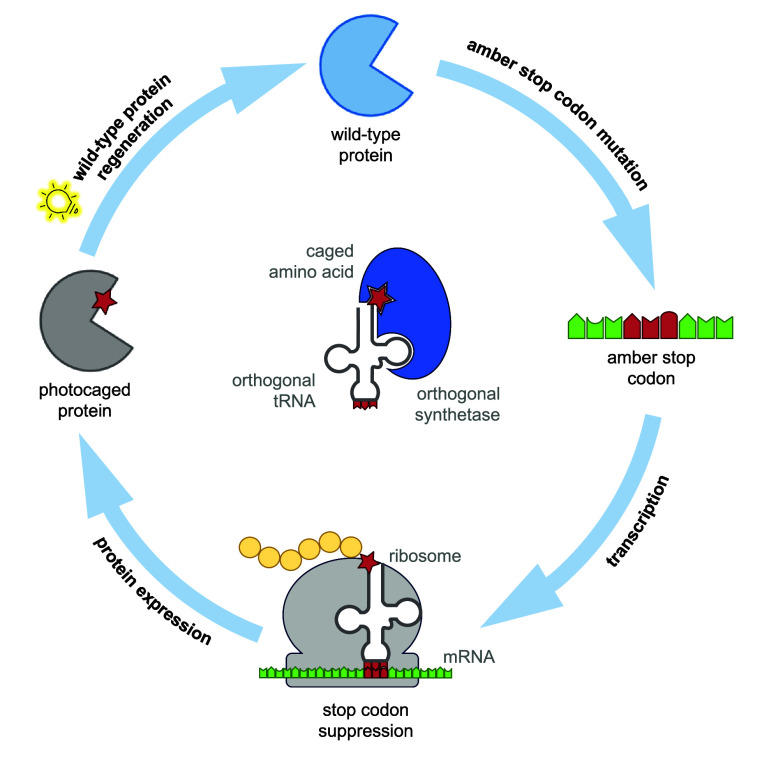
Overview of genetic code expansion. To introduce optical control over a protein via genetic code expansion with a photocaged ncAA, the codon corresponding to a key residue in a protein is mutated to an amber stop codon (UAG). An orthogonal tRNA is acylated with the caged amino acid by an optimized orthogonal synthetase, which is then used to incorporate the ncAA at the amber stop codon during translation. An inactive form of the protein is then expressed with the photocaged ncAA at the desired site, and subsequent irradiation decages the ncAA and leads to regeneration of the wild-type protein.

A brief overview of genetic code expansion is provided below. For a more in-depth discussion of the different parts and their engineering, please see the other reviews in this special issue of *Chem. Rev.* At its core, genetic code expansion is the reprograming of a stop codon, most commonly the UAG amber stop codon, for site-specific incorporation of an ncAA by the ribosome (Figure 1).^22,25,38−41^ The amber stop codon is the least frequently occurring stop codon in many organisms, including bacterial and mammalian cells.^20^ For example, only around 9% of stop codons in *E. coli* are amber codons, allowing for reprogramming without affecting organism viability.^20,22,36^

Two components need to be added to a cell for successful genetic code expansion: 1) a unique tRNA that recognizes the amber codon, and 2) a cognate tRNA synthetase (RS) that acylates this tRNA with the desired ncAA (see Table 1).^20^ These two parts need to be orthogonal to the endogenous protein biosynthetic machinery in the selected organism. Specifically, the tRNA should not be a substrate for an endogenous synthetase and the synthetase should not recognize an endogenous amino acid nor acylate an endogenous tRNA. There are a variety of synthetase/tRNA pairs that have been optimized for this purpose, including the TyrRS/TyrT pair from the archaea *M. jannaschii* (*Mj*TyrRS), LeuRS and TyrRS from *E. coli (Ec*LeuRS and *Ec*TyrRS), TrpRS from yeast, and the PylRS/PylT pair from a variety of archaea including *M. mazei* (*Mm*PylRS), *M. barkeri* (*Mb*PylRS), and *M. alvus* (*Ma*PylRS).^20,36,42^

**Table 1:** Synthetases Evolved to Encode Light-Responsive ncAAs Described in This Review

parent AA	ncAA	species	parent RS	synthetase name	synthetase mutations	ref
**K**	**ONBK**	*Mm*	PylRS	ONBKRS	Y306M, L309A, C348A, Y384F	(67)
	**PCK**	*Mb*	PylRS	PCKRS	M241F, A267S, Y271C, L274M	(68)
	**HCK**	*Mb*	PylRS	HCKRS	Y271A, L274M	(69)
	**ACK**					(70)
	**BrCK**					(69)
	**MeONBK**	*Mb*	PylRS	MeONBKRS	Y271I, L274M, C313A	(71)
		*Mm*	PylRS	MeONBKRS2	Y306M, L309A, C348T, T364 K	(72)
**Y**	**ONBY**	*Mj*	TyrRS	ONBYRS	Y32G, L65G, F108E, D158S, L162E	(73)
		*Mb*	PylRS	ONBYRS2	L270F, L274M, N311G, C313G	(74)
		*Mb*	PylRS	ONBYRS3	L270F, L274M, N311G, C313G, Y349F	(75)
	**NPY**	*Mb*	PylRS	ONBYRS3	L270F, L274M, N311G, C313G, Y349F	(75)
	**MNPY**	*Mb*	PylRS	ONBYRS3	L270F, L274M, N311G, C313G, Y349F	(75)
	**NPPY**	*Mb*	PylRS	ONBYRS3	L270F, L274M, N311G, C313G, Y349F	(75)
	**FnbY**	*Mb*	PylRS	FnbYRS	270F, L274M, N311A, C313G	(76)
	**FmnbY**	*Ma*	OylRS	FmnbYRS	L125F, N166A, V168G	(77)
	**ONB2FY**	*Mj*	TyrRS	ONB2FYRS	Y32G, L65G, H70N, F108G, D158S, I159M	(78)
	**ONB3FY**	*Mj*	TyrRS	ONB2FYRS	Y32G, L65G, H70N, F108G, D158S, I159M	(78)
	**ONBDFY**	*Mj*	TyrRS	ONBDFYRS	Y32G, L65G, H70M, F108G, D158S, I159M, L1D62E	(78)
	*m***-ONB-DOPA**	*Mj*	TyrRS	ONB-DOPARS-1	Y32A, L65I, H70, Q109A, D158A, I159A, L162M, A167N, A180Q	(79)
**C**	**ONBC**	*Ec*	LeuRS	ONBCRS	M40W, L41S, Y499I, Y527A, H537G	(53)
		*Mb*	PylRS	PCC1RS	N311M, C313Q, V366G, W382N, R85H	(80)
	**DMNBC (Cmn)DMMNBC**	*Ec*	LeuRS	DMNBSRS	M40G, L41Q, Y499L, Y527G, H537F, T252A	(44, 81, 82)
	**NPC**	*Mb*	PylRS	PCC2RS	N311Q, C313A, V366M	(80, 83)
		*Mm*	PylRS	PCCRS	N311Q, C313A, V366M	(80, 84)
	**NP-CA-C**	*Mb*	PylRS	PCKRS	M241F, A267S, Y271C, L274M	(68, 85)
	**NP-CA-HC**	*Mb*	PylRS	PCKRS	M241F, A267S, Y271C, L274M	(68, 85)
**U**	**NPU**	*Mb*	PylRS	PCC2RS	N311Q, C313A, V366M	(80, 86)
	**DMNBU**	*Ec*	LeuRS	DMNBSRS	M40G, L41Q, Y499L, Y527G, H537F, T252A	(44, 87, 88)
**S**	**DMNBS**	*Ec*	LeuRS	DMNBSRS	M40G, L41Q, Y499L, Y527G, H537F, T252A	(44)
**H**	**ONBH**	*Mb*	PylRS	ONBHRS	N311G, C313 V, V366 K	(89)
**F**	**AzoF**	*Mj*	TyrRS	AzoFRS	Y32G, L65E, F108A, Q109E, D158G, L162H	(90)
	**F2AzoF**	*Mb*	PylRS	AzoFRS2	L270F, L274M, N311G, C313G, Y349F	(91)
	**F3AzoF**	*Mm*	PylRS	AzoFRS-4	Y31G, L65 V, D158G, I159Y, L162R	(92)
	**F4AzoF**	*Mb*	PylRS	AzoFRS2	L270F, L274M, N311G, C313G, Y349F	(91)
	**DBTDA**	*Mm*	PylRS	*cis*-DBTDARS	A302T, N346A, C348G, Y384F, W417T	(93)
	**DBDAA**	*Mm*	PylRS	*trans*-DBDAARS	A203T, N346A, C348G, Y384F, V401L, W417T	(94)
	**PSCaa**	*Mm*	PylRS	PSCaaRS	302T, 309S, 322I, 346 V, 348G, 384Y, 401 V, 417W, 419G	(95)
	**Cl-PSCaa**	*Mm*	PylRS	PSCaaRS	302T, 309S, 322I, 346 V, 348G, 384Y, 401 V, 417W, 419G	(95)
	**Keto-PSCaa**	*Mm*	PylRS	KetoPSCaaRS	302T, 309A, 322T, 346A, 348G, 401 V, 417W, 419G	(95)
	**F-PSCaa**	*Mm*	PSCaaRS	PSCaaRS	302T, 309S, 322I, 346 V, 348G, 384Y, 401 V, 417W, 419G	(96)
	**AAPF**	*Mb*	PylRS	AzoFRS2	L270F, L274M, N311G, C313G, Y349F	(97)
		*Mj*	TyrRS	RS	Y31G, L65 V, D158C, I159D, L162G, A167L	(98)
**E**	**ONBE**	*Mb*	PylRS	ONBERS	N311S, C313G, Y349F	(99)
	**NPE**	*Mb*	PylRS	NPERS	A267D, N311G, C313G	(99)
	**MNIE**	*Ec*	LeuRS	MNIERS	Y499C, Y527G, H537T	(100)
**D**	**ONBD**	*Mb*	PylRS	ONBDRS	L270 V, L274G, Q311C, C313W, Y349F	(101)
	**MeONBD**	*Mb*	PylRS	ONBDRS	L270 V, L274G, Q311C, C313W, Y349F	(101)
**DAP**	**PC-DAP**	*Mb*	PylRS	DAPRS	Y271C, N311Q, Y349F, V366C	(102)
**CIT**	**PC–CIT**	*Ec*	LeuRS	CITRS	M40I, Y499I, Y527A, H529G, T252A	(103)

The greatest limitation of the *Mj*TyrRS system is its lack of orthogonality in eukaryotic cells. A number of *E. coli* synthetases have been employed for genetic code expansion in yeast and mammalian systems, including *Ec*TyrRS and *Ec*LeuRS.^43,44^ While *E. coli* LeuT is directly orthogonal in yeast, *B. stearothermophilus* TyrT is used in place of the *E. coli* TyrT for genetic code expansion in mammalian cells due to the inclusion of a necessary internal promoter sequence.^43^ The most common synthetase/tRNA pair used in modern genetic code expansion is the PylRS/PylT pair.^45^ PylT is a natural amber suppressor and PylRS is orthogonal in both pro- and eukaryotic cells. PylRS also has a large hydrophobic active site evolved to recognize pyrrolysine^20^ and does not feature an editing domain, unlike other synthetases.^45^ The deep binding pocket of PylRS is distant from its catalytic core, allowing mutations of residues around the pocket for recognition of bulkier ncAAs without risking interruption of catalytic activity.^45^

The most commonly used PylRS synthetases are those from *M. mazei* and *M. barkeri*.^20,36,45^*Mm*PylRS and *Mb*PylRS share a highly conserved catalytic core, but have N-terminal regions of varying lengths.^20^ A chimeric synthetase consisting of the more soluble N-terminal domain of *Mb*PylRS and the more catalytically active C-terminal domain of *Mm*PylRS has also been used for ncAA incorporation, and has shown even higher rates of incorporation than either individual synthetase.^46^ The PylRS from *M. alvus* has also been recently used for ncAA incorporation.^47^*Ma*PylRS overcomes some of the limitations of *Mb*PylRS and *Mm*PylRS as it has a simpler structure than the classic PylRS isoforms, providing increased solubility and stability.^47^*Ma*PylRS lacks the N-terminal domain of the classic *Mb* or *Mm*PylRS synthetases, which recognizes the T-arm and variable loop of PylT. Otherwise, the structure of *Ma*PylRS is similar to other PylRS, but only shares around 35% sequence similarity.^47^

For incorporation of a given ncAA, a synthetase must be found that is able to acylate the desired tRNA with the ncAA of interest. The most commonly used method of synthetase optimization involves alternating rounds of positive and negative genetic selection starting with a library of synthetase mutants, where incorporation of a ncAA either results in cell survival or cell death, respectively.^48^ Chloramphenicol acetyl transferase is often used for positive selections, as introduction of an amber stop codon into the enzyme only results in bacterial cell survival in the presence of chloramphenicol if a ncAA is encoded. Failure to incorporate the ncAA results in cell death in the presence of the antibiotic.^48^ This positive selection method does not eliminate synthetase mutants that may incorporate endogenous amino acids instead of the desired ncAA, however. To account for this issue, a subsequent round of negative selection is conducted using a barnase lethality gene or uracil phosphoribosyltransferase (UPRT) gene with a premature amber stop codon.^48,49^ Cell survival in the absence of the ncAA then validates the lack of endogenous amino acid incorporation. Repeating this series of positive and negative selections with a library of mutant synthetases results in the identification of a mutant that is specific for incorporation of the desired ncAA with minimal background incorporation of endogenous amino acids.^50^

Other selection markers have also been used in synthetase selections, such as *LacZ*, *His3*, *Ura3*, or fluorescent markers.^51−53^ Fluorescent markers allow for rapid characterization of hits through methods such as fluorescence automated cell sorting (FACS).^51^ Most phenotypic-based methods of selection, however, suffer from some level of inconsistency.^54^ Relative protein expression levels or solubility of different synthetase mutants may prevent identification of true hit synthetases. To overcome this limitation, multiplex automated genome engineering (MAGE) has been employed for synthetase optimization through the generation of chromosomally integrated libraries of *Mj*TyrRS mutants.^55^ In addition to the aminoacyl tRNA synthetase, a selection marker, specifically a UAG-containing *tolC,* is also introduced into the genome, making cells sensitive to the presence of colicin E1. Consistent amounts of both proteins are expressed, giving a more accurate readout of amino acid incorporation.^54,55^ After a series of rounds of MAGE using a pool of single stranded DNA oligonucleotides, a round of negative selection exposing cells expressing *tolC*-UAG to colicin E1 without exposure to the ncAA of interest, followed by a FACS-based round of positive selection with GFP-UAG, leads to the identification of optimal synthetases.^55^

Other methods of synthetase engineering include phage-assisted continuous or noncontinuous evolution (PACE or PANCE), tRNA extension (tREX) and its derivatives such as tRNA display, and a method that selects for tRNA-acylation without ribosomal translation (START); however, these methods have not yet been used to generate a synthetase for incorporation of photoactive ncAAs.^46^ Next-generation sequencing has enabled tracking of the success of each synthetase in libraries used in any of these methods, revealing the relative abundance of each mutant from a pool of those that passed the selection process.^56−60^ Some residues that are often mutated in the generation of synthetase libraries include those that form the amino acid binding pocket in *Mm*PylRS, such as A302, L305, Y306, L309, N346, C348, and W417.^45^ Y384 is also often mutated, as it is part of the flexible region that restructures from a random loop to form a cap on top of the binding pocket upon amino acid interaction. Studies of crystal structures of synthetases bound to their ncAAs have helped to elucidate these key residues and their interactions with ncAA substrates to facilitate the design of subsequent synthetase libraries.^61^ Synthetase libraries might also be computationally designed based on the likelihood of certain mutations for introducing ncAA specificity.^62^

Methods of tRNA optimization have also been developed, such as the virus-assisted directed evolution strategies (VADER and VADER 2.0) for genetic code expansion in mammalian systems, which use tRNA libraries paired with adeno-associated virus (AAV) particles with a TAG codon in their capsid proteins to isolate active tRNA through the ability of the virus to replicate.^57,58^ Using an azide-containing ncAA to test for incorporation, subsequent dibenzocyclooctyne (DBCO)-biotin treatment and streptavidin pulldown allows for labeling and isolation of AAV particles with tRNA that incorporated the ncAA and separation of mutants that only incorporated natural amino acids into capsid proteins. As current methods of genetic code expansion in mammalian cells require hundreds of tRNA-expressing genes per cell, optimization methods such as these that decrease the necessary copies of tRNA will further streamline synthetase identification.^63,64^

With the evolution of many tRNA/RS pairs, genetic code expansion has been used to encode photoactive ncAAs in a variety of organisms, including *E. coli*, mammalian cells, *C. elegans*, zebrafish, frogs, and mice.^20,35,36,65^ A variety of *E. coli* strains have been designed to increase mutagenesis efficiency, such as those with all native amber stop codons replaced with alternate stop codons, or those with release factor 1 (RF1) deleted, preventing competition between RF1 and the suppressor tRNA for the amber stop codon.^23,25^

While GCE is a versatile tool that has allowed for the generation of a plethora of ncAA-containing proteins, it also faces limitations. Obtaining synthetases that are specific for a given ncAA can be a difficult task, and some ncAAs are unable to be incorporated via GCE, e.g., due to their toxicity.^24,66^ While many ncAAs have become commercially available, that is not universally the case.^23^ Lastly, while GCE is most established in cell culture, it has only been demonstrated in select animals to date.^20,65^

## 3 Lysine

Caged lysine derivates are frequently used to achieve optical control over enzyme activity. Installation of a light-removable protecting group eliminates its positive charge, preventing electrostatic interactions such as those with negatively charged nucleic acids, and inhibiting post-translational modifications such as acetylation, acylation, methylation, and ubiquitination.^68,120,165^ Photolabile caging groups also add steric bulk to the side-chain, offering the ability to block a substrate from entering an active site or interacting with other key residues.^70,115,166^ The high frequency of essential lysines in enzymes has allowed photocaged lysines to be utilized in a variety of applications, including methods to control gene expression, cell signaling, protein degradation, and protein activity through the caging of a diverse set of proteins ranging from recombinases to kinases, helicases, polymerases, and many more (see Table 2).

**Table 2:** Summary of Proteins Successfully Photocontrolled with Photocaged ncAAs

class	protein	ncAA	optically controlled amino acid function	section	ref
ABC transporter	TAP	**PCK**	electrostatic interaction	3	(104)
biotin ligase	TurboID	**PCK**	H-bonding	3	(105)
luciferase	Fluc	**HCK**	H-bonding	3	(106)
		**ONBY**	sterics	4	(75, 107)
		**NPY**	sterics	4	(75, 106)
		**MNPY**	sterics	4	(75)
		**NPPY**	sterics	4	(75)
		**ONBH**	sterics	7	(89)
		**ONBD**	H-bonding	8	(101)
	Nluc	**ONBY**	sterics	4	(107)
	Rluc	**NP-CA-HYC**	sterics	5	(85)
		**NP-CA-C**	sterics	5	(85)
		**ONBH**	basicity	7	(89)
		**NPOMH**	basicity	7	(108)
dehydrogenase	IDH	**PCK**	sterics	3	(109)
demethylase	FTO	**ONBY**	sterics	4	(107)
dioxygenase	TET1	**DMNBS**	sterics	6	(110)
	TET2	**DMNBS**	sterics	6	(111)
fluorescent protein	BFP	**ONBH**	sterics	7	(89)
	GFP	**ONBY**	sterics	4	(113, 114)
		**ONB2FY**	sterics	4	(78)
		**ONB3FY**	sterics	4	(78)
		**ONBDFY**	sterics	4	(78)
		*m***-ONB-DOPA**	sterics	4	(79)
		**ONBE**	electrostatic interaction	8	(99)
		**NPE**	electrostatic interaction	8	(99)
GTPase	KRAS	**ONBY**	sterics	4	(107)
		**ONBD**	metal coordination	8	(101)
		**MeONBD**	metal coordination	8	(101)
	NRAS	**ACK**	H-bonding	3	(70)
		**HCK**	H-bonding	3	(106)
helicase	UvrD	**HCK**	electrostatic interaction	3	(115)
hydrolase	β-galactosidase	**ONBY**	acidity	4	(73)
intein	Gp41-1	**ONBY**	sterics	4	(116)
	M86	**ONBY**	sterics	4	(117)
	mCherry/Src	**DMMNBC**	nucleophilicity	5	(118)
ion channel	CFTR	**DMNBS**	phosphorylation	6	(119)
	Kir 2.1	**DMNBC**	sterics	5	(81)
kinase	Adk	**HCK**	H-bonding	3	(120)
	BRAF	**ONBD**	metal coordination	8	(101)
		**MeONBD**	metal coordination	8	(101)
	LCK	**PCK**	electrostatic interaction	3	(121)
	LepB	**HCK**	sterics	3	(122)
	MEK1	**PCK**	electrostatic interaction	3	(123)
		**HCK**	electrostatic interaction	3	(124)
		**ONBY**	sterics	4	(107)
		**ONBD**	phosphate mimic	8	(101)
		**MeONBD**	phosphate mimic	8	(101)
	MKK6	**PCK**	electrostatic interaction	3	(125)
		**HCK**	electrostatic interaction	3	(126)
	p38	**HCK**	electrostatic interaction	3	(127)
	PKA	**ACK**	H-bonding	3	(70)
nanobody	7D12 (anti-EGFR)	**ONBY**	sterics	4	(128, 129)
	2Rs15d (anti-HER2)	**ONBY**	sterics	4	(130)
	anti-ALFA-tag	**ONBY**	H-bonding	4	(131)
	anti-sfGFP	**ONBY**	sterics	4	(132, 133)
		**NPY**	sterics	4	(132, 133)
	EgA1 (anti-EGFR)	**ONBY**	sterics	4	(130)
NLS	OptoNLS	**PCK**	basicity	3	(134, 135)
	p53 bipartite NLS	**PCK**	basicity	3	(68)
	TDP-43 NLS	**ONBK**	H-bonding	3	(136)
nuclease	zinc finger nuclease	**ONBY**	sterics	4	(137)
	Cas9	**PCK**	sterics	3	(112)
peptide	PDP-*NaI*	**ONBY**	sterics	4	(138)
phosphatase	MKP3	**HCK**	electrostatic interaction	3	(139)
		**DMNBC**	nucleophilicity	5	(139)
	SHP2	**HCK**	electrostatic interaction	3	(140)
		**NPY**	H-bonding	4	(140)
phosphotransferase	SopB	**HCK**	sterics	3	(141)
PIP-binding domains	PLCδ1 PH domain	**HCK**	H-bonding	3	(142)
polymerase	T7 RNA polymerase	**PCK**	H-bonding, electrostatics	3	(143)
		**ONBY**	metal coordination	4	(144)
	Taq DNA polymerase	**ONBY**	sterics	4	(145)
protease	caspase 3	**ONBY**	sterics	4	(107)
		**ONBC**	nucleophilicity	5	(53)
		**NPC**	nucleophilicity	5	(84)
	HCVp	**NPOMH**	H-bonding, basicity	7	(108)
	HRV3C	**MNIE**	H-bonding, acidity	8	(100)
	SEAP	**MNIE**	metal coordination	8	(100)
	TEVp	**NPY**	H-bonding	4	(75)
		**NPC**	nucleophilicity	5	(75)
receptor	IL-24 receptor	**ONBY**	sterics	4	(146)
	TLR8	**ONBY**	sterics	4	(147)
recombinase	Cre recombinase	**PCK**	H-bonding	3	(148)
		**HCK**	H-bonding	3	(149)
		**ONBY**	nucleophilicity	4	(148, 150)
		**NPOMH**	sterics	7	(108)
self-labeling enzyme	SpyCatcher	**HCK**	nucleophilicity	3	(151)
		**ONBE**	acidity	8	(99)
		**NPE**	acidity	8	(99)
synthase	FAS-TE	**ONBY**	sterics	4	(152)
	GGGPS	**ONBY**	sterics	4	(153)
	ImGPS	**ONBY**	allostery	4	(154)
		**NPY**	allostery	4	(154)
synthetase	tryptophan synthetase	**ONBY**	sterics	4	(155)
toxin	lethal factor	**ONBY**	sterics	4	(107)
	YoeB_Sa1_	**ONBY**	sterics	4	(156)
transcription factor	Pho4	**DMNBS**	phosphorylation	6	(44)
	STAT1	**ONBY**	phosphorylation	4	(74)
transferase	chloramphenicol acetyltransferase	**ONBH**	H-bonding, basicity	7	(89)
	DNMT	**DMNBC**	nucleophilicity	5	(157)
	Ecm1	**ONBY**	H-bonding	4	(158)
	O-GlcNAc transferase	**PCK**	electrostatic interaction	3	(159)
	PRMT1	**ONBY**	sterics	4	(160)
ubiquitylation	PCNA	**NPC**	nucleophilicity	5	(83)
	ubiquitin	**FnbY**	photo-cross-linking	4	(77)
miscellaneous	AAV capsid	**PCK**	electrostatic interaction	3	(161)
	degron	**HCK**	electrostatic interaction	3	(162)
	fp-5	*m***-ONB-DOPA**	sterics	4	(79)
	FtsZ	**ONBY**	sterics	4	(163)
	SH2 domain	**ONBK**	electrostatic interaction	3	(164)
	Z protein	**FnbY**	photo-cross-linking	4	(77)
		**FmnbY**	photo-cross-linking	4	(77)

**Figure 2:**
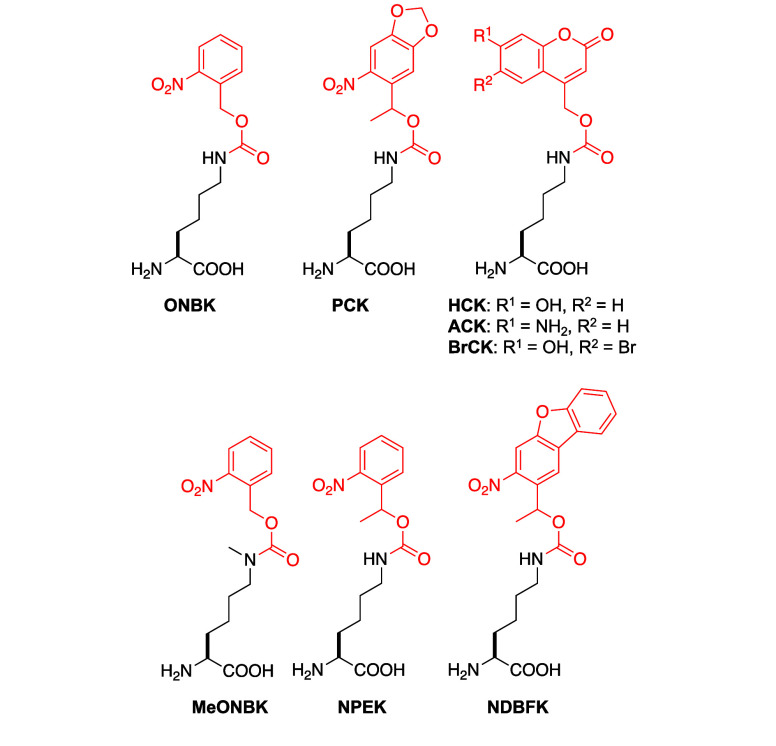
Structures of photocaged lysines incorporated into proteins through genetic code expansion with caging groups indicated in red.

One of the earliest genetically encoded photocaged lysines was the *o*-nitrobenzyl-caged lysine **ONBK** (Figure 2).^67^ An optimized *Mm*PylRS for incorporation of **ONBK** was isolated through a series of chloramphenicol resistance-based positive selections and barnase-based negative selections with a library of PylRS mutants containing five randomized residues around the pyrrolysine binding site. A synthetase with four mutations was identified (ONBKRS, see Table 1) and used to successfully incorporate **ONBK** into sfGFP and EGFP in *E. coli* and mammalian cells, respectively, as confirmed by mass spectrometry analysis.^67^

Addition of an electron donating group onto the benzene of the *o*-nitrobenzyl caging group red-shifts its absorbance.^26,68,143^ Therefore, the photocaged lysine genetically encoded for use in the greatest number of applications is nitropiperonylmethyloxycarbonyl lysine, **PCK** (Figure 2). This ncAA has been successfully incorporated in a range of organisms, including *E. coli*, yeast, mammalian cells, and *C. elegans*.^68,134,167,168^ In addition to its red-shifted absorbance, **PCK** also decages to yield a ketone byproduct, in comparison to the more reactive aldehyde released by **ONBK.**(68,143) The α-methyl group of **PCK** also enhances its decaging kinetics.^169^ As in the case of **ONBK**, a synthetase containing four mutations (PCKRS, Table 1) around the ncAA binding pocket was obtained through a double-sieve selection process and was confirmed by electrospray ionization mass spectrometry (ESI-MS) and MS/MS to incorporate **PCK** into myoglobin in *E. coli*.^68^

A number of coumarin-caged lysines have also been developed.^69,70^ Coumarin acts not only as a highly light-sensitive caging group, but also allows the ncAA to serve as a fluorescent probe.^69^ Hydroxycoumarin (**HCK**) and bromo-hydroxycoumarin lysine, (**BrCK,**Figure 2) were first designed to decage with blue and near-IR light, respectively.^69^**HCK** undergoes single photon decaging while **BrCK** undergoes two-photon decaging, allowing greater spatial control over decaging and increased tissue penetration from the longer wavelength light. Both ncAAs were incorporated with an *Mb*PylRS bearing the mutations Y271A and L274 M (HCKRS, Table 1).^69^ Y271A is a mutation known to facilitate incorporation of *N*^ε^-carbamate-linked lysines, while L274 M removes steric bulk at the back of the hydrophobic pocket, allowing space for greater flexibility of the ncAA side chain.^69^ These mutations also position the caging group to undergo a π-stacking interaction with W382, as well as an H-bonding interaction between the hydroxyl group of the coumarin and D373. Both ncAAs also maintain key interactions with residues known to play roles in ncAA recognition, such as H-bonding between the carbamate carbonyl and N311, and the carboxylic acid and R295.^69^ Both **HCK** and **BrCK** have been incorporated in *E. coli* and mammalian cells, and **HCK** has additionally been incorporated in zebrafish and *Xenopus*.^69,149^

In 2023, aminocoumarin lysine **ACK** (Figure 2) was developed as an improvement upon the **HCK** scaffold and was predicted to decage 3.4-times more efficiently than **HCK**.^70^ The amino analogue **ACK** features a 2.1-fold higher extinction coefficient, a red-shifted λ_max_ from 330 to 348 nm, a quantum yield almost twice that of **HCK** (0.04 vs 0.025, respectively), and a wider pH range in which it can successfully decage (pH 5–8). Further, **ACK** is recognized by the same synthetase as **HCK** and **BrCK**, and has been successfully incorporated into proteins in *E. coli*, mammalian cells, and zebrafish.^70^ Recent modifications to **ONBK** have also been made, including the addition of α-substitutions and expansion of π-electron systems to improve decaging efficiency.^170^ Specifically, 1-(2-nitrophenyl)-ethyl lysine (**NPEK,**Figure 2) was encoded into proteins in mammalian cells using HCKRS (Table 1). Expansion on the π-electron system of **ONBK** yielded nitrodibenzylfuranyl lysine (**NDBFK**, Figure 2), which was also encoded using HCKRS and has fast decaging kinetics.^170^

**Figure 3:**
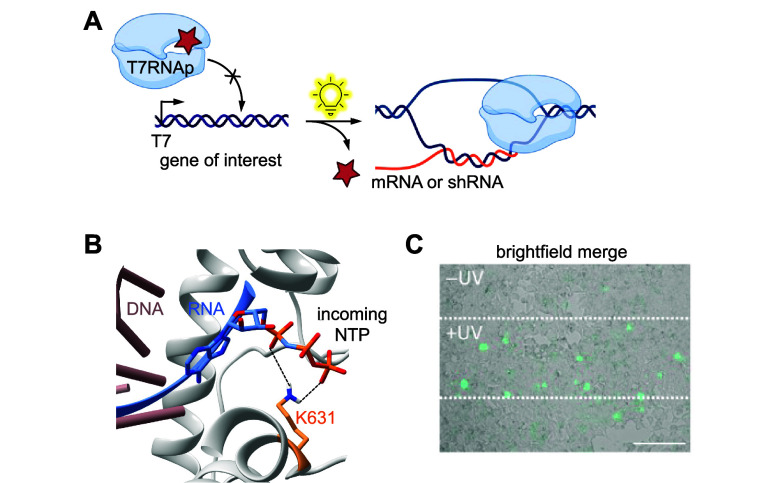
Photocaged RNA polymerase. (A) Photocaged T7RNAp is inactivated until irradiation with 365 nm light, upon which activity is restored. (B) Crystal structure of T7RNAp-K631 showing the key interaction of K631 with the incoming NTP at the α-phosphate (PDB 1S76).^143^ (C) Representative micrograph of T7RNA-K631**PCK** activation of T7-IRES-EGFP expression via irradiation through a horizontal slit. Reproduced with permission from ref (143). Copyright 2013 American Chemical Society.

Photocaged lysines have been used to introduce photocontrol over a wide variety of functions ranging from gene expression to post-translational modifications and cell signaling. Some of the early work utilizing photocaged lysines involved controlling gene expression with light.^112,134,143,148,149,167,171,172^ One approach incorporated **PCK** into T7 RNA polymerase (T7RNAp) to optically activate transcription of DNA into mRNA and short hairpin RNA (shRNA) in mammalian cells.^143^ By introducing a caged bacteriophage polymerase into mammalian cells, an orthogonal system of gene expression is introduced that will not be activated by endogenous mammalian machinery.^143^ Expression of introduced genes of interest under a T7 promoter would then solely occur after irradiation activates the T7RNAp and transcription is initiated (Figure 3A). K631 is a crucial residue that stabilizes an incoming NTP by forming an H-bond with its α-phosphate (Figure 3B).^143^ Introducing a bulky caging group at K631 sterically occludes NTPs from the active site while also eliminating the positive charge on lysine, preventing interaction with the NTP. Successful introduction of optical control was demonstrated through use of firefly luciferase and EGFP reporters under the control of T7 promoters in HEK293T cells (Figure 3C).^143^ Optical control of RNA interference was also demonstrated using T7RNAp-K631**PCK** to regulate transcription of a short hairpin RNA (shRNA). Specifically, Eg5, a gene that encodes a motor protein involved in mitosis, was silenced through light-triggered shRNA expression as exhibited through the formation of binucleated cells, a known phenotype of Eg5 inhibition.^143,173,174^ RNA inhibition was further shown through qRT-PCR analysis of cellular RNA extracts before and after irradiation. A 55% reduction in Eg5 mRNA was observed after irradiation, matching the gene silencing caused by wild-type T7RNAp.^143^ This technology can be paired with any gene or shRNA of interest under a T7 promoter to control gene expression or inhibition via light, making it a widely applicable tool. T7RNAp was also caged with a photocaged tyrosine (section 4).^144^

**Figure 4:**
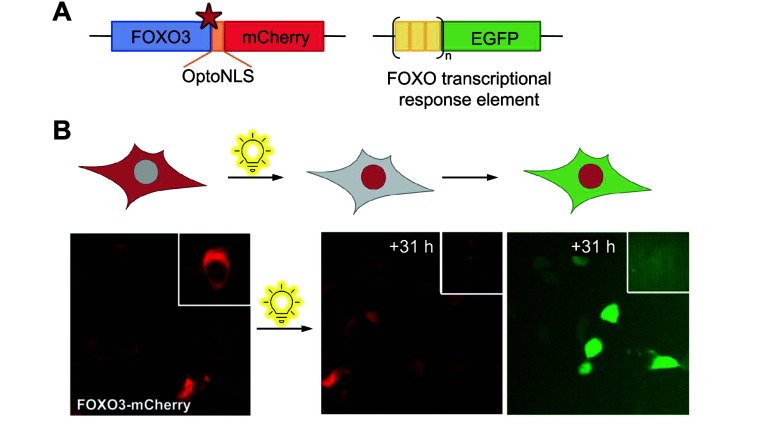
Optical control of localization using photocaged lysine. (A) Gene design for fluorescence tracking of FOXO3 localization. FOXO3 is linked to mCherry by an OptoNLS sequence bearing photocaged lysine. Nuclear localization of FOXO3 is detected with an EGFP reporter bearing tandem repeats of FOXO3 transcriptional response elements. (B) Prior to irradiation, FOXO3-mCherry remains in the cytoplasm. Decaging of the OptoNLS yields nuclear localization and subsequent transcriptional activation of GFP. Reproduced with permission from ref (134). Copyright 2014 American Chemical Society.

Another early approach to activating gene expression through light exposure involved optically controlling transcription factor localization. Three nuclear localization sequences (NLS) were caged with **PCK** or **ONBK** for temporal control over protein translocation into the nucleus: 1) the NLS of SatB1 (termed OptoNLS), 2) the NLS of TDP-43, and 3) the bipartite NLS of p53.^68,134^ OptoNLS was used to control nuclear localization of FOXO3, a transcription factor regulating apoptosis and implicated in tumorigenesis.^134^ The OptoNLS is designed such that the NLS is situated between a gene of interest (EGFP, FOXO3, etc.) and mCherry, with **PCK** inserted at the critical K29, blocking translocation until irradiation (Figure 4A). Optical control over nuclear import of FOXO3-OptoNLS-**PCK**-mCherry was demonstrated through mCherry localization to the nucleus after 120 s of irradiation and subsequent, slower activation of EGFP expression from an upstream FOXO transcriptional element (Figure 4B). Notably, quantitative translocation of FOXO3-OptoNLS corresponds to quantitative decaging, demonstrating a unique aspect of optically controlled nuclear localization compared to caged enzymes.

Similarly, TEV protease was fused to OptoNLS to optically control protein cleavage. Initial mislocalization of substrate and enzyme to two different cellular compartments prevented substrate cleavage, but simple irradiation induced nuclear import, and proteolysis of the now colocalized substrate was observed.^134^ A nuclear-localized mCherry-SATB1^TEV^-GFP fusion was used as a reporter for TEV protease activity. Upon irradiation and nuclear localization of the protease, mCherry is cleaved from GFP, leading to diffusion of the fluorescent protein throughout the cell, which is easily imaged. Importantly, no background cleavage was observed in the absence of light exposure. Thus, this approach could be paired with any protein that is exclusively functional in the nucleus.

OptoNLS was also fused to Yes-associated protein (YAP) to study its role in tumor invasion.^135^ Although high nuclear YAP levels have been associated with tumor invasion, it was unknown whether nuclear localization caused this behavior or was merely a correlation.^175^ To study this process, YAP was fused to OptoNLS (optoYAP), and irradiation of tumor spheroids containing optoYAP led to the display of a larger number of invading cells than the nonirradiated control.^135^ Selective irradiation in a specific area of the spheroid also exclusively led to localized invasion, further demonstrating that nuclear localization of YAP was essential to trigger tumor invasion.

Another NLS, derived from TDP-43, was also caged with **ONBK**.^136^ TDP-43 is a DNA/RNA binding protein involved in regulation of RNA processing.^176^ Although a primarily nuclear protein, it is localized to the cytosol in times of cellular stress, where it forms protein condensates.^177^ To study these condensates, K48 in the NLS of the protein was caged with **ONBK**.^136^ Acetylation of this residue is known to prevent TDP-43 nuclear localization, so addition of the bulky caging group at this position was predicted to have the same effect.^136,178^ Using a caged TDP-43-mRuby fusion reporter, nuclear import was shown to be dependent upon irradiation. The condensates formed by nonirradiated caged protein were studied using fluorescence recovery after photobleaching (FRAP) experiments, which revealed that the condensates included insoluble aggregates as well as more liquid-like condensates.^136^

Finally, the bipartite NLS of the tumor suppressor p53 was also caged at the key lysine residue K305, which is located in the basic domain of the NLS.^68^ This site is required for binding of importin α, the nuclear import factor that binds the NLS in the first step of nuclear translocation.^179^ This **PCK**-caged NLS was not as tightly controlled as OptoNLS and suffered from notable leakage into the nucleus before decaging; however, nuclear transport after decaging occurred within seconds and the precise starting time point allowed for kinetic measurements.^68^ Overall, these studies demonstrate that a photocaged NLS can act as a broadly applicable tool for controlling the activity of any nuclear-active protein.

**Figure 5:**
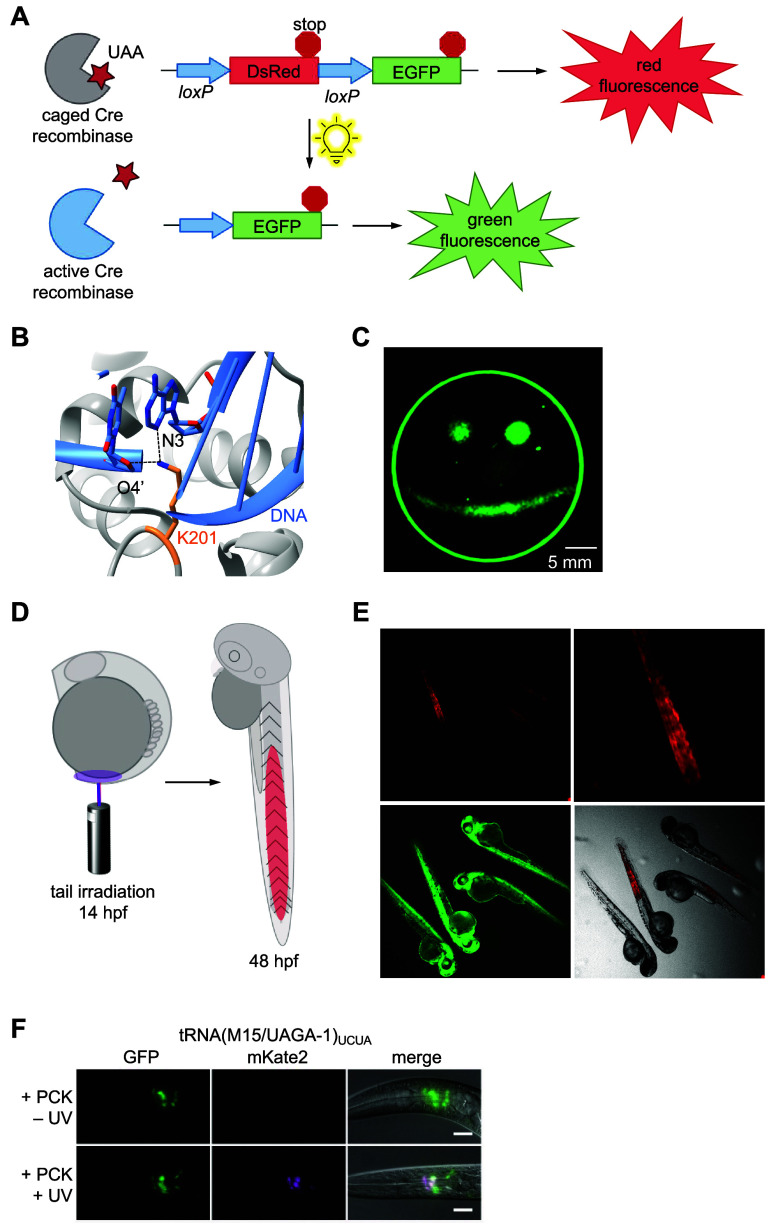
Photocaged Cre recombinase. (A) The Cre-stoplight reporter features a DsRed gene followed by a stop codon and flanked by loxP sites upstream of an EGFP gene. Without Cre-mediated recombination, DsRed is expressed from this reporter. Excision of the DsRed gene fragment by Cre recombinase yields EGFP expression.^148,183^ (B) Chimera rendering of the active site of Cre recombinase where K201 (orange) coordinates O5′ (not shown) and O4′ of the −1 sugar and N3 of the +1 base of loxP DNA (blue; PDB 1Q3V).^149^ (C) Spatial control of Cre activity through patterned irradiation of cells expressing Cre-Stoplight-K201PCK. Reproduced with permission from ref (148). Copyright 2016 The Royal Society of Chemistry under CC BY 3.0, https://creativecommons.org/licenses/by/3.0/. (D) Cartoon depiction of cell lineage tracing through irradiation of a zebrafish embryo expressing HCK-caged Cre-recombinase. Note that the EGFP and RFP genes are swapped in the design of this reporter compared to (A). (E) Irradiation of the embryonic tail 14 hpf leads to Cre recombinase activation in the tail of the embryo within 48 hpf. Panels (D,E) reproduced with permission from ref (149). Copyright 2018 Wiley-VCH Verlag GmbH & Co. KGaA, Weinheim. (F) Quadruplet directed incorporation of **PCK** into Cre recombinase for optical activation of a channelrhodopsin:mKate2 reporter in *C. elegans*. Reproduced with permission from ref (172). Copyright 2022 Oxford University Press under CC BY 3.0, https://creativecommons.org/licenses/by/3.0/.

Another prominent target of lysine photocaging for optical control over gene expression has been Cre recombinase, an enzyme that can lead to the insertion, deletion, or inversion of a DNA sequence encoded between two *loxP* sites, each containing 13-base pair sequences flanking an 8-base pair asymmetric sequence (Figure 5A).^148,149,167,171,172^ Cre recombinase has been widely used as a genome engineering tool for studying gene function, and optical control provides an additional layer of precision by providing temporally and spatially restricted DNA recombination events and thereby gene expression.^180,181^ Photocaging of Cre recombinase using tyrosine and histidine analogues is also discussed in sections 4 and 7, respectively. The enzyme has a critical active site lysine, K201, that is highly conserved across the Int family of recombinases and is required for enzyme activity.^148^ K201 interacts with the minor groove of the target DNA, forming hydrogen bonds with the 5′- and 4′-O of the −1 sugar and the N3 of the +1 guanine base (Figure 5B).^148,149,182^ Cre recombinase has been caged in mammalian cells, zebrafish, and *C. elegans* through the incorporation of photocaged lysines at K201.^148,149,167,171,172^ Caging of this lysine was first demonstrated in HEK293T cells through the introduction of **PCK** at this site and confirmed through use of a Cre-stoplight reporter, in which a DsRed gene is followed by a stop codon and flanked by *loxP* sites on either side, all upstream of an EGFP gene (Figure 5A).^148,183^ This reporter therefore produces red fluorescence in the absence of Cre-mediated recombination and green fluorescence after DsRed excision by the recombinase. Precise spatiotemporal control over Cre recombinase activation was demonstrated through patterned irradiation of a layer of HEK cells (Figure 5C).^148^ Further, tuning of DNA recombination based on light exposure was demonstrated by a longer irradiation of the right eye of the smiley face than the left eye.

Utilizing the coumarin lysine, **HCK**-photocaged Cre recombinase was used to track cell differentiation in zebrafish embryos (Figure 5D).^149^ By selectively irradiating specific cells at early stages of development in zebrafish embryos, the differentiation of these early cells and the tissues that they form can be traced to their final identity during development by tracking their fluorescence using a Cre-stoplight-like reporter with an EGFP gene followed by an mCherry gene (Figure 5E).^149,183^ As the reporter is integrated into the zebrafish genome, the genomic modification by Cre recombinase in the selectively irradiated cells is passed down to their daughter cells, allowing for the progeny of these cells to be lineage traced.

Another application of photocaged Cre recombinase used **PCK**-caged enzyme to elucidate the roles played by individual cells in neuronal pathways of *C. elegans*.^167^ The gene encoding for Chrimson, an optogenetic ion channel, was placed after a transcriptional terminator that was located between two *loxP* sites so that the channel would be expressed in the presence of active Cre recombinase. The individual neurons PLMR or PLML, the right or left posterior lateral microtubule (PLM) touch sensory neurons of *C. elegans*, respectively, were then irradiated to selectively activate DNA recombination in one cell at a time and lead to bilateral Chrimson expression. These individual neurons could then be activated by 470 nm irradiation of the Chrimson optogenetic channel to determine the difference in behavioral responses caused by just one or both neurons being activated. This methodology has broad applications as it provides a novel means of probing the roles played by individual neurons in pathways of interest.

In a second approach to genetic code expansion in *C. elegans*, a quadruplet codon, UAGA, instead of the more common UAG triplet codon, was suppressed to incorporate **PCK**.^172^ Incorporation was attempted using different combinations of mutated PylT scaffolds and anticodon loops. A specific quadruplet tRNA, tRNA(M15/UAGA-1), led to incorporation rates of 30–50% of those seen with triplet tRNA. Although not as successful as classic triplet genetic code expansion, the use of quadruplet codons allowed the use of multiple ncAAs in one organism while preventing native UAG codons from leading to the incorporation of an ncAA. The success of this optimized quadruplet tRNA was proven through the incorporation of **PCK** into the active site of Cre recombinase and testing of its activity with a previously used channelrhodopsin:mKate2 reporter, which resulted in red fluorescence upon UV irradiation and Cre-mediated recombination (Figure 5F). These experiments excitingly showed that tRNA(M15/UAGA-1)_UCUA_ was as successful in generating red fluorescence in animals as the most successful triplet tRNA, tRNA(C15)_CUA_, with around 70% of the specimens displaying red fluorescence when irradiated. This work represents the first time a quadruplet codon was used for genetic code expansion in a multicellular organism and was also applied to encode a photocaged cysteine in *C. elegans* (section 5).

**Figure 6:**
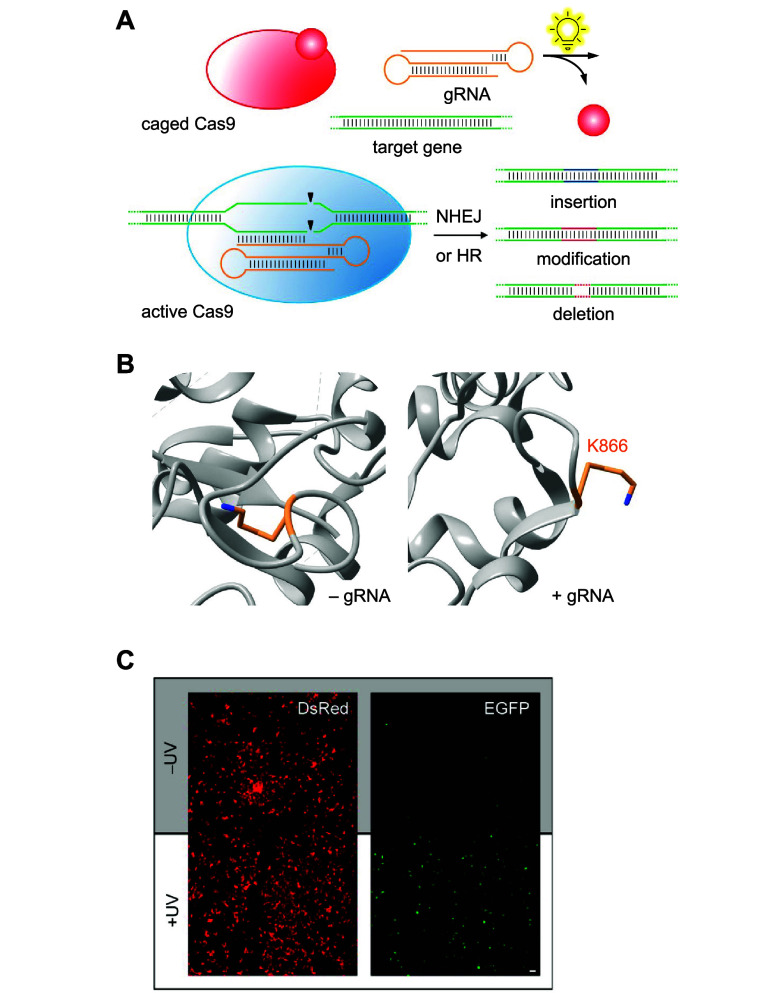
Photocaged CRISPR/Cas9. (A) The CRISPR/Cas9 system is able to produce insertions, modifications, or deletions in the genome through nonhomologous end joining (NHEJ) or homology-directed repair (HR). (B) Conformational change of Cas9-K866 after gRNA binding, orienting the lysine toward the protein surface (PDBs 4CMP and 4OO8). (C) Spatial activation of a Cas9-K866**PCK** as observed through a Cas9-controlled dual reporter. Panels (A,C) are reproduced with permission from ref (112). Copyright 2015 American Chemical Society.

To optically control protein expression through gene editing, a light-activated Cas9 was developed.^112^ Type II CRISPR systems feature a CRISPR RNA (crRNA) and a trans-activating crRNA (tracrRNA) that can be combined into a single guide RNA (gRNA).^184−186^ Upon sequence recognition, Cas9 cleavage of the target DNA occurs, followed by nonhomologous end-joining (NHEJ) or homology-directed repair (HR), allowing for insertion, modification, or deletion of a target sequence in the genome (Figure 6A). The CRISPR/CAS9 system has been widely utilized for targeted genome editing.^184−186^ Five lysine residues in Cas9 were identified as potential targets for caging.^112^ Four of them, K76, K163, K510, and K742 are each highly conserved between different Cas9 isoforms and are located in close proximity to the gRNA binding site.^187^ A fifth, K866, undergoes a large conformational change upon gRNA binding to orient the lysine toward the surface of the protein, suggesting that it might be necessary for target DNA cleavage (Figure 6B). Utilizing a reporter construct that switches from DsRed to EGFP expression upon interaction with Cas9 and matching gRNAs, a **PCK** scan at these five positions was performed. K866 was identified as a critical lysine, as mutation to **PCK** inactivated the enzyme (Figure 6C).^112^ Notably, no background activity was seen before UV exposure. Optical control over silencing of endogenous genes was also demonstrated for transmembrane transferrin receptor CD71 upon UV activation of caged Cas9 in the presence of the appropriate gRNAs. These results open the door to numerous applications of a photocaged CRISPR/Cas9 system, such as placing sequence-specific gene insertions, modifications, or deletions under optical control in cells and multicellular model organisms whose genetic code has been expanded.^184,188^

Another prominent application of photocaged lysine has been optical control over post-translational modifications (PTMs). PTMs are alterations to amino acid side chains after protein translation has been completed, and are crucial to a wide range of cellular processes.^189,190^ Modifications are installed by enzymes called “writers”, recognized by “readers”, and removed by “erasers”.^189^ Writers have been the main target for introduction of spatiotemporal control, as they modify proteins with structurally diverse groups such as phosphates, sulfonates, acetyl groups, methyl groups, etc. Another method to control PTMs is through caging of the protein substrate, either at modified side chains or at residues that perform key interactions with “writers” to further understand the downstream effects of these PTMs.

**Figure 7:**
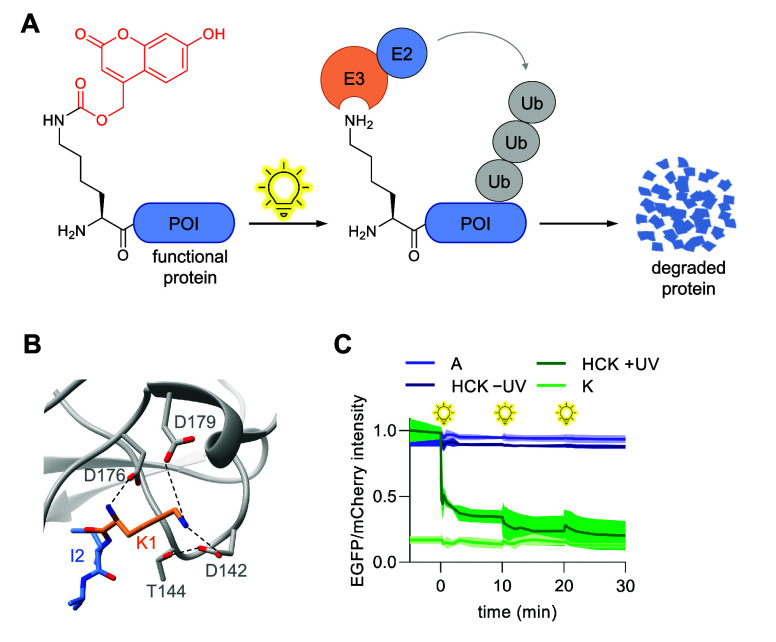
Photocontrol over N-end degradation. (A) Photocaged lysine **HCK** prevents recognition of the N-terminal lysine by E3 ligase in the N-end degradation pathway. After irradiation, the lysine is regenerated, and E3 ligase is recruited, leading to ubiquitination and protein degradation. (B) Crystal structure showing key interactions of K1 with the UBR box of UBR1 (PDB 3NII). (C) OptoDeg-fused EGFP is selectively degraded relative to the mCherry internal control. Panels (A,C) are reproduced with permission from ref (162). Copyright 2021 American Chemical Society.

**Figure 8:**
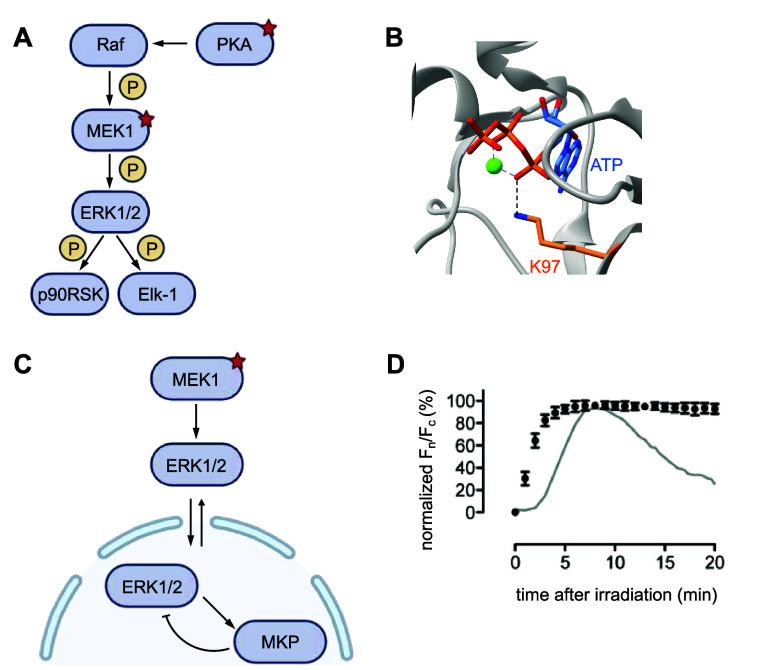
Optical control of cell signaling through the Raf/MEK/ERK pathway. (A) Diagram of the core Raf/MEK/ERK signaling pathway.^123,200^ (B) Rendering of key residue K97 in MEK1 (PDB 1S9J). (C) MEK1 phosphorylates ERK1/2, which is transported from the cytoplasm to the nucleus. ERK1/2 phosphorylates MKP, which in turn dephosphorylates ERK. (D) Nuclear over cytosolic fluorescence intensity over time shows nuclear transport within 2 min. The gray line represents cells consistently stimulated with EGF. Reproduced with permission from ref (123). Copyright 2011 American Chemical Society.

For example, the N-terminal lysine of a peptide degron was caged with **HCK** to introduce photocontrol over the N-end degradation pathway, which can determine the half-life of a protein by the identity of its N-terminal residue(s).^191^ Proteases in mammalian cells can expose a natural N-degron, typically a terminal lysine, which destabilizes the protein and initiates degradation through recognition by a UBR class E3 ligase for subsequent ubiquitination and degradation by the proteasome (Figure 7A).^191−194^ Specifically, this N-terminal lysine undergoes electrostatic interactions with the negative residues in the binding pocket of the E3 ligase (Figure 7B). Caging this terminal lysine with **HCK** therefore interrupts these electrostatic interactions, as well as introduces steric bulk that interferes with E3 ligase, specifically UBR1, recruitment.^162^ Degradation of the protein is then dependent on irradiation with 365 or 405 nm light. Optical control of this caged degron, termed OptoDeg, was demonstrated through degradation of OptoDeg-fused Fluc, EGFP (Figure 7C), MKP3 (a phosphatase in the ERK/MAPK signaling pathway; also see Figure 8C) and MEK1 (a protein kinase in the Ras/MAPK signaling pathway; also see Figure 8A) selectively upon irradiation. Control over MKP3 and MEK1 highlights the ability of the OptoDeg to regulate cellular pathways upon irradiation without the need to specifically design photocaged analogues of proteins in each pathway of interest, as OptoDeg can be encoded on the N-terminus of different proteins of interest without further engineering.

Another category of photocaged lysines include those that generate *N*-methyl-lysine upon decaging.^71,72^ Lysine methylation is a prominent PTM that controls protein activity, stability, localization, and interactions, and is especially critical for its role in gene expression through histone methylation.^71^ However, it would be difficult to directly incorporate an *N*-methyl-lysine using genetic code expansion, as it would be challenging to obtain a synthetase that would not also recognize native lysine.^72^ Thus, the *o*-nitrobenzyl caged methyl-lysine, **MeONBK,** was synthesized and an *Mb*PylRS mutant for incorporation of **MeONBK** was identified through a double-sieve selection. The synthetase, MeONBKRS (Table 1), was able to selectively incorporate **MeONBK** into myoglobin in *E. coli* and EGFP in CHO cells, as confirmed by mass spectrometry.^71^ An alternative *Mm*PylRS, MeONBKRS2 (Table 1) was also identified through a selection with a library of mutants with six residues randomly mutated.^71,72^ The function of MeONBKRS2 was confirmed through incorporation of **MeONBK** into GFP in *E. coli* and further verified by mass spectrometry.^72^ Future applications investigating site-specific lysine methylation on protein function with temporal precision are expected.

PTMs, especially phosphorylation and dephosphorylation, play a major role in cell signaling cascades.^195^ While signaling cascades are the backbone of cell decision making in response to external stimuli, their high complexity hinders full understanding of these intricate networks.^123^ The introduction of photocontrol into cell signaling cascades allows for investigation into the connections and kinetics behind these complex networks. Kinases, writers of phosphorylation, have been a frequent target of photocontrol, as the phosphorylation of proteins and lipids is a key method of communication within these cascades.^70,120,121,123^ Many methods for regulation of kinase activity have been developed, such as small molecule inhibitors, chemical rescue of an inactivating mutation, destruction of a kinase inhibitor, and activation through an installed rapamycin binding domain.^123,196−199^ However, many methods of investigation into kinase activity are performed *in vitro*, preventing the full complexity of cellular interactions from impacting results.^121^ Introducing optical control to the kinase activation toolbox allows for an alternate approach for rapid and specific activation of individual kinases in cells to determine the roles they play in signaling pathways and the kinetics of these interactions. To see examples of other photocaged kinases, see sections 4, 5, and 8.

For example, the kinase MEK1, a key member of the Raf/MEK/ERK signaling pathway, has been caged with both **PCK** and **HCK**.^123,124^ Natively, the Raf/MEK/ERK cascade is triggered by external stimuli such as growth factors and leads to final cellular responses like cell proliferation or differentiation.^200^ MEK1 is activated by phosphorylation by Raf, and in turn activates kinases ERK1/2 through phosphorylation (Figure 8A).

In one approach to optically control MEK1, a lysine that is highly conserved across the kinome, K97, was identified and photocaged.^123^ The applicability and transferability of caging conserved lysine K97 created a tool that could allow optical control of activity of almost any protein kinase of interest.^123,201^ K97 is located in the ATP binding pocket of the enzyme and helps to bind and orient the ATP (Figure 8B), and is thus required for phosphorylation to occur.^123,124,201^ Optical control via **PCK** incorporation into a constitutively active MEK1 mutant, in which residues 30–49 are deleted (A-MEK1-ΔN), was confirmed in HEK293ET cells.^123^ Increasing irradiation times of MEK1-K97**PCK** were shown to restore phosphorylation of an EGFP-ERK2 fusion, proving successful optical control of the protein. To further validate photoactivation of MEK1 and downstream signaling, phosphorylation of ERK1/2 substrates, the p90 ribosomal S6 kinase (p90RSK) and the transcription factor Elk-1, were probed and shown to only be phosphorylated after irradiation.

The power of this tool was demonstrated through its use elucidating the behavior of the Raf/MEK/ERK signaling pathway; specifically, in confirming the hypothesis that upstream factors, such as Raf desensitization, are responsible for the sigmoidal kinetics of ERK1/2 nuclear localization.^123^ Before phosphorylation by MEK1, ERK1/2 is bound to a series of cytoplasmic anchors including MEK1 itself.^123,202^ ERK1/2 then localizes to the nucleus upon dual phosphorylation, but after increasing nuclear accumulation upon added stimuli, nuclear levels of ERK1/2 eventually plateau and fall back to resting levels as more protein is dephosphorylated by nuclear MAPK phosphatases (MKPs) than is phosphorylated by MEK1 (Figure 8C), even with continued external stimuli such as exposure to epidermal growth factor (EGF).^123,202^ The desensitization to continued stimulus was hypothesized to be dependent on factors upstream of MEK1 and ERK1/2.^203^

To confirm this hypothesis, a different constitutively active MEK1 mutant bearing dual serine to aspartate mutations to mimic phosphorylation (see also Figure 39B), C-MEK1-DD, was caged with **PCK** at K97.^123^ After irradiating C-MEK1-DD-**PCK** with 365 nm light, confocal fluorescence imaging confirmed significant nuclear localization of EGFP-ERK2 after 10 min at a rate 3-fold greater than that of EGF-stimulated localization (Figure 8D). Through optical control of MEK1, nuclear localization was also sustained for long periods and did not display the typical desensitization to continued stimuli. This discovery supports the above proposed hypothesis, as photoactivation of the pathway through direct activation of MEK1 is free from influence by upstream factors such as Raf, suggesting these upstream factors are necessary for desensitization to occur. The success of kinase perturbation through caging of a conserved, active site lysine elucidated the behaviors of this complex signaling pathway and demonstrated the potential of this tool for expanding the knowledge of other cell signaling pathways.

MEK1 was also caged with **HCK** at K97 (Figure 8B) in zebrafish.^124^ MEK/ERK signaling is known to play a role in establishing dorsal and ventral polarity in early embryo development.^204^ Caging of MEK allowed it to be optically activated at different time points in embryogenesis, providing temporal precision for the identification of a specific developmental window in which the MEK/ERK pathway establishes polarity in the embryo.^124^ This approach also has the potential to be generalized to study the effects of activating different pathways at different points in zebrafish development.

**Figure 9:**
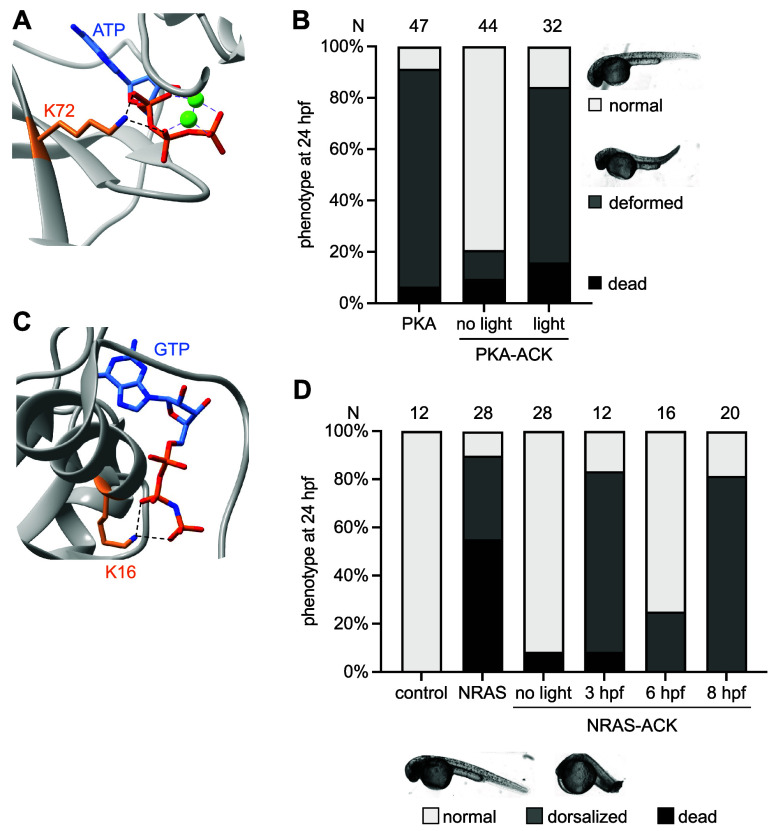
Optical control of PKA function in zebrafish. (A) The critical residue K72 in PKA interacts with the α- and β-phosphates of ATP (PDB 4WB5). (B) Zebrafish embryos expressing PKA-K72**ACK** were irradiated at 4 hpf and imaged 24 h later. A distinct body axis defect was observed only when PKA was decaged. (C) Residue K16 of NRAS has key electrostatic interactions with the β- and γ-phosphates of GTP (PDB 5UHV). (D) Embryos injected with mRNA encoding NRAS-K16**ACK** had a decreased dorsalization phenotype compared to the NRAS-G60E positive control (NRAS), which was then regenerated following decaging of NRAS-K16**ACK**. Panels (B,D) are reproduced with permission from ref (70). Copyright 2023 American Chemical Society.

The RAS/MAPK pathway was further placed under optical control through the caging of protein kinase A (PKA) (Figure 8A), a GTPase activator of Raf and NRAS.^70^ The aminocoumarin lysine **ACK** was used to cage K72 in PKA, a critical lysine that interacts with the α- and β-phosphates of ATP (Figure 9A).^205^ Caged PKA was expressed in zebrafish embryos in order to investigate the roles of PKA in embryonic development.^70^ Injection of mRNA for a constitutively active form of PKA eliminated the necessity of upstream activation and led to body axis defects in zebrafish embryos (Figure 9B). When injected with mRNA encoding for PKA-K72**ACK**, however, the embryos retained a normal phenotype until irradiated 4 h post fertilization (hpf). Following **ACK** decaging, phenotypes returned to the same body axis defect exhibited when uncaged PKA was expressed.

Similarly, NRAS was caged with **ACK** at K16, a key lysine that binds and orients GTP by forming hydrogen bonds with the two oxygens in the β- and γ- phosphates of GTP (Figure 9C).^70,206^ NRAS activates the RAS/MAPK pathway when bound to GTP, but hydrolysis of GTP to GDP inactivates NRAS binding to downstream signaling nodes.^207,208^ A mutant NRAS, G60E, is known to decrease binding between the protein and its assistant protein, GTPase activating protein (GAP), in human RASopathies.^209−211^ This therefore increases RAS/MAPK signaling and has been shown to cause dorsalization defects in zebrafish.^70,211^ Irradiating zebrafish embryos expressing caged protein at different times post fertilization revealed that defects only occurred if NRAS was activated early after fertilization, revealing that the G60E mutation must also lead to RASopathy characteristics such as heart defects during early embryo development (Figure 9D).^70^ These results demonstrate the use of **ACK** to study the mechanisms through which embryonic defects develop, which could aid in the development of therapies for these defects. Similarly, optical control of NRAS caged with **HCK** at K16 has also been demonstrated in *Xenopus laevis* embryos.^106^

**Figure 10:**
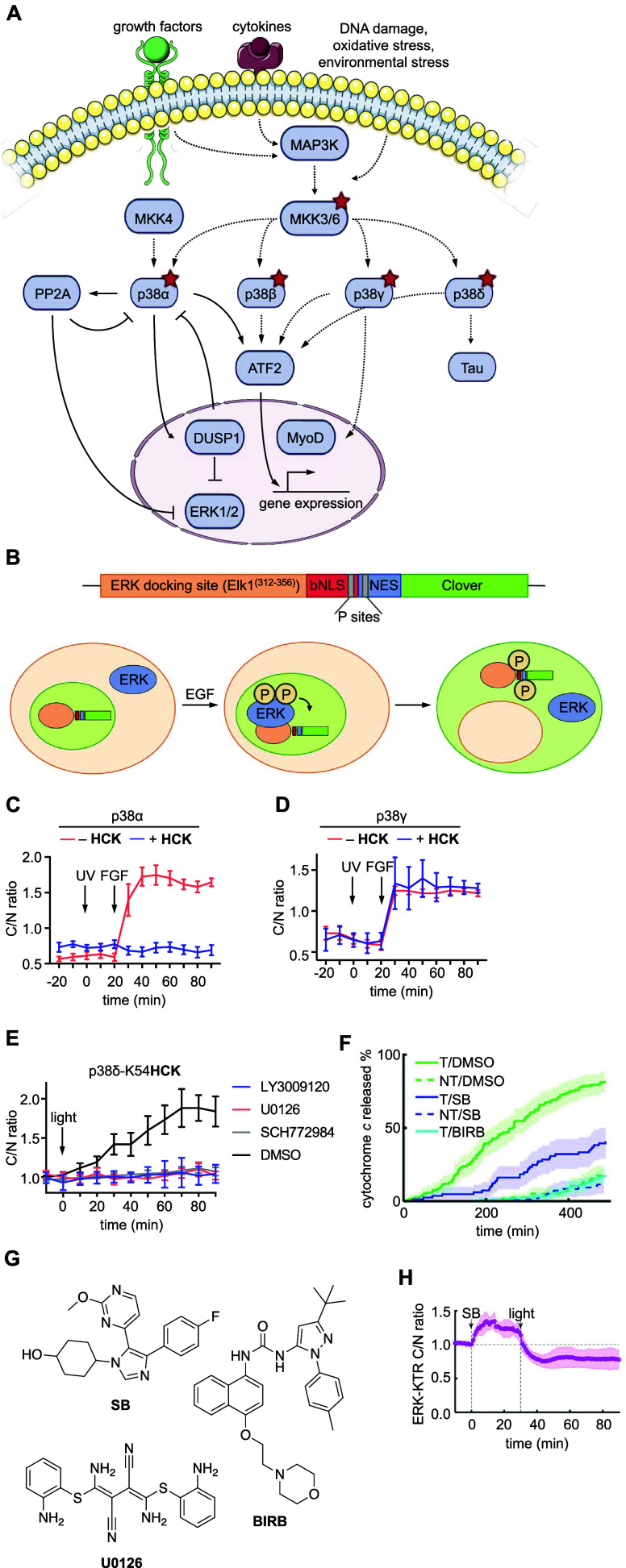
Optical control of MKK6 and different isoforms of p38. (A) Stimulation by external factors initiates the MKK6 and p38 signaling cascades. Reproduced with permission under Creative Commons license from ref (127). Copyright 2023 Royal Society of Chemistry. (B) The ERK-KTR reporter translocates to the cytoplasm upon phosphorylation by ERK. Reproduced with permission from ref (217). Copyright 2014 Elsevier Inc. (C) Time course quantification of ERK-KTR-Clover localization following photoactivation and FGF stimulation of p38α and (D) p38γ. (E) Small molecule inhibition of the ERK pathway suppressed crosstalk between p38δ and ERK, as measured through reporter translocation. Panels (C,D,E) reproduced with permission from ref (127). Copyright 2023 The Royal Society of Chemistry. (F) Cytochrome *c* is released from cells expressing MKK6-**PCK** (T) after UV-exposure and is decreased in the presence of inhibitors SB 239063 (**SB**) or BIRB 796 (**BIRB**). NT indicates nontransfected cells. (G) Chemical structures of small molecule inhibitors used in tandem with p38 and MKK6 photoactivation. (H) Quantification of ERK-KTR-Clover translocation from the cytoplasm to the nucleus when cells were first treated with p38α/β specific inhibitor SB 239063 (**SB**), then irradiated with UV light. Panels (F,H) reproduced with permission from ref (125). Copyright 2020 Elsevier under CC-BY license.

Isoforms of signaling proteins can play different or overlapping roles within a signaling pathway and may have varying regulatory mechanisms or downstream targets.^212^ These differences are often difficult to study, as development of isoform-specific inhibitors or activators can be elusive. The MAPK signaling protein p38, for example, has four isoforms, although their differences in function have not been well-understood.^213^ Introduction of **HCK** into each isoform allowed selective perturbation of activity to study interactions with other members of the p38/MAPK pathway, as well as to track crosstalk with proteins in other MAPK cascades such as the MEK/ERK pathway (Figure 10A).^127^ Photocaging with **HCK** was performed at a conserved lysine in the ATP-binding pocket of all four isoforms: K53 in p38α and p38β, K56 in p38γ, and K54 in p38δ.^214,215^ Introduction of **HCK** at these residues sterically blocks ATP from entering the active site, as well as removes the positive charge of the native lysine, preventing electrostatic interaction with the α-phosphate of ATP as well as a stabilizing salt bridge interaction with the conserved residue E71 of the protein.^216^ Caging the critical lysine prevented activity in all four isoforms until irradiated, as shown by Western blot of the phosphorylated substrate ATF2 and by ERK-KTR-Clover reporter activity (Figure 10B-D).^127^ This reporter consists of the ERK binding domain of Elk-1, an NLS, and a nuclear export signal (NES), so that upon activation and nuclear localization of ERK, phosphorylation of the reporter leads to activation of the NES, deactivation of the NLS, and resultant cytoplasmic localization of the Clover fluorescent signal.^217^

Notably, investigation of the effects of selectively activating individual isoforms of p38 revealed their differing roles: while p38α/β selectively activated the p38/MAPK pathway, p38γ/δ only activated the ERK/MAPK pathway, providing the first evidence of positive ERK crosstalk by p38.^127^ Further investigation into the signaling node at which the crosstalk occurred was conducted using small molecule inhibitors for different signaling proteins in the ERK/MAPK pathway, including Raf, MEK, and ERK (Figure 10E).^127^ Ultimately, inhibition of any of these nodes prevented crosstalk, leading to the conclusion that crosstalk must occur at, or further upstream than Raf. The unique temporal control of photocaged signaling proteins provides complete genetic specificity over interactions with proteins in other pathways. When combined with small molecule inhibitors, this approach presents the potential for a broadly applicable method to better understand the dynamic interactions between signaling pathways in cells.

The p38/MAPK pathway was further probed through caging of MAP kinase kinase 6 (MKK6) with **PCK** at the conserved active site lysine K82.^125^ MKK6 phosphorylates p38 (Figure 10A), which is known to result in apoptosis.^218^ Acute temporal control over MKK6 activation allowed investigation into the kinetics of p38 phosphorylation, as well as location of the points of crosstalk between the p38/MAPK pathway and other MAPK cascades,^125^ while constitutive activation would lead to cell death. Apoptosis, characterized by cytochrome *c* release from the mitochondria into the cytoplasm,^219^ was observed after optical activation of MKK6-**PCK** (Figure 10F-G) but suppressed through administration of a p53 inhibitor,^125^ confirming that MKK6-triggered apoptosis is p53-dependent.

To study the effect of MKK6 activation on the Raf/MEK/ERK pathway, activity of ERK kinase was first shown to be downregulated upon MKK6-**PCK** decaging using an ERK kinase translocation reporter (Figure 10B).^125^ This interaction was further studied by addition of the p38 inhibitor **SB** after MKK6 decaging, which unexpectedly did not lead to the recovery of ERK activity (Figure 10H).^125^ Addition of the p53 inhibitor before MKK6 activation still led to negative regulation of ERK activity, proving that MKK6 regulates the ERK pathway in a p38-independent manner, a previously unknown interaction. These results were also corroborated with an MKK6 caged at the same position with **HCK**.^126^ The combination of optical control to activate a member of a pathway and small molecule inhibition to prevent the activity of another provides a method with great potential to investigate the intricacies of cell signaling kinetics and interactions.

Another kinase that has been placed under optical control through the introduction of photocaged lysine is the lymphocyte-specific protein tyrosine kinase (LCK).^121^ LCK is a member of the SRC family of tyrosine kinases involved in T-cell receptor signaling.^121,220^ T-cell receptor (TCR) binding to major histocompatibility complex proteins on the surface of host cells triggers phosphorylation of the intracellular regions of the TCR by LCK, which then triggers recruitment and subsequent phosphorylation of ZAP70, a tyrosine kinase.^221^ LCK was caged with **PCK** at K273, an active site lysine required for substrate phosphorylation.^121,222^ After caging, no phosphorylation of ZAP70 was seen via phospho-western, while irradiation with 350–400 nm light restored phosphorylation in a direct relationship with the duration of irradiation.^121^

Additionally, adenylate kinase was caged with **HCK** to generate an optically controlled regulator of cellular ATP levels.^120^ Adk is a critical enzyme involved in the interconversion of adenine nucleotides, the regeneration of ATP in the cell from ADP, as well as *de novo* synthesis of adenine nucleotides and phospholipid biosynthesis.^223^ By photocaging at K13, a critical lysine involved in orienting the γ-phosphate of ATP, that is required for enzyme activity, ATP binding is blocked.^120^ Caged Adk was shown to prevent ATP homeostasis in an *E. coli* strain with thermosensitive endogenous Adk until irradiation and subsequent decaging. Similarly, ATP levels were shown to decrease dramatically in mammalian (HEK293T) cells upon Adk-K13**HCK** decaging, demonstrating the extensive potential of this tool for perturbation of cellular ATP levels.

**Figure 11:**
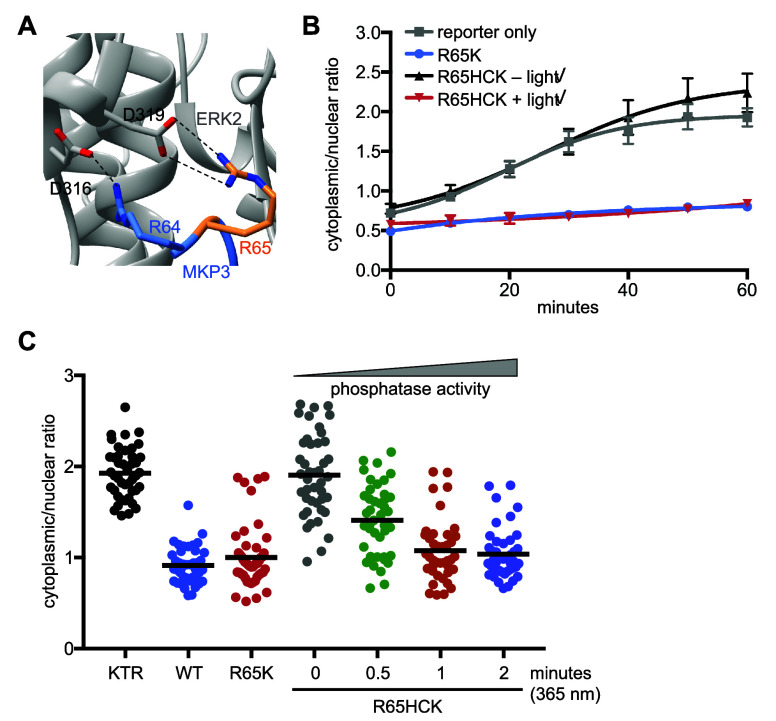
Optical control of MKP3. (A) Electrostatic interactions between ERK2 (D316 and D319, gray) and R64 (blue) and R65 (orange) of the kinase interaction motif of MKP3 (blue) are necessary for MKP3 activity (PDB 2FYS). (B) MKP3-R65**HCK** cannot dephosphorylate pERK until UV irradiation, as quantified via nuclear export of an ERK-KTR reporter. (C) Photolysis of MKP3-R65**HCK** shows an irradiation-dependent response of pERK translocation. Panels (B,C) are reproduced with permission from ref (224). Copyright 2019 Springer Nature under CC-BY license.

Another enzyme critical in cell signaling, the dual specificity phosphatase 6 (DUSP6 or MKP3), was also caged with **HCK**.^224^ MKP3 dephosphorylates dually phosphorylated ERK (see Figure 7C).^225^ Photocaging was used to investigate the effect of MKP3 on ERK activity, specifically to study if the phosphatase completely blocks ERK signaling after accumulating above a certain concentration in a switch-like mechanism, or if MKP3 dampens stimulation in a dose-dependent fashion. To study this question, MKP3 was caged at its interface with ERK2 through the introduction of **HCK** at R65, a residue that undergoes an electrostatic interaction with negatively charged D319 of its substrate, ERK2 (Figure 11A).^224^ Although most other caged signaling enzymes have been caged at lysine-phosphate interfaces, caging at the protein–protein interface of the phosphatase provides new opportunities to expand the caging approach. Optical control of DUSP6 activity was demonstrated (Figure 11B) using an ERK-KTR translocation reporter (Figure 10B). The effect of different concentrations of active MKP3 in the cell was then studied using different irradiation times of the caged phosphatase, which uniquely allows for the titration of activated enzyme (Figure 11C). The cytoplasmic/nuclear ratio of the reporter progressively decreased after longer irradiation times, showing that MKP3 displays a graded response in the regulation of ERK signaling. In an alternative approach, MKP3 was also caged with a cysteine derivative (section 5).

Based on the success of caging MKP3, another phosphatase, Src-homology 2 (SH2) domain-containing protein tyrosine phosphatase 2 (SHP2), was also photocontrolled through introduction of **HCK**, further demonstrating general applicability of the approach.^140^ SHP2 includes a catalytic protein tyrosine phosphatase (PTP) domain and two SH2 domains (C-SH2 and N-SH2).^226^ SH2 domains recognize and bind to phosphotyrosine motifs and are found in a variety of proteins involved in cell signaling.^227,228^ In the closed, inactive form of SHP2, the N-SH2 domain sterically blocks the PTP domain, but upon SH2-mediated phosphotyrosine binding, the enzyme moves to its open conformation and the PTP domain is accessible.^229^ R138 in the C-SH2 domain of the enzyme undergoes an electrostatic interaction with the phosphate of pTyr to facilitate this interaction.^230^ An ERK KTR-mCherry (similar to the reporter described in Figure 10B) was used to visualize the effects of the phosphatase on the ERK pathway.^217^ SHP2 activates ERK signaling through Ras-GDP dephosphorylation and subsequent Raf recruitment.^231^ Using an ERK-KTR-mCherry reporter, SHP2-R138 K was shown to have a similar activating effect on ERK signaling as expression of wild-type SHP2, suggesting that the lysine scar left from decaging of **HCK**138 would not affect enzyme activity.^140^ Caging of this residue with **HCK** resulted in an inactive SHP2, as no ERK signaling was detected in serum starved cells. Irradiation then showed reporter activation through triggering of ERK function. This method of caging an allosteric site that undergoes phosphotyrosine interaction has the potential to be applied to other proteins with SH2 domains. SHP2 was also light-activated through introduction of a caged tyrosine derivative (see section 4).

Similarly, a different SH2 domain was caged with **ONBK**.^164^ Two positively charged arginine residues in the SH2 domain, R5 and R35, undergo electrostatic interactions with the negatively charged phosphotyrosine, and mutation of either arginine to glutamine resulted in a 40-fold decrease in affinity toward a phosphopeptide probe.^164,232,233^ An R35K mutation maintained the critical positive charge of the residue and was shown to minimally affect probe affinity. Therefore, **ONBK** was inserted at this position and photocontrol of phosphotyrosine recognition was demonstrated through affinity measurement using fluorescence polarization.^164^

**Figure 12:**
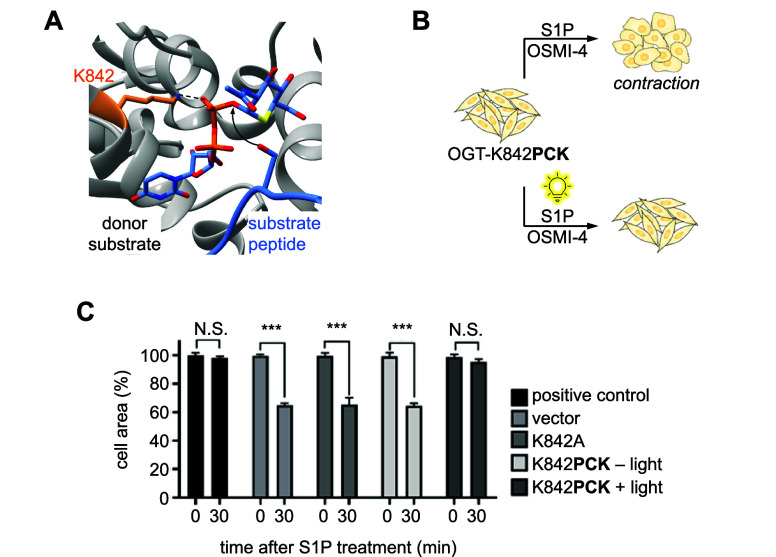
Optical control of cell behavior. (A) K842 undergoes key electrostatic interactions with the phosphate of the donor substrate which are essential for catalysis (PDB 4XIF). (B,C) In the absence of light, treatment of cells expressing OGT-K842**PCK** with S1P and OSMI-4 yields cell contraction. Irradiation and activation of OGT recovers cell shape. Reproduced with permission from ref (159). Copyright 2022 American Chemical Society.

Beyond kinases, other post-translational modification writers have been placed under optical control. For example, **PCK** was introduced into O-GlcNAc transferase (OGT), the writer for the O-linked *N*-acetylglucosamine (O-GlcNAc) protein modification.^159^ K842, a critical lysine involved in stabilizing interactions with phosphates in both the donor cofactor, uridine-diphosphate *N*-acetylglucosamine, as well as in the acceptor substrate, was the target of photocaging (Figure 12A).^234^ After O-GlcNAcylated proteins showed GalNAc incorporation and were conjugated with alkynyl-biotin, a streptavidin pull down and subsequent proteolytic digestion followed by mass spectrometry was used to identify the relative rates of modification of O-GlcNAcylated proteins after OGT photoactivation.^159^ To provide a phenotypic readout of OGT activity, NIH3T3 cells were treated with sphingosine-1-phosphate (S1P), which is known to cause cell contraction in the absence of OGT activity (Figure 12B).^235^ Specifically, O-GlcNAcylation of MYPT1 prevents its phosphorylation, leading to dephosphorylation of myosin light chain and preventing subsequent actin contraction when the signaling pathway is activated by S1P. Caging of OGT activity then results in cellular contraction until irradiation and decaging. To account for endogenous OGT, treatment with a known OGT inhibitor, OSMI-4, suppressed endogenous O-GlcNAcylation.^236^ Thus, treating cells containing photocaged OGT with S1P led to cell contraction, proving the absence of O-GlcNAc modifications.^159^ The observed loss of cell contraction and recovery of a normal phenotype after irradiation with 365 nm light further validated the optical activation of OGT (Figure 12C).^159,235^

**Figure 13:**
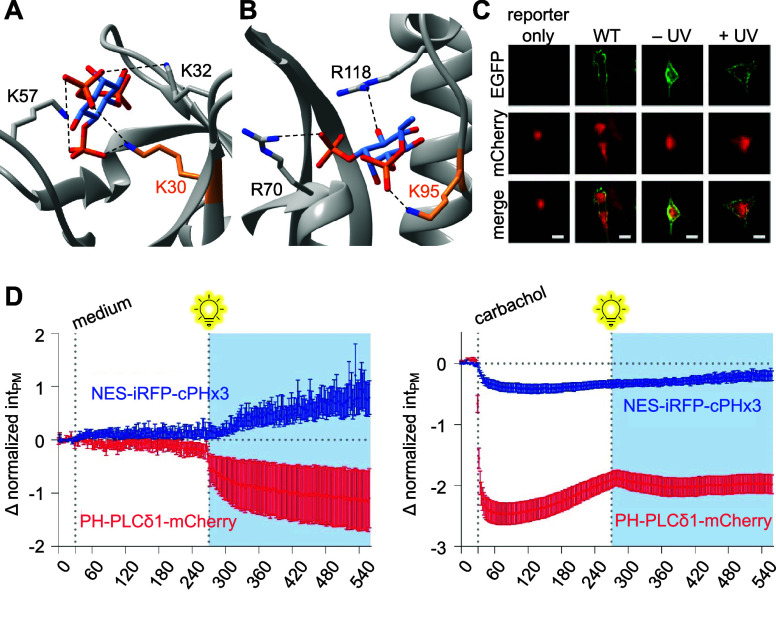
Light-controlled membrane translocation. (A) The crystal structure of the PH domain of PLCδ1 shows the phosphoinositol head of PI(4,5)P_2_ coordinating with K30, K32, and K57 in a series of hydrogen bonding interactions (PDB 1MAI). (B) The crystal structure of SNX3 reveals key interactions of K95, R70, and R118 with PI(3)P (blue; PDB 1OCU). (C) ERK KTR-mCherry translocation to the cytoplasm is triggered only after irradiation and membrane localization of PH-K30**HCK**-EGFP-SOS^cat^. Reproduced with permission from ref (142). Copyright 2021 American Chemical Society. (D) PI(3,4)P_2_ is rapidly generated upon SopB decaging as PI(4,5)P_2_ concentration decreases, as shown by cPHx3 and PH–PLCδ biosensors, respectively. Carbachol-mediated depletion of cellular PI(4,5)P_2_ prevents PI(3,4)P_2_ generation after SopB decaging. Reproduced with permission from ref (141). Copyright 2022 Springer Nature.

Several other cellular processes or protein activities have been put under optical control through the introduction of a photocaged lysine. For example, phosphatidylinositol phosphate (PIP)-binding domains, including the pleckstrin homology (PH) domain of phospholipase C delta 1 (PLCδ1), which recognizes PI(4,5)P_2_, and the phox (PX) domain of sorting nexin 3 (SNX3), which binds to PI(3)P, have been caged with **HCK**.^142,237,238^ PIPs play a role in protein localization to membranous organelles through differential recognition of PIP composition by PIP-binding domains.^239^ The PH domain of PLCδ1 was caged at K30, which undergoes electrostatic interactions with the phosphoinositol head of PI(4,5)P_2_ and forms hydrogen bonds with the 4′- and 5′-phosphates (Figure 13A), while the PX domain of SNX3 was caged at K95, which undergoes hydrogen bonding with the 1′-phosphate of the headgroup of PI(3)P (Figure 13B).^240,241^ Introduction of **HCK** at either of these sites blocked PIP binding through the introduction of steric bulk, removal of the positive charge of the lysine, and disruption of the hydrogen bonding network. Light-activated membrane localization of PH-K30**HCK**-EGFP and SNX3-K95**HCK**-EGFP was demonstrated through fluorescence imaging, and minimal background localization was seen.^142^ A light-triggered plasma membrane translocation approach was applied to SOS2, a guanine nucleotide exchange factor, fused to caged PH, resulting in optical activation of colocalized Ras within the membrane as seen by membrane localization of a fluorescent label and ERK-KTR-mCherry reporter activity (Figures 10B and 13C).^142,217^

LepB is an effector protein with PI(3)P 4-OH kinase activity that converts PI(3)P to PI(3,4)P_2_.^122,242^ During the development of a biosensor for PI(3,4)P_2_, a method was needed to test its affinity.^122^ Thus, LepB was caged with **HCK** at K39, a key active site lysine that directly contacts the α-phosphate of ATP, providing spatiotemporally controlled generation of PI(3,4)P_2_ and corresponding spatiotemporal biosensor recruitment, thereby confirming the function of the biosensor for further PI(3,4)P_2_ studies.^122,242^ Such studies involved investigating the method of PI(3,4)P_2_ recruitment by *Salmonella* outer protein B (SopB) to probe the role of the effector molecule in bacterial invasion of human cells.^141^ SopB was caged with **HCK** at K464 to sterically block its active site and allow for temporal control over enzyme activity and study of PI(3,4)P_2_ response kinetics in the presence or absence of other PIPs. The biosensor showed that PI(3,4)P_2_ is rapidly generated upon SopB activation exclusively in the presence of PI(4,5)P_2_, and that carbachol-mediated depletion of cellular PI(4,5)P_2_ prevents PI(3,4)P_2_ generation after SopB decaging (Figure 13D). These experiments helped reveal the unexpected role of SopB as a phosphotransferase with a sole substrate of PI(4,5)P_2_.

Photocontrol over nucleotide excision repair (NER) has also been established through the incorporation of **HCK** into UvrD, an SF1 helicase.^115^ UvrD is a helicase responsible for unwinding DNA in the 3′ to 5′ direction to separate a damaged DNA strand from an undamaged one.^243^ UvrD was caged with **HCK** at K37, a residue highly conserved across helicases that helps bind the γ-phosphate of ATP.^244^ Successful caging was demonstrated through two fluorescence-based assays, verifying ATPase and helicase activity after light exposure, but not before.^115^ As UvrD is an ATPase, success in implementing photocontrol over this enzyme introduces the possibility to do the same in other ATPases, a broad class of enzymes spanning a multitude of biological roles.^245^

ATP binding was also caged in the transporter associated with antigen processing to study the kinetics of antigen presentation via major histocompatibility complex class I (MHC I).^104^ TAP is part of the peptide loading complex on the ER membrane that funnels antigenic peptides from the cytosol to the ER lumen, where they are eventually loaded onto MHC I and trafficked to the cell surface.^246^ To study the kinetics of this process, the conserved lysine K509 in the ATP-binding site of TAP was caged with **PCK**, preventing interactions between the lysine and the β- and γ-phosphate of ATP.^104,247^ Photocontrol of TAP revealed that MHC I presentation steadily increased after irradiation, following a 1 h lag phase.^104^ Notably, when compared to a shortened coreTAP-K509**PCK** construct, containing only the catalytic domain of TAP, the full TAP-K509**PCK** resulted in greater MHC I surface presentation even before irradiation. This finding illustrated that TAP can act as a scaffold for peptide loading complex formation and promote TAP-independent antigen presentation even when inactive.

In another application to control protein–protein interactions with light, **PCK** was incorporated into the capsid of adeno-associated virus (AAV).^161^ Two arginine residues in AAV are known to play a role in heparan sulfate proteoglycan (HSPG) binding, a key interaction between the virus and host cells crucial to the ability of the virus to infect HEK293T cells. After R to K mutants were shown to retain infectivity, introduction of **PCK** blocked this interaction until irradiation, and decaging reestablished host cell binding.^161,248^ The use of photocaged lysine allows for more precise perturbation of virus-host interactions that may allow for elucidation of further details of the infection process. This discovery is especially useful considering the use of AAVs as prominent gene delivery vectors.^161,248^

**PCK** has also been used to investigate the kinetics of disease-causing mutations.^109^ Isocitrate dehydrogenase (IDH), a citric acid cycle protein that catalyzes the oxidative decarboxylation of isocitrate to α-ketoglutarate, is known to contain an R to K mutation at key active site residue R172 in many cancers.^249^ This mutation allows the enzyme to reduce α-ketoglutarate to (*R*)-2-hydroxyglutarate, a known oncometabolite which competitively inhibits ten-11-translocation enzymes from converting 5-methylcytosine to 5-hydroxymethylcytosine (5-hmC).^250,251^ A decrease in 5-hmC levels is characteristic of many cancers, but the kinetics of decreasing 5-hmC levels after IDH mutation were not well studied.^109,252^ To investigate this process, **PCK** was incorporated at R172 to allow for generation of IDH-R172 K upon irradiation and investigation of the subsequent rates of changing 5-hmC levels.^109^ This investigation revealed that decreasing 5-hmC levels were an early response to IDH mutation, with levels dropping significantly in the first 24 h after irradiation.

**Figure 14:**
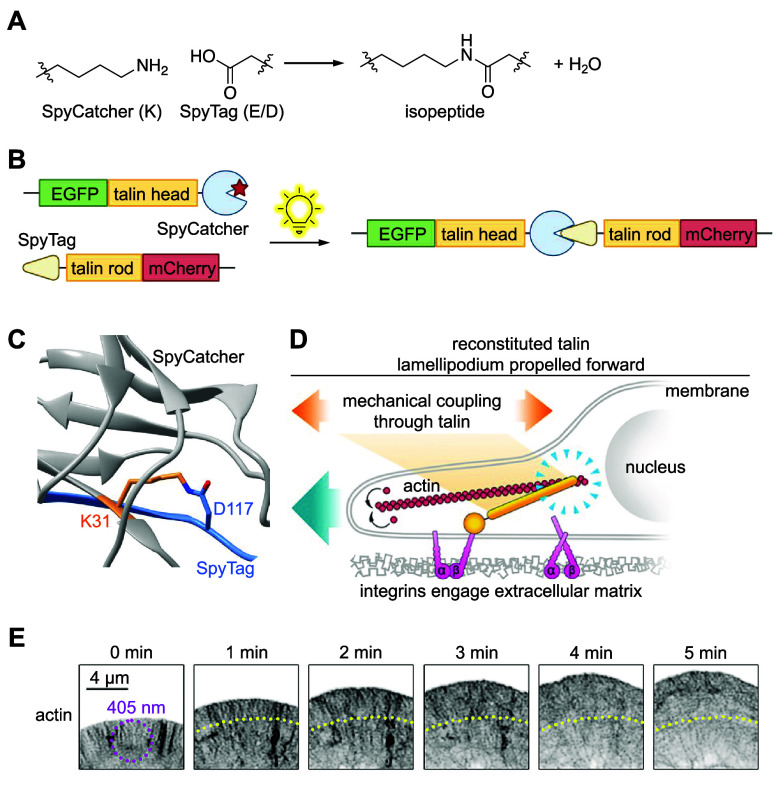
Photocaged lysine used to control SpyCatcher labeling. (A) Mechanism of SpyCatcher ligation to SpyTag.^151^ (B) By splitting talin and fusing each half to either an **HCK**-caged SpyCatcher or SpyTag, reconstitution of the protein was made dependent on irradiation with 405 nm light.^151^ (C) Rendering of K31 of SpyCatcher binding to D117 of SpyTag (PDB 4MLI). (D) Schematic of the role of reconstituted talin in actin-dependent cell spreading.^151^ (E) Lamellipodia extension over 5 min postirradiation of **HCK**-caged SpyCatcher-talin head with SpyTag-talin rod. Panels (D,E) are reproduced with permission from ref (151). Copyright 2023 ACS under CC-BY 4.0 license.

Additionally, the kinetics of the effects of talin on cell mobility were investigated through use of an **HCK**-caged SpyCatcher.^151^ SpyCatcher is a self-labeling protein that forms isopeptide bonds with SpyTag, its peptide partner (Figure 14A).^253^ By attaching one binding partner to either half of a split protein and photocaging the activity of SpyCatcher, reconstitution of the attached protein was placed under optical control (Figure 14B).^151^ Specifically, SpyCatcher was caged with **HCK** at K31, the key residue that covalently reacts with an aspartic acid in SpyTag (Figure 14C).^253^ SpyCatcher has also been optically controlled using photocaged glutamate analogues (see section 8). This tool was used to investigate the activity of talin, the “molecular clutch” that bridges β-integrin to the actin cytoskeleton and coordinates actin-dependent cell spreading upon force sensing (Figure 14D).^254^ The protein was split and caged SpyCatcher was fused to the talin head and EGFP, while SpyTag was fused to the talin rod and mCherry, so that the reaction between the two halves could be tracked via fluorescence microscopy, as well as by anti-EGFP and anti-mCherry Western blots (Figure 14B).^151^ Reconstitution of talin was observed after only 5 s of 405 nm irradiation. This precise temporal control allowed for investigation into the kinetics of lamellipodia extension upon talin reconstitution, where a biphasic extension with an initial fast phase and slower secondary phase was observed (Figure 14E). The kinetics of other cell adhesion processes, such as actin treadmilling and recruitment of other cell adhesion components upon talin activity were also elucidated using this method. The phosphorylation of focal adhesion kinase was revealed among the first activated steps, and recruitment of vasodilator-stimulated phosphoprotein among the last. These studies were uniquely enabled by the precise temporal reconstitution of talin, an approach that might be applicable to other proteins as well.

Finally, a photocaged lysine was used in the development of a tool for the characterization of the ER-specific phosphoproteome.^105^ TurboID is a latest-generation biotin ligase used in proximity labeling.^255^ In this technique, proteins in the vicinity of TurboID within a cellular compartment of interest are labeled with biotin and can then be purified via streptavidin pull-down and identified with mass spectrometry. An additional enrichment of phosphoproteins can be achieved through use of TiO_2_-coated beads.^105^ However, use of TurboID can lead to cellular stress and toxicity due to biotin sequestration and excessive biotinylation of endogenous proteins.^255^ To overcome this limitation, photoTurbo was designed with **PCK** incorporated at K182, a catalytic lysine residue that is necessary for stabilization of ATP during the formation of the reactive biotinyl-5′-AMP intermediate.^105,256^ PhotoTurbo was then selectively activated to limit the amount of background biotinylation that could occur. Using this tool, the phosphoproteome of the ER was characterized, and the effects of cellular stress on ER protein phosphorylation patterns were elucidated.^105^

Overall, photocaged lysines have been used in several applications to introduce optical control over a wide variety of proteins to perturb and study systems ranging from gene expression to cell signaling, protein degradation, and a range of other protein functions in *E. coli*, mammalian cell lines, zebrafish, and *C. elegans*. The prevalence of key lysines that are critical for enzymatic activity makes the residue a great target for caging. Caging removes the basicity of the amino group through the presence of a carbamate, thereby eliminating the positive charge of lysine and thus its ability to undergo electrostatic interactions.^68,70,115,120,165,166^ For example, these unique considerations allow optical control to be established over nearly any NTP-dependent enzyme, as caging key lysines in NTP binding sites prevents interaction with the negatively charged species.^104,115^ Caging also prevents key lysines from undergoing key reactions such as post-translational modifications or isopeptide bond formation.^151^ Caging can also block H-bond formation and overall imposes steric demand in the vicinity of the caging site. Caged lysine derivatives can also at times be used to replace arginine residues, due to their similarity in charge and the lack of a currently developed caged arginine derivative, as long as the K to R mutation is shown to not affect protein activity.^161^ There is significant potential in future applications of photocaged lysines for further study of the behavior of proteins that rely on a key lysine for activity.

## 4 Tyrosine

The first photocaged ncAAs that were introduced into proteins via genetic code expansion were tyrosine derivatives. Tyrosine is an important amino acid to cage due to its prevalence in enzyme active sites and in hot spots of protein–protein interfaces.^257^ It is also a crucial residue in cell signaling, as its phosphorylation state plays a major role in signal transduction.^258^ Native tyrosines can also undergo other post-translational modifications such as nitration and sulfation.^259^ Caging tyrosine not only introduces steric bulk to the residue, but eliminates its ability to H-bond and to act as a nucleophile.^259,260^

**Figure 15:**
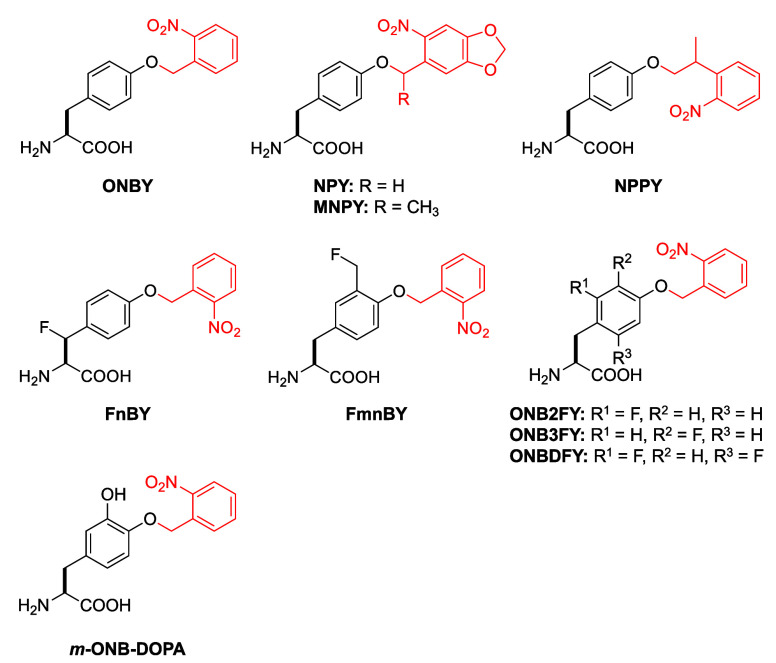
Structures of genetically encoded photocaged tyrosine analogues with caging groups indicated in red.

The first genetically encoded caged tyrosine was *o*-nitrobenzyl tyrosine (**ONBY**, Figure 15).^73^ It has been incorporated into proteins in *E. coli*, mammalian cells, and *C. elegans* with *Mb* and *Mm*PylRS mutants, as well as *Mj*TyrRS mutants (Table 1). However, **ONBY** has a number of limitations. For example, the absorption maximum of the *o*-nitrobenzyl caging group is 254 nm, blue-shifted from the often used 365 nm wavelength for decaging.^26^ Further, the nitro group of **ONBY** is susceptible to reduction to the corresponding amine in certain proteins and bacterial environments, preventing decaging.^131,153−155^ To accommodate this limitation, a strain of *E. coli* with nitroreductases removed has recently been developed.^261^ Despite these potential drawbacks, **ONBY** has been instrumental in introducing optical control over the activity of a broad range of proteins, and is the most prevalent photocaged ncAA of all analogues of any amino acid (Table 2).

A synthetase for the incorporation of **ONBY** was identified via rounds of double-sieve selection of a library of *Mj*TyrRS mutants.^73^ Approximately 10^8^ mutants were generated through the randomization of six residues within the tyrosine binding pocket of the native synthetase (Y32, L65, F108, Q109, D158, and L162). As Y32 and D158 form hydrogen bonds with the hydroxyl group of tyrosine, their mutation was predicted to make native tyrosine binding unfavorable. Selections converged on a synthetase (ONBYRS, Table 1) with five mutations around the binding pocket which did not recognize any endogenous amino acids but selectively incorporated **ONBY** into model proteins in *E. coli*, as confirmed by mass spectrometry. The success of the synthetase was further confirmed by **ONBY** incorporation at Y503 of β-galactosidase, a key residue known to decrease activity 10^4^–10^6^ fold upon mutation.^262^ Caging this tyrosine reduced enzyme activity by ∼95%, as observed using a modified Miller assay.^73,263^ Activity was restored to 67% of wild-type levels after 30 min of irradiation with 365 nm light, although this is an unusually long time to irradiate when compared to later applications of **ONBY**.^73^

Early applications of **ONBY** utilized an *Mj*TyrRS mutant, but this synthetase is limited by its lack of orthogonality in mammalian cells (see section 2). To allow for incorporation of **ONBY** in eukaryotic systems, an *Mb*PylRS was engineered for **ONBY** incorporation through three rounds of alternating positive and negative selection using a library of mutants with L270, Y271, L274, N311 and N313 mutated to all possible amino acids.^74^ The final isolated synthetase contained the mutations L270F, L274M, N311G, and C313G (ONBYRS2, Table 1).

**Figure 16:**
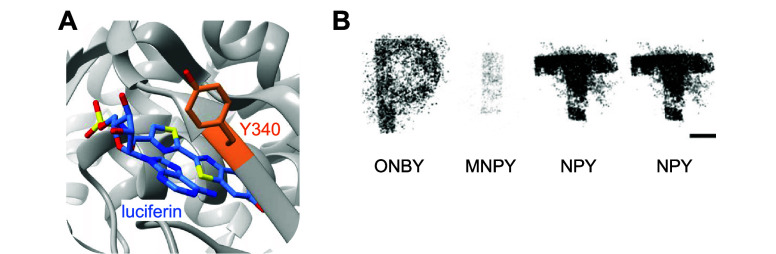
Optically controlled firefly luciferase. (A) Fluc residue Y340 orients the substrate in the binding pocket of the enzyme (PDB: 2D1S). (B) Irradiation of Fluc containing **ONBY**, **MNPY**, or **NPY** in place of Y340 through a mask demonstrates spatial control of Fluc activation in a layer of HEK293T cells. Reproduced with permission from ref (75). Copyright 2017 John Wiley and Sons.

Later analogues of caged tyrosines included **NPY**, **MNPY** and **NPPY**, which carry caging groups with red-shifted absorption spectra, faster decaging kinetics, higher quantum yields, and produce less reactive photolysis byproducts, or feature a combination of these attributes.^75,133^ Incorporation efficiencies of the four photocaged Tyr analogues (**ONBY**, **NPY**, **MNPY**, and **NPPY**) were compared in HEK293T cells using an mCherry-TAG-EGFP reporter together with a derivative of ONBYRS2, ONBYRS3, which contains an extra Y349F mutation for improved efficiency (Table 1).^75,264^ With this reporter, EGFP is only expressed if an ncAA is incorporated; otherwise, translation is terminated at the amber stop codon and only mCherry is expressed. In this comparison, **NPY** and **ONBY** were found to exhibit the greatest incorporation efficiency with this synthetase, while **NPPY** and **MNPY** showed decreased levels of incorporation, as seen by poor EGFP fluorescence. After confirming incorporation of **NPY**, **MNPY**, and **NPPY** through mass spectrometry, the decaging efficiencies of the four tyrosine derivatives were tested using a photocaged firefly luciferase (Fluc) reporter. The key catalytic residue Y340, which is involved in the substrate orientation in the binding pocket (Figure 16A), was mutated to each caged tyrosine analogue.^75,265^ Each ncAA successfully suppressed Fluc activity before irradiation, as luminescence levels were similar to those of a negative control containing no ncAA. Irradiation of the caged Fluc mutants with 365 nm light for 120 s revealed that Y340**NPY** displayed the best recovery of enzymatic activity, while **NPY** and **MNPY** showed faster decaging compared to **ONBY** and **NPPY**. Overall, **NPY** displayed the best compromise between incorporation and decaging efficiencies. Spatial control over activation of Fluc in HEK293T cells was demonstrated though localized irradiation of a cell culture dish (Figure 16B). Optical control over Fluc-Y340**NPY** has also been demonstrated in *Xenopus laevis* embryos.^106^

**Figure 17:**
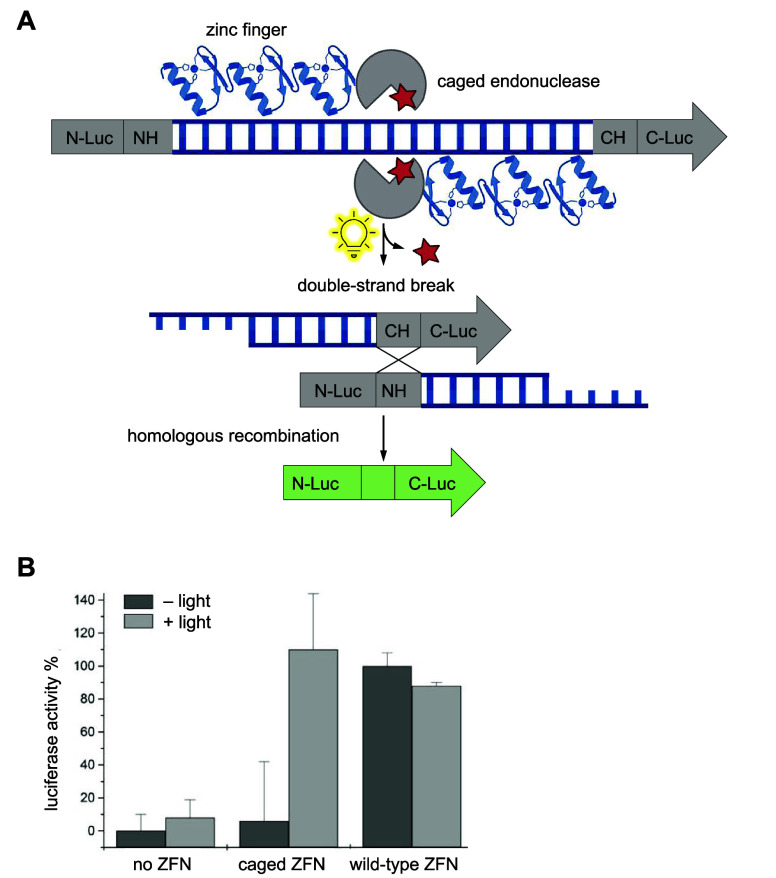
Photocontrol over zinc fingers. (A) ZFN activity resulting in a double-strand break within the reporter gene leads to the generation of an intact luciferase gene, which allows ZFN activity to be read out through luciferase activity.^269^ (B) The **ONBY**-caged ZFN shows no background activity and full restoration to wild-type activity after irradiation. Reproduced with permission from ref (137). Copyright 2011 John Wiley and Sons.

Photocaged tyrosines have been utilized in a wide range of applications in a variety of organisms, including to optically control gene expression through regulation of nuclease and polymerase function, as well as post-transcriptionally and epigenetically through regulation of methyltransferases. Zinc-finger nucleases (ZFNs) are chimeric enzymes consisting of an N-terminal zinc-finger domain and a C-terminal nuclease domain developed for targeted gene editing.^266^ The N-terminal domain recognizes specific DNA sequences through a series of Cys_2_His_2_ fingers that hydrogen bond to the major groove, followed by nuclease-catalyzed nicking of the DNA. Through binding of two ZFNs, a double-strand break (DSB) between the two recognized sites can be achieved.^267^ To introduce an additional level of control over nuclease activity, **ONBY** was introduced into Y471 of the nuclease domain, a residue at the DNA binding cleft of the protein.^137,268^ Optical control of the caged ZFN was demonstrated in HEK293T cells using a reporter gene that produces a functional luciferase enzyme upon gene editing through DSB (Figure 17A).^137,269^ The caged ZFN showed no background activity, while brief irradiation resulted in full restoration of wild-type levels of gene editing, as demonstrated through full restoration of luciferase activity (Figure 17B).^137^ This method could be used with zinc-fingers designed to target any desired DNA sequence for site specific DNA editing with high spatiotemporal control.

**Figure 18:**
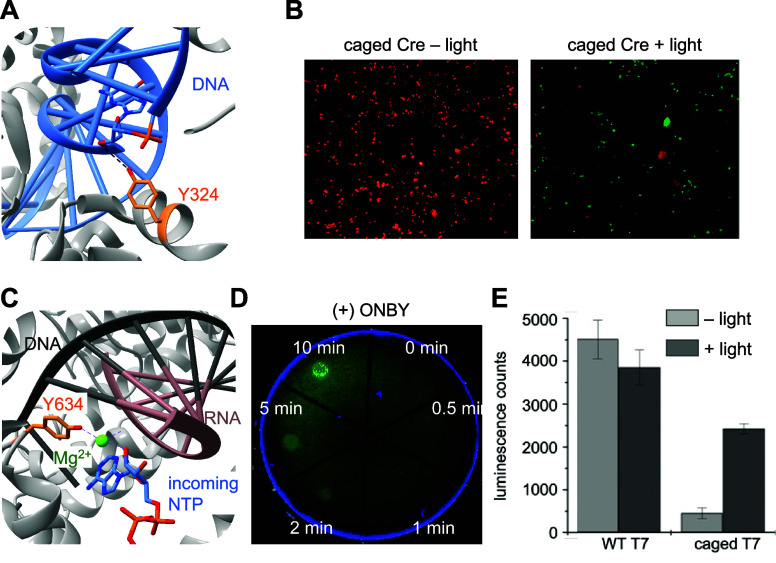
Photocaged tyrosine for optical control of Cre recombinase. (A) Crystal structure of the active site of Cre recombinase. Y324 forms a covalent bond with the phosphodiester backbone of DNA through nucleophilic attack, thereby cleaving the nucleic acid molecule (PDB 1Q3V). (B) Activity of Cre-Y384**ONBY** in HEK293T cells is dependent on light exposure, as seen using a Cre-stoplight reporter. Reproduced with permission from ref (150). Copyright 2009 American Chemical Society. (C) Y634 in T7 RNA polymerase binds a magnesium ion, which in turn coordinates the incoming NTP (PDB 1S0V). (D) Spatial control over expression of EGFP under a T7 promoter upon irradiation of an **ONBY**-caged T7 polymerase in *E. coli*.^144^ (E) Luciferase expression in HEK293T cells transfected with recombinantly expressed, caged T7 polymerase before and after irradiation. Panels (D,E) are reproduced with permission from ref (144). Copyright 2010 John Wiley and Sons.

Another method of optically controlling gene expression using caged tyrosine is the photocaging of Cre recombinase, an important tool in the study of gene function and cell lineage tracing, as discussed in further detail in section 3.^148,150^ The enzyme can lead to the insertion, deletion, or inversion of a DNA sequence between two *loxP* sites (Figure 5A).^180^ To cage Cre recombinase, **ONBY** was encoded at Y324, a key tyrosine residue that catalyzes sequential strand exchange between the two cognate *loxP* sites.^148,150^ Specifically, the phosphodiester backbone of the target DNA undergoes a nucleophilic attack by Y324, which results in the formation of a covalent bond between the 3′-O of guanosine and the hydroxyl group of the tyrosine (Figure 18A).^270,271^ Introduction of a caging group onto this tyrosine blocks nucleophilicity of the hydroxyl group and therefore inactivates the enzyme. Mutants of *Mj*TyrRS, *Mb*PylRS, and *Mm*PylRS have been used to introduce **ONBY** into Cre-Y324TAG in bacterial and mammalian cells.^148,150^ The **ONBY**-caged Cre recombinase was first expressed in *E. coli* cells and delivered into HEK293T cells, and later expressed directly in the mammalian cells; in both cases, its activity was visualized through a Cre-stoplight reporter (See Figure 5A in section 3 and Figure 18B).^148,150,183^ Excellent optical off-to-on switching, reaching wild-type protein levels, and virtually no background activity of the caged enzyme was observed in the absence of light.^148,150^ Other photocontrolled Cre recombinases were generated using caged lysine and histidine (see sections 3 and 7, respectively) and implemented in developing zebrafish embryos for lineage tracing experiments (see section 3).

Optical control of gene expression has also been achieved through genetic code expansion in polymerases. Bacteriophage T7 polymerase is orthogonal to both prokaryotic and eukaryotic systems, allowing for the introduction of an orthogonal expression system into these cells.^272^ Through strategic placement of a caged tyrosine in T7 RNA polymerase, transcription and gene expression of genes under T7 promoter control were photocontrolled (see Figure 3A in section 3). Specifically, **ONBY** was encoded at Y634, a critical residue that coordinates the Mg^2+^ which interacts with the 2’–OH of the incoming NTP and is responsible for driving the synthesized RNA out of the polymerase active site during a conformational change of the enzyme (Figure 18C).^273−275^ Through an *in vitro* transcription assay, the caged enzyme showed virtually no activity, with restoration following ∼2 min of irradiation with 365 nm light.^144^ A maximum of 78% of wild-type activity was achieved after 10 min of irradiation as seen through an *in vitro* transcription/translation assay. Expression of EGFP under a T7 promoter was then shown to be dependent upon irradiation of engineered *E. coli* cells, as demonstrated by spatially restricted irradiation, which only yielded fluorescence in irradiated areas of a plate (Figure 18D). A firefly luciferase gene was also placed under a T7 promoter in HEK293T cells, and Fluc expression was shown to increase 5-fold after irradiation when cells were transfected with caged T7 polymerase (Figure 18E). This supports the general applicability of an **ONBY**-caged T7 RNA polymerase to introduce photocontrol over the expression of any gene of interest placed under a T7 promoter in bacterial or mammalian expression systems. See section 3 for discussion of a lysine-caged T7 RNA polymerase in which the entire expression system, including the caged polymerase, genetic code expansion machinery, and protein of interest under a T7 promoter was genetically encoded in mammalian cells.^143^ Additionally, a DNA polymerase, Taq, was caged with **ONBY**, as discussed later in this section.^145^

Another method of optically controlling gene expression is the development of a light-activated methyltransferase.^158^ In eukaryotes, mRNA is processed through 5′ capping, splicing, and polyadenylation.^276^ Methylation of the mRNA cap is necessary for efficient recognition by cap-binding proteins such as eukaryotic translation initiation factor (eIF4E).^276^ By regulating activity of a methyltransferase, gene expression can be controlled, albeit on a global, not gene-specific, level.

To date, two methyltransferases have been caged through the introduction of **ONBY**.^158,160^ Guanine N7MTase Ecm1 was caged in *E. coli* with **ONBY** at Y284, a highly conserved residue in the enzyme active site that coordinates the cap guanosine through H-bonds with the 2’–OH and N3 of the cap.^158^ A biochemical assay revealed that irradiated enzyme showed 97% conversion of a 5′ cap analogue after an hour, while nonirradiated enzyme only saw about 5% conversion, confirming the success of the caging group in modulating enzyme activity. Protein/histone arginine methyltransferase 1 (PRMT1) has also been caged with **ONBY**.^160^ PRMTs transfer a methyl group from S-adenosyl-l-methionine to histones, determining which sections of the genome are transcriptionally active.^277^ PRMTs also play a role in disease pathogenesis, as incorrect methylation of histones adversely affects gene expression, mitosis, and DNA repair, which correlate with cancer.^277^ PRMT1 is the most prevalent protein in the PRMT family and plays a critical role in neuronal development.^278^ The protein was caged at Y291, a site chosen based on natural enzyme regulation, as phosphorylation at this site restricts activity by blocking substrate binding.^160^ Introduction of the caging group decreased methyltransferase activity by 7-fold and irradiation with 365 nm light for as little as 2 min restored wild-type levels of enzymatic activity as measured by biochemical assays. Methyltransferases were also optically controlled using photocaged cysteine (see section 5).

**Figure 19:**
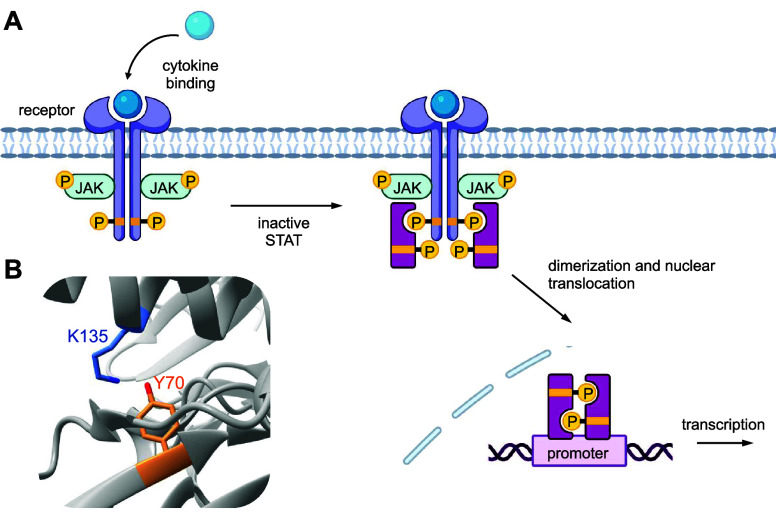
Photocaged tyrosine for the optical control of cell signaling. (A) The JAK/STAT signaling pathway.^280^ (B) Y70 of the IL-20 receptor is located at the binding interface of the receptor with IL-24. The hydroxyl group of Y70 forms a hydrogen bond with K135 of IL-24 (PDB 6DF3).

As discussed in detail in sections 3 and 8, photocaged ncAAs are often used to perturb cell signaling pathways. Tyrosine phosphorylation serves as a means of signal transduction dependent on its phosphorylation state.^258^ Caging critical tyrosines prevents phosphorylation and allows investigation into the roles and kinetics of individual steps in signaling pathways.^74,279^ For example, Y701 of signal transducer and activator of transcription 1 (STAT1), which is phosphorylated by Janus kinases (JAK) in the JAK/STAT signaling pathway, has been caged with **ONBY**.^74^ The JAK/STAT pathway is activated through cytokine binding to cell surface receptors and ultimately results in the transcription of target genes (Figure 19A).^280^ To demonstrate the utility of **ONBY** for the optical control of cell signaling, STAT1-Y701**ONBY** was expressed in HEK293 cells.^74^ To activate the JAK/STAT pathway, cells were treated with interferon, and phosphorylation was detected by Western blot. In the absence of light, phosphorylation of endogenous STAT1 but not of STAT1-Y701**ONBY**-EGFP was observed, and only when treated with interferon and light was photocaged STAT1 phosphorylated. No background phosphorylation in the absence of light was observed. These results represented the first time that **ONBY** had been genetically encoded in mammalian cells and showed the potential for optical control of sites modified by PTMs.

The JAK/STAT pathway was further investigated through the photocaging of human interleukin 24 (IL-24) receptors with **ONBY**. The binding of IL-24, a cytokine which binds to membrane receptors such as the IL-20 receptor (IL-20R1/IL-20R2) (Figure 19A),^281^ was photocontrolled using IL-20R-**ONBY** and demonstrated through microscale thermophoresis. IL-20R2D, an IL-20R2 derivative optimized for *E. coli* expression, was caged at Y70 and showed a 300-fold decrease in binding affinity.^146^ Site Y70 is located on the binding interface between IL-24 and its receptor, and its hydroxyl group forms a hydrogen bond with K135 in IL-24 (Figure 19B).^146,282^ Subsequent irradiation restored levels of binding to near wild-type activity. Caging at this site therefore disrupts key interactions between IL-20R and its substrate, preventing binding until irradiated and creating an additional tool for future studies of the JAK/STAT pathway.^146^

Another study into the optical control of cell signaling utilized **ONBY** for light-activation of protein phosphatase-1 (PP1)-disrupting peptide (PDP) in the mitogen-activated protein kinase (MAPK) pathway.^138^ PDP-*Nal*, an optimized, cell permeable form of PDP, modulates PP1 activity and prevents phosphorylation of PP1 substrates.^283,284^ As no tyrosine residues were available to cage along the interface between PP1 and PDP-*Nal*, the phenylalanine in the RVTF binding motif was replaced with **ONBY**, as mutation to tyrosine showed minimal effect on PDP-*Nal* binding.^138^ The steric bulk of **ONBY** prevented most PP1/PDP-*Nal* interaction until irradiated, as evidenced by in-cell western for phosphorylated histone H3, a substrate for PP1. Development of this caged PDP-*Nal* allows for future studies into the effects of selectively activating PP1 at different stages of mitosis.

Phosphatases have also been caged with photocaged tyrosines.^140^ As discussed in section 3, Src-homology 2 domain-containing protein tyrosine phosphatase 2 (SHP2) is a key phosphatase in the ERK/MAPK signaling pathway, although its mechanism of action is not entirely understood.^229,285^ To create a tool for further investigation of its function, SHP2 was caged with **NPY** at Y279, a residue in the key KNRY motif of SHP2 that is responsible for phosphotyrosine recognition and stabilization through stacking interactions with the aromatic ring of the substrate.^140,286,287^ Disrupting the H-bonding interactions between Y279 and the other members of the KNRY motif destabilizes the region and prevents substrate binding. Caging this residue sterically blocks the substrate from interacting with the active site of SHP2 until irradiated.^140^ Optical control over SHP2 activity was demonstrated using the fluorogenic phosphatase substrate DiFUMP in a biochemical assay, and the effect of decaging on ERK signaling was assessed using an ERK KTR-mCherry reporter in human cells (see Figure 10B in section 3). Nonirradiated SHP2-Y279**NPY** was shown to be present in the nucleus with a similar N/C ratio to the negative, reporter-only control. After irradiation, cytoplasmic localization of the reporter occurred rapidly and plateaued after about 40 min. The mechanism of the effects of SHP2 on ERK/MAPK signaling were then further studied using a photocaged lysine, as discussed in section 3.

Antibodies have also been targets for photocaged tyrosine incorporation to optically control antigen binding.^128−133,288,289^ In particular, nanobodies, which are single-domain antibody fragments composed of the variable domains of the heavy-chain-only antibodies of camelids, have been expressed with **ONBY**, **NPY**, and **MNPY** to introduce a method of temporal control over antibody binding.^128−133,288−290^ Control of antibody binding after translation has potential to achieve spatiotemporal activation over common uses of nanobodies such as the detection, masking, or inhibition of target antigens, as well as investigation into antigen function through nanobodies used with targeted proteasomal degradation systems.^128,131,291−293^

Tyrosine is commonly found along the paratope-epitope interface of nanobodies, making it a good target for photocaging.^130,294^ For example, the nanobody 7D12, which targets the epidermal growth factor receptor (EGFR), was caged through the incorporation of **ONBY** in *E. coli*.^128^ Using human epidermal cells with high expression levels of EGFR (A431 cells), whole-cell ELISA and on-cell western-type assays demonstrated no binding when 7D12 was photocaged with **ONBY** at Y32 or Y113, even when high concentrations of nanobody were used.^128^ After irradiation with 365 nm light, binding was restored to near wild-type levels. Notably, however, 7D12-Y109**ONBY** still recognized EGFR. As Y32, Y113, and Y109 were all located at the binding interface of the antigen, each was predicted to be a suitable target for caging. Molecular dynamics simulations revealed more movement, or breakage of key interactions, from the starting conformation in 7D12-Y31**ONBY** and 7D12-Y113**ONBY** compared to Y109**ONBY**.^128^ Residue Y109 was also revealed to be positioned largely parallel to the antigen surface, leading to limited antigen interactions. These results revealed that although the plasticity of protein–protein interactions can sometimes accommodate significant structural modifications, such as caging groups, optical control can typically be achieved through caging of alternate residues. Other photocaged nanobodies were generated following the same strategy, including those targeting sfGFP and HER2.^130^

**Figure 20:**
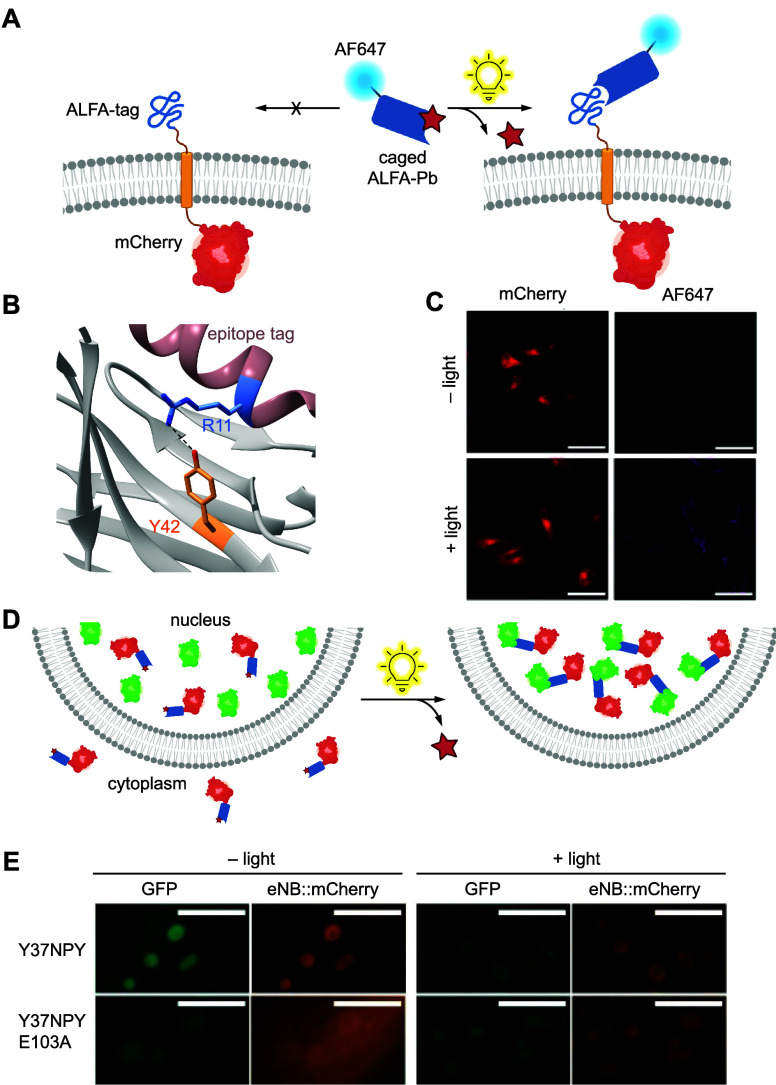
Optical control of nanobodies. (A) Activity of caged ALFA-Pb in HeLa cells was visualized through expression of a fluorescent AF647-tagged ALFA–Pb-Y42**ONBY** and cell surface-localized mCherry-ALFA-tag. Presence of **ONBY** prevents ALFA-Pb binding to the epitope tag until irradiation and decaging.^131^ (B) Y42 (orange) of an anti-ALFA-tag antibody undergoes hydrogen bonding with R11 (blue) of the epitope tag (pink; PDB 6I2G). (C) AF647-tagged ALFA–Pb-Y42**ONBY** (blue) does not localize to surface mCherry-ALFA-tag (red) until irradiated (purple). Reproduced with permission from ref (131). Copyright 2022 John Wiley and Sons under Creative Commons CC BY license. (D) To visualize and quantify anti-GFP photobody binding before and after irradiation, EGFP with an NLS was coexpressed with the photocaged nanobody fused to mCherry. The nuclear to cytoplasmic ratio (N/C) of mCherry fluorescence was then used to quantify the level of nanobody binding before and after irradiation.^133^ (E) The above experiment performed in *C. elegans.* Reproduced with permission from ref (133). Copyright 2022 John Wiley and Sons under Creative Commons CC BY license.

Another category of photocaged nanobodies are those that target short peptide epitope tags.^131^ These antibodies allow for recognition of any tagged protein and are commonly used in methods such as immunoblotting, immunofluorescence, and protein purification.^295^ Their photocaging allows for optical control over methods such as fluorescent labeling of any tagged protein. Specifically, a photocaged antibody for ALFA-tag (ALFA-Pb, Figure 20A), an ultrahigh affinity nanobody for a tag absent in the human proteome, was generated by incorporating **ONBY** in place of Y42, a residue located in the binding site that undergoes hydrogen bonding with R11 of the tag (Figure 20B).^131^ Optical control was demonstrated by two experiments: 1) treating *E. coli* displaying ALFA–Pb-Y42**ONBY** with ALFA-tagged sfGFP, as measured by flow cytometry, and 2) imaging of HeLa cells expressing a fluorescently tagged ALFA–Pb-Y42**ONBY** and cell surface-localized mCherry-ALFA-tag (Figure 20A). Both studies showed no binding of the photocaged nanobody and restoration of full binding after irradiation with 365 nm light for 20–45 s (Figure 20C).

Intriguingly, the aim of creating an **ONBY**-caged GFP nanobody required GCE troubleshooting.^132^ When mutating the codon for Y37 in the nanobody to an amber stop codon, read-though of the stop codon was observed, as seen by high levels of protein expression and antibody binding in HeLa cells even in the absence of the synthetase or **ONBY**. It has been shown that translation accuracy toward the 5′ region of mRNA is notably reduced compared to later translation.^296−299^ To address the observed readthrough, an EGFP gene was appended upstream of the nanobody, followed by an F2A ribosomal skipping site.^132^ This allowed for both EGFP and the nanobody to be translated in a single ribosomal passage, while moving the amber stop codon away from the lower fidelity 5′ region.^300^ Successful incorporation of **ONBY** and **NPY** with minimal background incorporation was then possible, providing the first example of photocontrol over nanobodies expressed in mammalian cells.^132^

A recent attempt to introduce photocaged nanobodies into *C. elegans* exposed an interesting reliance on the cellular environment for successful photocaging.^133^ As in the example above, an anti-GFP nanobody was photocaged at Y37 with both **ONBY** and **NPY** and expressed in *C. elegans* using an *Mm*PylRS mutant with the same mutations as ONBYRS3; in contrast to other approaches, this anti-GFP nanobody construct did not feature a ribosomal skipping site.^132,133^ While identical photocaged nanobodies did not show antigen binding in HeLa cells until irradiated, when expressed in *C. elegans*, the caged nanobodies were able to bind GFP even before irradiation.^133^ However, no clear conclusion could be drawn as to why this caging was successful when the caged nanobody was expressed in HeLa cells, but failed in *C. elegans*. To address this, computational alanine scanning was used to determine which residues could be mutated to decrease the caged-nanobody binding affinity, while still retaining enough affinity to bind the antigen after decaging.^133^ EGFP with a nuclear localization sequence was coexpressed with the photocaged nanobody fused to mCherry, and the nuclear to cytoplasm ratio (N/C) was used to measure the level of nanobody binding before and after light irradiation (Figure 20D). Upon the addition of an E103A mutation, background binding prior to light decaging was abolished, as measured by a low N/C ratio (Figure 20E). Residue E103 lies along the nanobody/GFP interface, and engages in stabilizing interactions with EGFP, as seen by its high negative binding free energy.^301^ After irradiation, decaged nanobody localized to the nucleus, although the **ONBY**-containing mutant required a significantly longer irradiation time (640 s) than the **NPY**-containing mutant (160 s) to reach the same N/C ratio of ∼2.3.

Controlling protein–protein interactions with light was also applied to tobacco etch virus (TEV) protease through introduction of **NPY** at Y178, a key residue involved in hydrogen bonding with peptide substrates.^75,302^ Activity was measured through a GloSensor reporter, which uses a circularly permuted luciferase wherein the N- and C-termini are linked by a protease cleavage site. This reporter increases in luminescence once cleaved by TEV protease, as the conformational constraints on the protein are relieved.^101^ Caged TEV protease produced negligible GloSensor activity, similar to that of a minus ncAA control, while irradiation restored protease activity.^75^ Activity of a wild-type positive control was not investigated, however, so the percentage of activity restoration upon irradiation was not quantified. TEV protease has also been rendered light-responsive through cysteine caging (see section 5), and its optical control could be used for a variety of molecular biology applications such as light-dependent protein cleavage or affinity tag removal.^303,304^

A final example of protein–protein interactions being placed under optical control through the use of a photocaged tyrosine is the caging of the innate immune receptor TLR8.^147^ TLR8 is a member of the toll-like receptor (TLR) family, which triggers innate and adaptive immune responses following recognition of pathogen-associated and danger-associated molecular patterns.^305^ Using **ONBY**, each tyrosine residue in TLR8, 34 in total, was probed for its impact on activity using a secreted alkaline phosphatase (SEAP) reporter gene.^147^ The caging group installations were expected to either 1) sterically block ligands from entering the two pockets of the receptor, 2) prevent proper interaction between two TLR8 protomers, or 3) prevent TLR8 cytosolic domains from properly interacting with downstream adaptors. TLR8 function was blocked until irradiation yielded the decaged tyrosine for 11 of the tested sites.^147^ Importantly, this screen discovered residues at the interface dimer that could completely prevent TLR8 activity through caging, a finding that had not been previously discovered through conventional mutagenesis methods such as alanine scanning.

**Figure 21:**
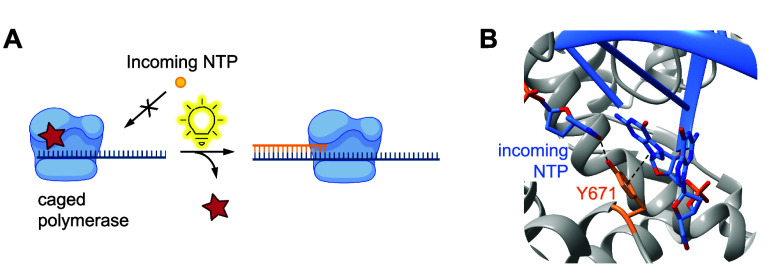
Photocaged tyrosine for optical control of Taq polymerase. (A) Activity of Taq polymerase is blocked until irradiation and decaging of **ONBY** occurs.^145^ (B) Y671 of Taq polymerase is responsible for positioning the primer-template pair in the active site, as well as aligning the incoming dNTP with the first base of the template (PDB 2KTQ).

In addition to light-regulation of protein–protein interactions as discussed above, **ONBY** was also applied to achieve optical control of protein-nucleic acid interactions, specifically those of Taq DNA polymerase (Figure 21A).^145^ Y671 is a residue crucial for positioning the primer-template pair in the active site and aligning the first base of the template with the incoming dNTP after the enzyme undergoes an open-closed conformational change (Figure 21B).^145,306^ Photocaging at Y671 completely eliminated Taq function, while a 5 min irradiation of the recombinantly expressed polymerase was able to restore 71% activity, as seen by comparison of the PCR products produced by caged, irradiated, and wild-type enzymes.^145^ This photocaged enzyme proved useful for hot-start PCR, a method where the polymerase is inactivated until the PCR temperature is high enough to prevent nonspecific primer binding and the synthesis of the resulting nonspecific DNA amplification products.^307,308^ As this critical tyrosine residue is conserved in many other DNA and RNA polymerases, caging with **ONBY** is likely transferable to many other polymerases.^309^ Bacteriophage T7 RNA polymerase was similarly caged with **ONBY**, as discussed earlier in this section.^144^

**Figure 22:**
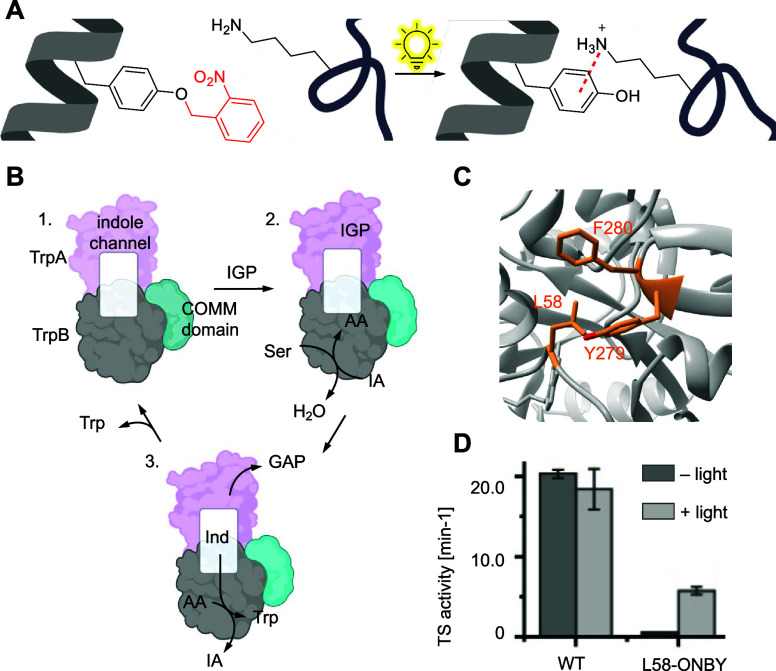
Photoactivation of GGGPS and tryptophan synthetase. (A) Irradiation of GGGPS-W139**ONBY** relieves the steric clashing caused by the introduction of the caging group, which allows the resulting tyrosine to undergo a cation-π interaction with a neighboring lysine.^153^ (B) Mechanism of tryptophan synthetase.^155^ (C) L58 of tryptophan synthetase sits in a pinwheel position with Y279 and F280 (PDB 1A5S). (D) Caged tryptophan synthetase activity before and after irradiation compared to wild-type enzyme activity. Reproduced with permission from ref (155). Copyright 2019 retained by the authors under Creative Commons CC BY 4.0 license.

Another method of controlling enzymatic function is to cage the interface of a protein that requires oligomerization for full catalytic activity. This was demonstrated with geranylgeranylglyceryl phosphate synthase (GGGPS), a hexameric enzyme that has previously shown a 380-fold decrease in catalytic activity when mutations were introduced at the subunit interfaces, limiting the protein to dimerization.^153,310^ GGGPS features three interfaces involved in the formation of its hexameric complex: 1) the dimer module interface, 2) the interconnecting interface, and 3) the ring interface.^310^ A key association at the ring interface is a cation-π interaction between an aromatic tryptophan (W139) of one monomer and a cationic lysine of another. Since a W139Y mutant was shown to still form hexamers, a W139**ONBY** mutation was used to cage the enyzme.^153^ Steric clashing introduced by **ONBY** at this position prevented the residues from orienting correctly for the cation-π interaction to occur (Figure 22A). Nonirradiated GGGPS-W139**ONBY** expressed in *E. coli* was then shown to exclusively form dimers and be enzymatically inactive, as shown through size exclusion chromatography and a colorimetric assay measuring a pyrophosphate byproduct, respectively. After 3 min of 365 nm irradiation, approximately 30% of the protein formed hexamers, restoring about 27% of wild-type activity. Incomplete activation was due to the reduction of **ONBY** in cells as seen by protein sequencing, which caused the ncAA to become unresponsive to irradiation.

In addition to the often-used approach of blocking the active site of proteins with a caging group, blocking an allosteric site has also been explored, as discussed extensively in section 9. Briefly, the introduction of a photocaged tyrosine into loop 1 of the imidazole glycerol phosphate synthase (ImGPS) provided allosteric photocontrol of this bienzyme complex.^154^ Initial expression of ImGPS-Y39**ONBY** in *E. coli* saw successful blocking of ImGPS activity, but only ∼20% of wild-type activity was restored after irradiation. Mass spectrometry revealed that 20–60% of the incorporated *o*-nitrobenzyl was reduced to the corresponding aniline, preventing full decaging and recovery of activity. When **ONBY** was replaced with **NPY** at residue Y39, activity matched the negative control prior to activation, with near wild-type levels of activity (6-fold increase) restored following irradiation.^154^ Mass spectrometry revealed that <10% of **NPY** at this site was reduced compared to the 20–60% of the **ONBY**-containing mutant, suggesting **NPY** incorporation is a possible means of overcoming nitro-stability and subsequent decaging issues linked to **ONBY**.

Another protein complex that has been photocontrolled via **ONBY** incorporation at a site that affects allosteric regulation is tryptophan synthetase (TS).^155^ TS is a heterotetrameric complex consisting of two monomeric TrpA subunits that enclose a homodimeric TrpB subunit, and is highly allosterically regulated.^311,312^ Indole-3-glycerol phosphate (IGP) binding to TrpA lowers the K_m_ for serine binding in TrpB leading to subsequent aminoacrylate (AA) formation in TrpB from the condensation of serine and internal aldimine (IA), increasing TrpA catalytic activity toward IGP (Figure 22B).^311,313,314^ Structural changes in a communication (COMM) domain between the subunits allows the transfer of this allosteric communication to occur.^315^ A residue located near the COMM domain, L58 in TrpA, was mutated to **ONBY** after an L58Y mutation demonstrated no effect on TrpA activity.^155^ The effect of the tyrosine mutation was probed because decaging of **ONBY** leaves behind a tyrosine scar at this residue. In this position, **ONBY** sits in the intramolecular indole channel in the protein complex through which indole is passed from TrpA to TrpB, where it orients in a pinwheel arrangement with Y279 and F280 in TrpB, two residues that serve as gating residues for the channel (Figure 22C).^316,317^ These π–π interactions likely have a significant effect on allosteric communication within the protein complex.^318^ It was found, however, that around 50% of **ONBY** incorporated at this site was reduced and could not be decaged, preventing full restoration of enzyme activity (Figure 22D).^155^ Further experiments investigating the activities of TrpA and TrpB individually supported the conclusion that **ONBY** inhibited allosteric communication between the subunits in three primary ways: 1) by limiting activation of TrpA upon binding with TrpB, 2) by suppressing aminoacrylate formation upon IGP binding, and 3) by limiting the activation of TrpA activity caused by serine binding. Indole movement through the intermolecular channel between TrpA and TrpB also appeared to be blocked by the caging group. Overall, these results show the potential of suppressing allosteric regulation in a protein complex by caging a key site involved in allosteric communication.

Inteins have also been the target of photocaging with **ONBY**.^116,117^ Inteins are protein domains that autocatalytically excise themselves from a precursor protein, allowing the remaining exteins to ligate in a process called protein splicing, as further discussed in section 5.^319^ Gp41–1, a fast *trans*-splicing intein, was the target of this caging approach.^116,320,321^ The caged protein had to be produced in the periplasm of *E. coli*, as cytoplasmic expression led to significant amounts of reduced, nonphotoactive **ONBY**.^116^ F65 is a highly conserved and structurally critical residue that stabilizes the key histidine near the splicing site that activates the thiol to initiate the N–S acyl shift that begins intein splicing (see Figure 27A in section 5).^322,323^ This stabilization occurs through a cation-π interaction, and mutation of this histidine to another residue, or F65 mutation to a nonaromatic amino acid, have both been shown to abolish intein splicing activity.^322,323^ Irradiation of Gp41–1-F65**ONBY** yielded close to wild-type levels of splicing activity, while no activity was observed in the absence of light, as shown by SDS-PAGE.^116^ In another application of photocaged inteins, peptide circularization was optically controlled.^117^ An intein has also been placed under optical control using a photocaged cysteine, which enabled optical control of a variety of proteins in mammalian cells (see section 5).

**Figure 23:**
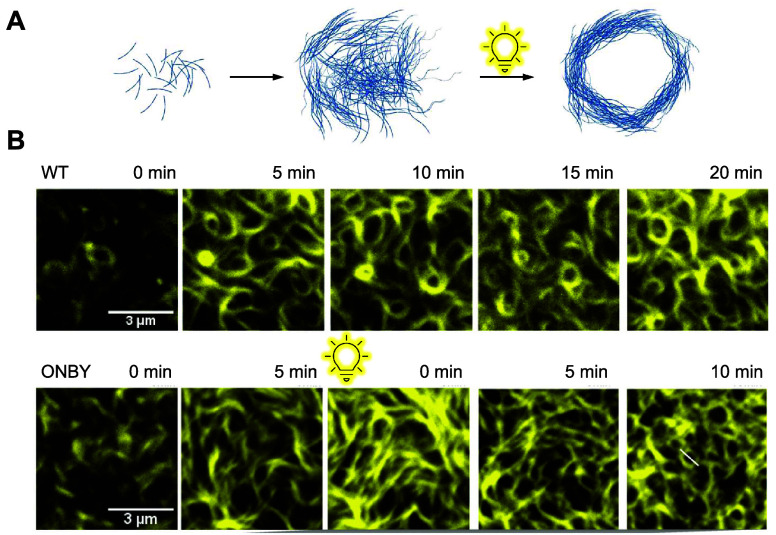
Photocontrol of filamentation. (A) Caged FtsZ is unable to self-assemble into its characteristic circular structures until irradiated and decaged.^163^ (B) As shown through TIRF imaging, while wild-type FtsZ self-assembles into circular structures, FtsZ-Y222**ONBY** only forms a chaotic mesh. After irradiation, the ability to form circular structures is restored. Reproduced with permission from ref (163). Copyright 2021 John Wiley and Sons under Creative Commons CC BY license.

Photocaging has also been used in bottom-up designs to study the mechanics of key biological systems. Filamenting temperature-sensitive mutant Z (FtsZ) is a highly conserved prokaryotic division protein and is a key target for efforts toward building a synthetic minimal cell.^324,325^ FtsZ was caged with **ONBY** at Y222, a tyrosine in the interdomain region between the N- and C-terminal domains of FtsZ shown to be critical for filament formation through its role in promoting the hinge-like movements of these domains that allow the protein to shift between its straight and curved forms.^163,326−328^ Mutation of this residue to a bulkier tryptophan has been shown to sterically constrain this movement, as well as decrease the enzyme’s GTPase activity.^327,328^ Caging FtsZ-Y222 with **ONBY** resulted in an over 95% decrease in GTPase activity of the enzyme compared to wild-type, while a 5 min irradiation increased activity of the mutant over 22-fold, as measured by released inorganic phosphate in a GTPase activity assay.^163^ The ability of the mutant to self-organize was then analyzed using total internal reflection fluorescence (TIRF) microscopy. TIRF imaging revealed that FtsZ-Y222**ONBY** was unable to form the circular structures characteristic of wild-type protein, instead forming a chaotic mesh (Figure 23A-B). After irradiation, the protein could again self-assemble into its characteristic circular structures (Figure 23B). Interestingly, although the Y222**ONBY** mutant showed successful control over activity, only 38% of FtsZ-Y339**ONBY**, another tested mutant, was decaged. ESI-MS confirmed approximately 48% of Y339**ONBY** had been reduced and was therefore inactive toward photolysis. The difference in stability observed between Y222**ONBY** and Y339**ONBY** further highlights the effects of local protein microenvironment on the suitability of the *o*-nitrobenzyl caging group. Overall, this work allows for tunable control over FtsZ filamentation and paves the way for conditional control over other systems used in creating a minimal synthetic cell.

Another unique use of photocaged tyrosines was the caging of a critical residue in a toxic protein to allow overexpression and purification in *E. coli*.^156^ YoeB_Sa1_ is a toxin produced by *Staphylococcus aureus*, and its full mechanism of toxicity has not been studied due to its inherent toxicity and inability to be overexpressed for purification.^156,329,330^**ONBY** was used to cage a tyrosine critical for protein toxicity, Y88. Mutation to **ONBY** allowed YoeB_Sa1_ to be expressed in an inert state before toxicity was restored for later experiments.^156^ This new method for isolating proteins facilitated the discovery that YoeB_Sa1_ possesses only ribosome-dependent, not independent, RNase activity, despite previous predictions based on the similarity of YoeB_Sa1_ to other toxins featuring ribosome-independent RNase activity.^156,331,332^

As exemplified by the numerous applications discussed in this review, optical control over protein function has frequently been achieved through rational selection of a caging site, based on structural and/or mechanistic information on the protein of interest. In cases where a site is not obvious, a computational approach for the identification of “cageable” residues, initially developed using **ONBY**, could provide a solution.^107,333^ The approach first computationally generates a list of potential anchor sites, or residues near the functional site of the protein, that are likely to block activity when caged and restore activity when decaged to tyrosine. Tyrosine was chosen as the anchor residue, or residue that is left behind at the anchor site after decaging, due to its prevalence in active sites. This is an important consideration as the ideal anchor residue would be identical to the original anchor residue, or at least have minimal effect on protein activity. Rosetta can then be used to determine which of these sites meets a series of geometric and energetic parameters. The sites generated from these computations are then subjected to experimental validation to assess the ability of **ONBY** to introduce optical control over protein activity. This method was used to generate a variety of caged proteins, including a kinase, a GTPase, a caspase, an RNA demethylase, and a luciferase.^107^

Photoactivatable fluorescent probes have also been generated using caged tyrosines. The chromophore of sfGFP, for example, was caged with **ONBY** at the central residue Y66.^113,114^ When caged with **ONBY**, no fluorescence was observed despite confirmation of chromophore maturation.^114^ The lack of fluorescence was likely caused by the steric bulk of the *o*-nitrobenzyl group interrupting the proton-wire and quenching the singlet excited state of the chromophore.^113,114,334^ Fluorescence was re-established upon irradiation with 365 nm light.^113^ The crystal structure of sfGFP-Y66**ONBY** revealed a unique domain-swapped dimeric structure, where domain 1 of one molecule associates with domain 2 of another molecule due to backbone rearrangement caused by the steric interference of **ONBY**.^114^ sfGFP-Y66**ONBY** is therefore notably less stable and more prone to aggregation than wild-type sfGFP. This residue has also been caged with fluorotyrosine derivatives, as discussed below.^78^

In addition to the many uses of **ONBY** and **NPY** for optical control, other analogues of tyrosine have been caged for unique applications. One example is a class of photocaged tyrosines that utilizes fluorinated analogues of the amino acid. Fluorine is useful as a biological probe due to its high electronegativity and small size, yielding minimal structural impact. Adding fluorines also allows for tuning of the p*K*_a_ of the side chain; while the p*K*_a_ of the phenolic proton of tyrosine is ∼10, the pKas of fluorotyrosines can be as low as 5.^335,336^ Photocaging these near-natural ncAAs for incorporation masks them from endogenous cellular machinery, preventing global incorporation into proteins.^78^ Photocaged fluorotyrosines employed for optical control of proteins include **ONB2FY**, **ONB3FY**, and **ONBDFY** (Figure 15).^78^ Two *Mj*TyrRS were evolved for these ncAAs through a series of chloramphenicol acetyl transferase-based selections.^78^ The first synthetase identified, ONB2FYRS (Table 1), was able to incorporate **ONB2FY** and **ONB3FY**, while the second synthetase, ONBDFYRS (Table 1), could incorporate all three fluorine-containing ncAAs. The successful incorporation and decaging of these ncAAs was confirmed by expression into sfGFP at the chromophore tyrosine, Y66. After decaging with 365 nm, **ONB2FY** and **ONBDFY**, which contain fluorines at the *ortho* position, were shown to blue-shift the λ_em_ from 513 nm (wild-type) to 503 and 509 nm, respectively. However, **ONB3FY** caused a slight red-shift of the chromophore with its *meta*-positioned fluorine, shifting the λ_em_ to 515 nm. Thus, encoding and subsequent activation of **ONB2FY**, **ONBDFY**, and **ONB3FY** highlights the magnitude of the effect of the insertion of a single fluorine into a protein.

In another approach to mask near-natural tyrosines for selective incorporation, isotopically labeled **ONBY** was used in NMR structural studies of protein interactions.^152^ Introduction of an isotopically labeled ncAA into an active site greatly simplifies its NMR spectrum, as it provides an “assignment” to the NMR signal for that specific residue.^337^ This can further be used to study the interactions between specific residues of a protein and its substrate, as the chemical shift of the single resonance corresponding to the labeled residue can be tracked.^338^ As native synthetases would recognize an isotopically labeled tyrosine and incorporate it systemically, using an isotopically labeled **ONBY** allowed for site-specific incorporation of the near-natural, labeled amino acid and regeneration of the native, now labeled, protein after irradiation. Isotopically labeled **ONBY** was incorporated into the thioesterase domain of fatty acid synthase and facilitated investigation of the interactions between the protein and an inhibitor.^152,339^

**Figure 24:**
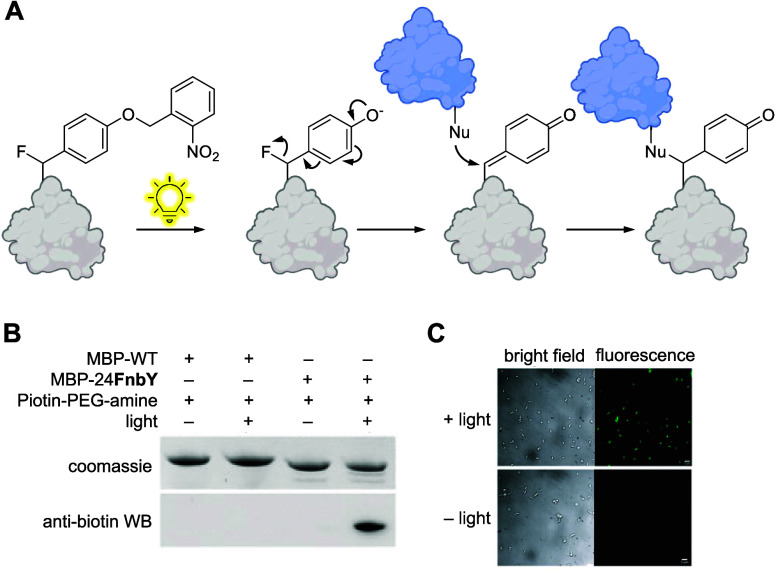
Covalent cross-linking with photocaged tyrosines. (A) After photoactivation, **FnbY** undergoes 1,4-addition with nucleophilic residues to form covalent cross-links between interacting proteins or a protein and a label.^76,77^ (B) Labeling of maltose binding protein (MBP) fused with Z protein containing **FnbY** at residue 24 (MBP-Z-24**FnbY**) with biotin-PEG-amine occurs only after irradiation and decaging of the protein, as observed by antibiotin Western blot (WB).^77^ (C) Labeling of outer membrane protein eCPX-5**FmnbY** with Alexa Fluor 488 hydrazide is dependent upon irradiation of the protein. Panels (B,C) are reproduced with permission from ref (77). Copyright 2020 American Chemical Society.

Another class of photocaged tyrosines includes those that become reactive upon decaging to form covalent cross-links for identification of interacting proteins.^76^ The ncAA *o*-2-nitrobenzyl-β-fluorotyrosine (**FnbY**) was designed to generate a quinone methide (QM) upon photoactivation, which acts as a highly reactive electrophile, creating covalent bonds with proximal nucleophilic amino acids (Figures 15 and 24A).^340^ A main difference between **FnbY** and traditional photo-cross-linkers, such as phenyl azides, diazirines, or benzophenones is the much longer half-life of the resulting electrophile, which is on the scale of seconds rather than nano- or microseconds.^341−344^ Incorporation of **FnbY** into proteins in both *E. coli* and HeLa cells was achieved using an *Mb*PylRS synthetase mutant (FnbYRS, Table 1).^76^ For proof of concept, **FnbY** was incorporated into glutathione transferase (GST) at residue 103, located along the dimerization interface. Irradiation caused covalent binding of **FnbY** with K107 of the opposite GST monomer, as confirmed by mass spectrometry. Mutating its binding partner to other amino acids suggests the ability of **FnbY** to cross-link to other residues as well.

Another application of QM-generating ncAAs was as a general handle for protein modification with nucleophilic molecules.^77^ As **FnbY** and a related ncAA, *o*-2-nitrobenzyl-3-fluoromethyl tyrosine (**FmnbY**, Figure 15), create short linkages between the protein backbone and a nucleophile, they are useful for structural studies examining the distance and orientation of conjugation partners with methods such as NMR, electron paramagnetic resonance, or Förster resonance energy transfer (FRET). An *Ma*PylRS was engineered for incorporation of **FmnbY** (FmnbYRS, Table 1).^77^ Both **FnbY** and **FmnbY** were shown to allow protein labeling with a variety of amine and thiol derivatives through a 1,4-addition. Introduced labels included fluorophores, biotin, and PTM mimics (Figure 24B,C). *In vitro* labeling was also efficient at 0 °C, making these ncAAs ideal for use with unstable proteins that must be kept cold.

Another application of photocaged ncAAs in biomaterials are 3,4-dihydroxyphenylalanine (DOPA)-containing adhesives. DOPA is a molecule found in the adhesive proteins of marine mussels and is responsible for the extreme underwater adhesion capabilities of the organisms.^345,346^ Genetic encoding of ONB-caged DOPA analogues with the caging group at either the *meta* or *para* positions revealed that ***m*****-ONB-DOPA** (Figure 15) displayed more efficient incorporation than ***p*****-ONB-DOPA**.^79^ After exposure to 365 nm light for 45 min, more than 50% of the ncAA decaged; as with other ONB-caged ncAAs, the metabolic reduction of the nitro group was a limiting factor. To further prove the utility of this ncAA, ***m*****-ONB-DOPA** was incorporated into the muscle adhesive protein foot protein-5 (fp-5). Replacing 5 or 10 tyrosine residues in the protein with ***m*****-ONB-DOPA** permitted successful expression of the multicaged protein in a strain of *E. coli* mutated to lack release factor;^347^ however, replacing all 19 of the tyrosine in the protein sequence with caged DOPA prevented the expression of the protein, likely due to competition of release factor 1 or steric clashing between the ncAAs and other residues of the protein.^79^ Atomic force microscopy revealed 12- and 6.5-fold increases in adhesion of the proteins containing 5 and 10 instances of the ncAA, respectively, upon irradiation. These findings highlight the potential of ***m*****-ONB-DOPA** for creating a strong, photoactivatable underwater adhesive, and the possibility for other DOPA analogues to be genetically incorporated into proteins.

Overall, photocaged tyrosines have been used in a broad range of applications to introduce optical control over proteins. Its prevalence in active sites makes tyrosine a prominent amino acid to cage. Through photocaging, tyrosine loses the ability to H-bond, act as a nucleophile, or undergo post-translational modifications, such as phosphorylation.^257−260^ With the many key functions of the native amino acid, photocaging offers a variety of ways to connect the activity of a protein to its irradiation state in addition to just introducing steric bulk into an active site. A range of caged tyrosine analogues have been designed that serve different purposes and allow control over and investigation into the behaviors of many proteins and pathways. While **ONBY** is the most commonly used photocaged tyrosine, its instability in bacterial environments poses room for improvement.^131,153−155^ The reduction of the **ONBY** nitro group to the corresponding amine in prokaryotic cells prevents decaging, which has limited its applicability. The prevalence of this reduction is dependent on the protein of interest and the specific site of introduction; even two sites within the same protein may result in very different amounts of the reduced ncAA.^163^**NPY** serves as an alternate caged tyrosine with efficient decaging and high rates of incorporation, ultimately yielding an improved alternative to **ONBY** for future applications of photocaged tyrosine.^75^ Expression in *E. coli* strains lacking nitroreductases or applications strictly in eukaryotic cells may also decrease the amount of reduced **ONBY**.^261^ Overall, caged tyrosines exemplify the range of possible applications of photocaged ncAAs in introducing optical control over protein function through rational design paired with genetic code expansion.

## 5 Cysteine

Although one of the least abundant amino acids in the proteome, cysteine frequently functions as a nucleophile in enzyme active sites and plays important roles in redox signaling, metal binding, and protein folding through disulfide bond formation.^348^ Selenocysteine (U) is a functionally similar but rare amino acid that has also been targeted for optical control of protein function; it has a lower p*K*_a_, lower redox potential, and higher nucleophilicity compared to cysteine.^349^ By replacing the thiol and selenol protons with light-removable protecting groups, a unified approach to optically controlling the diverse functions of these amino acids has been achieved in proteins.

**Figure 25:**
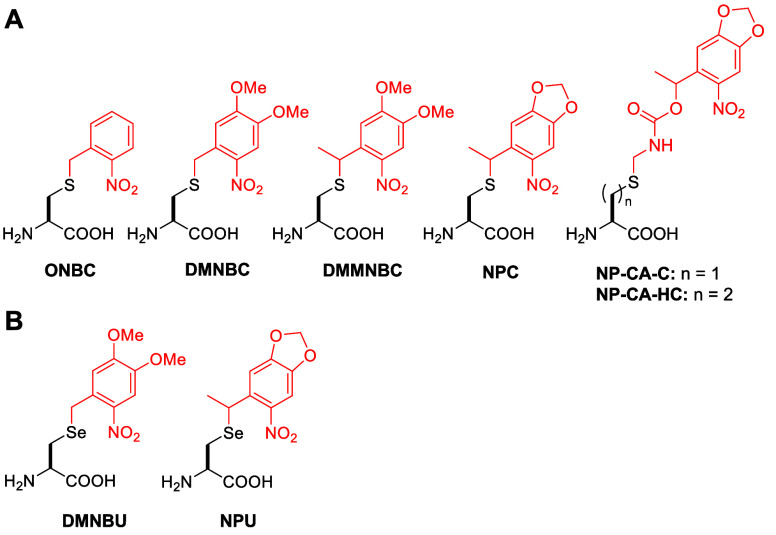
Chemical structures of photocaged (A) cysteines and (B) selenocysteines with caging groups indicated in red.

The first synthetase evolved for a caged cysteine was developed using *Ec*LeuRS/tRNA machinery and was applied in yeast.^53^ The LeuRS was chosen based on analysis of its crystal structure, which features a large hydrophobic pocket, allowing for expected accommodation of diverse unnatural side chains.^53^ Five residues in the LeuRS binding pocket (M40, L41, Y499, Y527, and H537) were randomized and the resulting library was used in a genetic selection with *o*-nitrobenzyl cysteine (**ONBC**, Figure 25A). After three rounds of alternating positive and negative selection using *URA3* and *HIS3* as genetic markers,^350^ respectively, a selective synthetase was obtained (ONBCRS, Table 1).

Separately, **ONBC** was genetically encoded with the PylRS system.^80^ Specifically, the *Mb*PylRS/PylT pair was engineered through randomization of residues around the synthetase binding pocket (N311, C313, V366, W382, and G386) and three rounds of positive and negative selection, yielding PCC1RS (Table 1). Simultaneously, a synthetase for the nitropiperonyl caged cysteine **NPC** was identified and named PCC2RS (Table 1), and was later used to genetically encode the nitropiperonyl caged selenocysteine **NPU**.^86^

Two more photoactive ncAAs, 4,5-dimethoxy-2-nitrobenzyl cysteine (**DMNBC**) and 1-(4,5-dimethoxy-2-nitrophenyl)ethyl cysteine (**DMMNBC**, Figure 25A), were both genetically encoded using *Ec*Leu DMNBSRS (Table 1), a synthetase that was originally used to incorporate photocaged serine **DMNBS** (see section 6).^44^ These caging groups are attractive because methoxy groups provide a slightly red-shifted absorption spectrum relative to **ONBC**. Further, the methyl group present in **DMMNBC** increases the rate of photolysis and yields a less reactive ketone byproduct.^26^ The synthetase DMNBSRS was also used to incorporate 4,5-dimethoxy-2-nitrobenzyl caged selenocysteine (**DMNBU**, Figure 25B).^87,88^

In a different approach, analogues of a nitropiperonyl caged cysteine (**NP-CA-C**) and homocysteine (**NP-CA-HC**, Figure 25A) were specifically designed with aminomethylene linkers so the photocaging group would structurally mimic the photocaged lysine **PCK** (Figure 2).^85^ The goal was to utilize the previously identified PCKRS (Table 1) for incorporation. Expression experiments with PCKRS showed that **NP-CA-HC** was incorporated at similar levels to **PCK**, but **NP-CA-C** was incorporated with slightly reduced yields likely due to is shorter cysteine side chain.^85^

As the main role of cysteine in biology is to act as a nucleophile, several examples exist that utilize a photocaged cysteine to control this functionality, particularly in the context of generating light activated proteases. As the first example of an enzymatically encoded photocaged cysteine, an active site cysteine in human proapoptotic protein caspase 3 was replaced with **ONBC** using the *E. coli*-derived ONBCRS in yeast.^53^ Caspase 3 is a cysteine protease involved in programmed cell death, and typically exists as a zymogen in the cytosol of cells.^53^ When active, C163 interacts with H121 in a catalytic dyad, where histidine abstracts a proton from cysteine, allowing for nucleophilic attack of the peptide substrate.^351^ When recombinant caspase 3 was expressed with **ONBC** at site 163, no activity was observed. After 10 min of irradiation with UV light, protease activity was detected at 40% of wild-type levels.^53^ The requirement for long irradiation times and observed incomplete decaging have caused alternate photocaging groups to be favored over ONB for further caging of cysteine.

The same constitutively active human caspase 3 was also caged with **NPC** to control protease function in *C. elegans*.^84^ Of note, this site directed incorporation of **NPC** used a quadruplet codon rather than the classic triplet codon approach (as discussed in section 3). Mutations matching that of PCC2RS were encoded in an *Mm*PylRS for the genetic encoding of **NPC** in *C. elegans*. Optical control of caspase 3 permitted light-activated apoptosis in worms, including spatial control using cell-specific promoters.^352^

**Figure 26:**
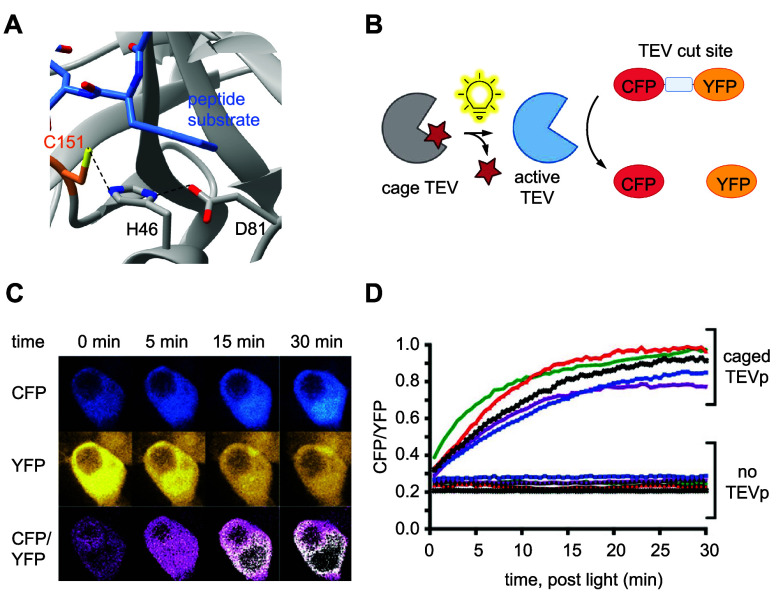
Optical control of protease activity with photocaged cysteine. (A) Active site rendering of the TEV protease catalytic triad. Residue D81 withdraws electron density from H46, activating C151 for nucleophilic attack of the peptide substrate (PBD 1LVM). (B) Optical activation of TEV protease and its cleavage of the CFP-TevS-YFP substrate. (C) Single-cell time course of TEV protease activity after irradiation as detected by a FRET sensor shows an increase in CFP/YFP fluorescence. (D) TEV protease activity for cells expressing the FRET sensor, with or without photoactivated TEV protease. Panels (C,D) reproduced with permission under Creative Commons CC-BY license from ref (80). Copyright 2014 The Author(s).

Another cysteine protease, TEV protease, was also targeted for caging.^80^ TEV protease is a widely used enzyme due to its high substrate specificity and efficiency, and has been utilized across industrial and cellular applications for fusion protein cleavage, tag removal, and purification.^353^ TEV protease has a catalytically active cysteine (C151) which is positioned in a triad with H46 and D81 (Figure 26A).^354^ When TEV protease-C151**NPC** was expressed in HEK293T cells using PCC2RS, a FRET-based reporter bearing a TEV cut site (CFP-TevS-YFP) indicated a completely inactive enzyme. Irradiation with 365 nm light for 1 min recovered TEV protease activity and live cell imaging revealed 80% cleavage of the FRET sensor after 15 min (Figure 26C–D).^80^ A light activatable TEV protease could be a useful system to control engineered outcomes of protease-catalyzed cleavage, such as protein translocation and signal transduction.^355^ TEV protease activity was also controlled using the photocaged tyrosine **NPY**; see section 4.^75^

**Figure 27:**
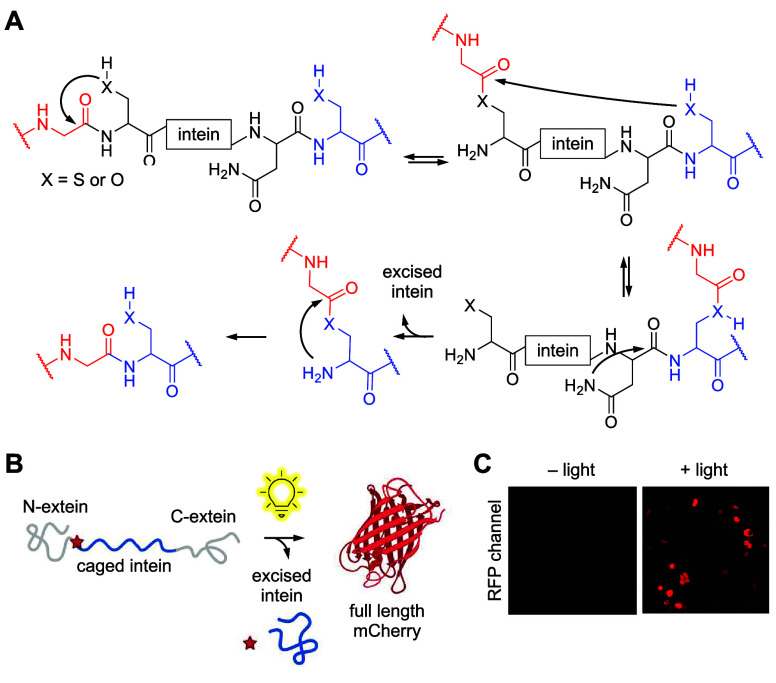
Light regulation of intein splicing via caged cysteine. (A) Splicing mechanism of a *cis*-intein.^359^ (B) Optically controlled mCherry maturation through a photoactive intein splicing event. The photoactivatable small molecule is represented by the red star. (C) Light-activated mCherry formation after a splicing event triggered by UV light in HEK293T cells. Reproduced with permission from ref (118). Copyright 2015 American Chemical Society.

Like proteases, inteins also rely on a nucleophilic cysteine for activity and are therefore good targets for photocaging. Inteins are internal protein segments that are post-translationally excised to splice together flanking sequences, or exteins, through peptide bond formation (see also section 4).^319,356^ Splicing events can result either from split inteins (*trans*-splicing inteins) that contain two fragments that are transcribed and translated independently, or through *cis*-splicing inteins that are expressed as one polypeptide domain.^357^ The splicing process starts with nucleophilic attack of cysteine (or serine) onto an amide carbonyl (Figure 27A). By blocking the nucleophilicity of this cysteine with a caging group, intein splicing was suppressed until photolysis.^118^ The *Nostoc punctiforme* (*Npu)* DnaE intein was selected for caging as it is a well characterized, efficient intein, and is compatible with a wide range of extein sequences.^358^ Placement of this intein within a protein of interest results in the expression of an inactive protein that is then activated by a splicing event (Figure 27B).

To validate the optical control of *Npu* DnaE for the activation of protein assembly, *Npu* DnaE was placed within mCherry so that a cysteine was present at both the N- and C-terminus of the intein, as is required for the second step of splicing, transesterification (Figure 27A).^118,359^ The N-terminal cysteine was mutated to an amber stop codon, and **DMMNBC** was incorporated into this construct using DMNBSRS in HEK293T cells. Before irradiation, nearly no background florescence of the split mCherry was seen; however, red florescence was observed within 1 h after irradiation, aligning with the rate of mCherry chromophore maturation (Figure 27C).^118^ The use of photocaged inteins may provide a general method that could theoretically place any protein of interest, regardless of its active site residues or caging compatibility, under optical control. Unfortunately, there are limitations to this approach. For example, one risk of expressing an intein-containing protein is that it may misfold and result in an inactive protein product or the formation of inclusion bodies in the case of bacterial expressions.^319,360^ Further, the need for a cysteine at the N-terminus of the C-extein may require additional mutations to the protein of interest.

As a proof-of-concept, the human tyrosine kinase Src was controlled using this photoactivatable intein (for an in-depth discussion of optical control of kinases, see section 3). Src is a proto-oncogene involved in cell proliferation and growth, and Src pathway activation is seen in upward of 50% of tumors derived from colon, liver, lung, and breast cancers.^361^ The caged DnaE was placed within the sequence of Src at three sites. As previously stated, a strategic cysteine must be present; thus, two of the selected sites utilized native cysteine residues, generating a wild-type Src protein after the splicing event, while the third site utilized an S to C mutation. Src activity was assayed using a KRas-Src sensor based on FRET signal between ECFP and YPet, where kinase activity results in a decrease in the YPet/ECFP ratio.^362^ When applied in HEK293T cells, all three Src mutants showed no activity in the absence of light. After irradiation with 365 nm light for 10 min, wild-type activity was restored as measured by decreasing FRET ratios, demonstrating successful intein splicing and protein activation.

**Figure 28:**
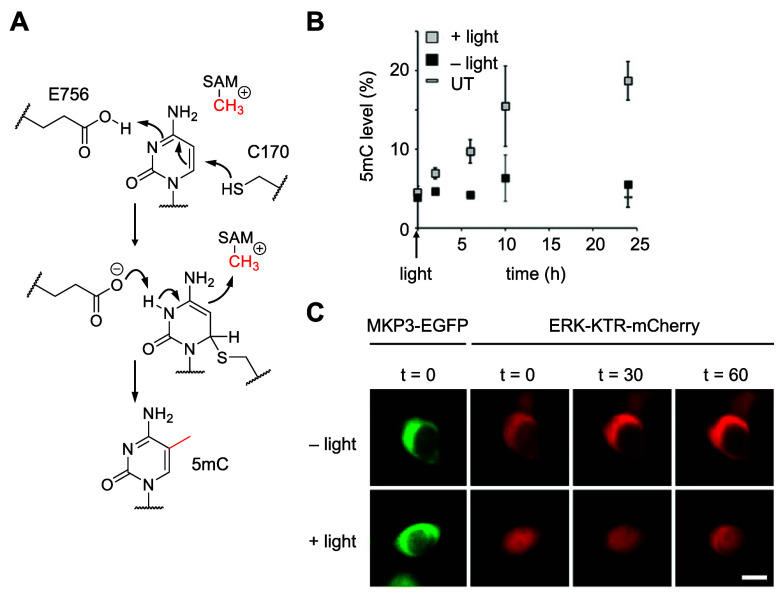
(A) The mechanism of cytosine methylation by DNMT.^157^ (B) SATIII methylation kinetics by SATIII-caged DNMT. Reproduced with permission from ref (157). Copyright 2021 John Wiley and Sons under Creative Commons CC BY license. (C) EGF-activated HEK293T cells expressing photoactivatable MKP3 (green fluorescence) and the KTR reporter (red fluorescence). Reproduced with permission from ref (139). Copyright 2019 Springer Nature under Creative Commons Attribution 4.0 International License.

DNA methyltransferases (DNMTs) are another class of proteins that rely on cysteine for catalytic activity. DNMTs introduce 5-methylcytosine (5mC) into DNA, a modification that is important for control of gene expression. DNMTs use a catalytic cysteine (C710) for activation of the cytosine nucleobase via 1,4-addition, which then attacks the methyl group of the S-adenosylmethionine (SAM) cofactor at the C6 atom (Figure 28A). DNMT3a is responsible for *de novo* methylation and is commonly mutated in hematological malignancies, although the effects of mutations on this protein are not well understood *in vivo*.^363,364^ The catalytic domain of DNMT3a was fused to its activating partner DNMT3L to render it constitutively active, and the active site cysteine was photocaged.^365^ Colon cancer cells with native DNMT1 and DNMT3b genes knocked out were cotransfected with *E. coli* machinery DMNBSRS and the DNMT-C710TAG fusion construct. Successful amber suppression with **DMNBC** was read out through anti-HA immunostaining and flow cytometry. Further, replacement of C710 with **DMNBC** inactivated the DNMT construct, and illumination with UV light for 5 min recovered activity, as measured by anti-5mC immunostaining. Optical control of DNMTs provides high temporal resolution and tunability not offered by small molecule transcriptional modulators toward the ultimate goal of understanding early translational responses of methylation with little to no background.^157^

As a first application of photoactivatable DNMT, a range of cancer-associated mutations (S714C, R736H, R771Q, or R836A) were made in caged DNMT3a, and the relative activities of these mutants were compared 24 h after light activation.^366^ Next, the light activated DNMT was applied for selective 5mC formation through fusion to a transcription-activator-like effector^211^ targeted to the sequence of satellite III (SATIII) DNA, which shows unusual methylation in multiple cancers.^211,367^ Prior to irradiation, the light activatable DNMT diminished background methylation, allowing for a time-resolved analysis of site-specific methylation over 24 h (Figure 28B,C). A unique advantage of using light as a trigger is the ability to strictly control the dose of activation, which could be useful for controlling the strength of the 5mC-downstream effects.

Further, because the ultimate effect of DNA methylation is gene downregulation, light controlled 5mC formation was also used to investigate early transcriptional responses.^366^ DNMT3a-C710**DMNBC** was light-activated in HEK293T cells and mRNA-seq was conducted 0, 4, and 8 h later. As a result, insight was gained into the initial targets of photocontrolled DNMT3a and the transcriptome response to *de novo* methylation. This approach can also be applied to control other DNMTs, as many share catalytic activity.

The first example of optical control of a protein phosphatase was developed using caged cysteine incorporation. MKP3, or mitogen-activated protein kinase (MAPK) phosphatase, was optically regulated by photocaging the catalytic cysteine C293 with **DMNBC**.^139^ The thiol acts as a nucleophile in the dephosphorylation reaction of its target pERK, alongside an arginine that stabilizes the phosphate group in the transition state.^139^ The MAPK signaling pathway is described in section 3, and has been extensively investigated through incorporation of photocaged ncAAs. MKP3 recognizes phosphorylated ERK (pERK) and dephosphorylates it at two sites to prevent nuclear inclusion and downstream gene activation.^368−370^ To improve understanding of the role that MKP3 dephosphorylation plays in the MAPK pathway, MKP3-C293**DMNBC** fused to HA-tagged GFP was expressed in HEK293T cells using DMNBSRS.

To investigate the photoactivatable dephosphorylation of pERK by MKP3, an ERK activity reporter, ERK-KTR-mCherry (similar to the reporter described in Figure 10B), was used.^224^ Prior to ERK phosphorylation, the reporter remains in the nucleus due to an active NLS. Upon generation of pERK, KTR is phosphorylated twice to both deactivate the NLS and activate the NES, resulting in nuclear exclusion. After pathway stimulation with epidermal growth factor (EGF) and before light activation, the reporter was transported out of the nucleus as the inactive MKP3 allowed for continued pERK generation and downstream phosphorylation of the reporter. These results closely mimicked the translocation of the reporter when no MKP3 was present, indicating complete deactivation prior to light irradiation, as quantified by the cytoplasm/nuclear (C/N) ratio of red fluorescence. After light activation, MKP3 then dephosphorylated pERK and prevented its activity on the reporter, resulting in maintained nuclear inclusion, as seen in the bottom panels of Figure 28C. The C/N ratio observed upon decaging mimicked that of wild-type MKP3 expression, indicating near complete decaging. In addition to optically controlling catalytic activity with photocaged cysteine, the interaction between MKP3 and its substrate was rendered light activatable using a caged lysine, as discussed in section 3. Compared to prior methods of phosphatase deactivation, such as gene knock down, the introduction of optogenetic methods through photocaged ncAAs allows for tunability dependent on light exposure and greater control over temporal activation. Further, this approach is generalizable to a wide range of similar phosphatases.

Light-control of the nucleophilicity of cysteine can not only be used to modulate protein activity, but also to control protein conjugation reactions.^83^ Ubiquitination is a biological process that results in covalent attachment of ubiquitin to a protein of interest, with roles in protein degradation, localization, kinase activation, and more.^371^ To study the role of ubiquitin, homogeneously ubiquitinated protein is required but difficult to achieve, especially for monoubiquitinated proteins. A photocaged cysteine was used to direct ubiquitin ligation to a single site on a protein of interest that has many solvent exposed cysteines, taking advantage of differential protecting group chemistry.^83^

**NPC** was incorporated into proliferating cell nuclear antigen (PCNA) at the site of ubiquitination (K164) using the synthetase PCC2RS in *E. coli*.^83^ After purification of PCNA-K164**NPC**, the remaining surface cysteines were protected with methyl methanoethiosulfonate. Following treatment with an alpha halogen modified ubiquitin generated through expressed protein ligation,^372^ nonirradiated protein showed no ubiquitination, as observed through a gel shift assay. After 1 min irradiation with UV light, 80% conversion to the ubiquitinated product was achieved. Finally, all cysteines were deprotected with DTT to result in a permanently monoubiquitinated protein for further study. This method is likely to be generally applicable to other proteins of interest and is an interesting use of orthogonal protecting groups within molecular biology to control protein side chain reactivity.

Caged cysteine has also been used to control protein activity through simple steric occlusion of an active site as seen in the application of the extended linkage utilized in **NP-CA-HYC** and **NP-CA-C**.^85^*Renilla* luciferase (Rluc) was chosen as a target due to its easily read out activity, and incorporation of both **NP-CA-HYC** and **NP-CA-C** with the synthetase PCKRS resulted in an inactive enzyme in HEK293T cells. After illumination for 4 min with 365 nm light, **NP-CA-C** showed a higher increase of function (150-fold) than **NP-CA-HYC** (20-fold), even though incorporation efficiencies of **NP-CA-HYC** were more favorable. This is likely because the extra CH_2_ present in homocysteine still perturbed the active site after decaging. The site-specific incorporation of photocaged homocysteine may be applied to understand the steric limitation and reaction trajectories of other cysteine-based active sites in the future.

**Figure 29:**
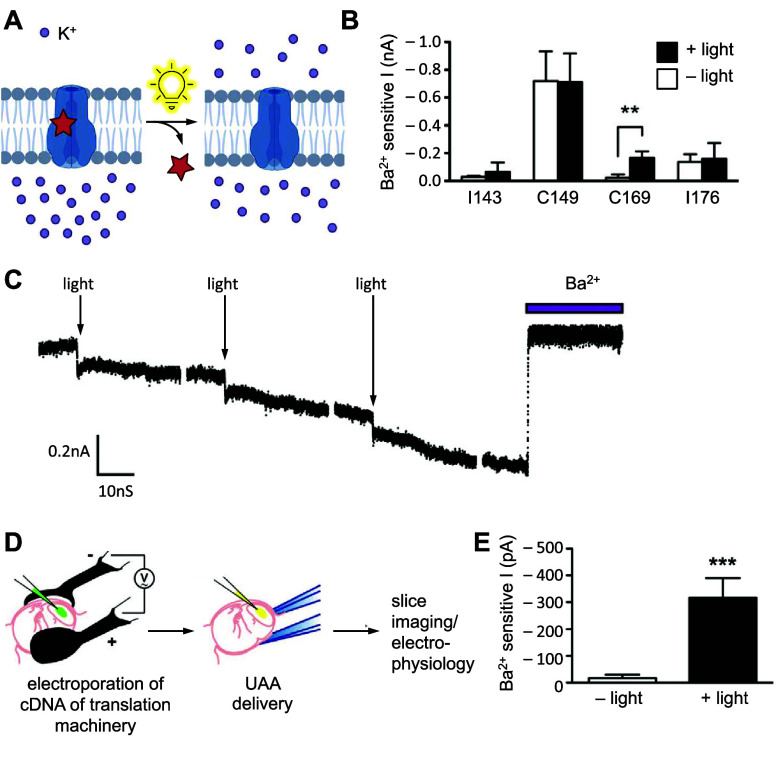
Photocontrol of a potassium ion channel. (A) Kir2.1 channel function before and after photoactivation where the red star indicates photocaged cysteine.^81^ (B) HEK293T cells expressing different mutated Kir2.1 channels with incorporated **DMNBC** before and after light irradiation.^81^ (C) Partial Kir2.1 activation is dependent on the number of light pulses applied. (D) Cartoon depiction of the delivery method of genetic code expansion machinery into the mouse brain. (E) Light activation of Kir2.1 in a mouse brain as seen by a voltage sensitivity to Ba^2+^ after light activation. Panels B, C, D, and E are reproduced with permission from (81). Copyright 2013 Elsevier.

**DMNBC** has also been utilized for the steric occlusion of potassium channel function, specifically of Kir2.1, for precise control of activation in an embryonic mouse neocortex (Figure 29A).^81^ This also represents one of only few applications of ncAAs in mammalian neurons or mouse brains. Photocontrol of Kir2.1 was possible as cysteine introductions at multiple sites within the intermembrane channel had no effect on activity, meaning the system would accommodate a cysteine scar after photolysis. Several sites (T142, I143, D172, I176, A184, and E224) and two native cysteine residues (C149 and C169) were mutated to a TAG amber stop codon for the incorporation of **DMNBC** using the synthetase DMNBSRS. Kir2.1-C169**DMNBC** was selected for further investigation, as no channel activity was observed for the caged protein until irradiation in HEK293T cells, as measured by whole cell voltage sensitivity to Ba^2+^, an inhibitor of Kir2.1 activity (Figure 29B).^373^ It was determined that ion channel activity could be tightly tuned by altering the duration of irradiation and the number of light pulses applied (Figure 29C).

After developing this photoactivatable Kir2.1 channel in mammalian cells, experiments were then conducted *in vivo*. Up to this point, genetic code expansion had not been shown in a mammalian brain.^81^ First, plasmids encoding the ncAA machinery and the protein of interest were electroporated into the mouse embryo *in utero*, and 2 days later, the ncAA was injected directly into the lateral ventricle of the mouse brain (Figure 29D). **DMNBC** was used to control the Kir2.1 channel *in vivo* by electroplating three plasmids *in utero*: one encoding the photocaged Kir2.1 and the tRNA, the second encoding the synthetase and mCherry, and the third encoding EGFP-Y182TAG. Green fluorescence in cells confirmed the delivery of all three plasmids and suggested that Kir2.1-**DMNBC** was also expressed. The successful incorporation of **DMNBC** into Kir2.1 was further confirmed by whole-cell readings of brain slices, which showed no Ba^2+^ sensitivity prior to light activation, and upon irradiation, showed a significant increase in Ba^2+^ sensitivity (Figure 29E). As a proof of concept for genetic code expansion in a mammalian brain, this method expanded the toolbox for neuroscience research and could be applied to investigate other ion channels and ncAAs in mammals. Genetic code expansion in mice was subsequently applied to a photocaged lysine (section 3) and a photoswitchable azobenzene-phenylalanine (section 9).^374,375^

In addition to caged cysteine, caged selenocysteine incorporation has also been achieved. Proteins engineered with selenocysteine can yield enhanced enzymatic activity and add new sites for folding, dimerization, and labeling.^376^ Natively, selenocysteine is incorporated into proteins through selenocysteine insertion sequences, or SECIS, that must be in proximity to a UAG codon.^377^ The tRNA^[Ser]Sec^ is aminoacylated with a serine first, which is then converted to a selenocysteine.^377^ The complex process of selenocysteine incorporation into proteins has resulted in difficulties overexpressing these targets. Through genetic encoding of a photocaged selenocysteine, a generalizable method for the synthesis of seleno-proteins was developed.

In addition to providing a recognition handle for the synthetase, the caging groups protect against undesirable side reactions (U–C or U–U bridge formation) in the cell. Using the synthetase DMNBSRS, **DMNBU** was successfully incorporated into EGFP at residue Y39, a solvent exposed permissive site, in yeast cells.^88^ A time course of photolysis over 80 min showed that irradiation with 330–385 nm light resulted in greater than 95% decaging, as observed by HPLC and mass spectrometry. Further, it was demonstrated that only after decaging was protein dimerization via a diselenide bond possible, which was demonstrated through semi-native SDS-PAGE.

A photocaged selenocysteine bearing a nitropiperonyl group (**NPU**, Figure 25B) was also encoded in *E. coli* using the synthetase PCC2RS (Table 1).^86^ Peptidyl-prolyl *cis*-*trans* isomerase B (PpiB) and the Zika virus NS2B-NS3 protease (ZiPro) were selected as recombinantly expressed targets and a decaged, solvent exposed selenocysteine was labeled with trimethylsilyl. Decaged selenocysteine sites were selected proximal to the active site of the enzymes, which was important for subsequent protein NMR experiments after site specific labeling. **NPU** incorporation and selective labeling after decaging (365 nm light, 60 min) of the seleno-protein was confirmed through mass spectrometry and NMR studies.

The most recent application of a photocaged seleno-protein extends its applications into mammalian systems using **DMNBU** and synthetase DMNBSRS in HEK293T cells.^87^ This is also the first example of a photocaged selenocysteine placed within the active site of a protein. After demonstrating function of the synthetase for **DMNBU** by expressing EGFP-Y39**DMNBU** and validating decaging (365 nm, 5 min) through mass spectrometric analysis, **DMNBU** was incorporated into a known seleno-protein, MsrB1. This protein utilizes an active site selenocysteine for reduction of methionine-*R*-sulfoxide and engages in downstream signaling and labeling.^378^ The active site selenocysteine (U95) forms a catalytic triad with R93 and D85, thus controlling MsrB1 activity.^379^ In cells, **DMNBU** decaging was monitored by isotopically labeling the now reactive selenocysteine followed by mass spectrometry. The activity of caged MsrB1 after light exposure was confirmed in a biochemical assay after pull-down. Unfortunately, a dark control was not included, making it impossible to assess if the caged enzyme was inactive. As this was a proof-of-concept study showing that photocaged selenocysteine can be incorporated into proteins in mammalian cells, applications of optical control of seleno-proteins can be expected in future studies.

Because cysteine is essential in the function of many enzymes and is important for metal binding, protein folding and protein–protein interactions through disulfide bond formation, redox signaling, and nucleophilic attack of a substrate, there is a wide range of potential targets that can be placed under light control. Selenocysteine photoactivation is a highly underexplored topic, and further investigations into the consequences of seleno-protein temporal activation are needed.

## 6 Serine

Serine bears a polar side chain and is important as a nucleophilic active site amino acid. It engages in a catalytic triad with aspartate and histidine and is often post-translationally modified through phosphorylation and o-glycosylation.^380,381^ One prominent enzyme class that relies on serine for activity is the serine protease.^382^ Generation of a photoactivatable serine is an attractive tool for the conditional control of protein activity.

Thus far, only one photocaged serine has been incorporated into proteins via genetic code expansion. Here, a 4,5-dimethoxy-2-nitrobenzyl (DMNB) photocaging group was utilized, as the methoxy groups red-shift the absorption spectrum relative to the common *o*-nitrobenzyl group.^44^ A synthetase selective for **DMNBS** was developed in yeast using an orthogonal *Ec*LeuRS/LeuT pair. The tRNA was mutated to an amber suppressor and the synthetase was engineered through randomization of five residues near the leucine binding pocket (M40, L41, Y499, Y527, and H537) and a subsequent double-sieve selection.^53,383^ The synthetase was tested through the incorporation of **DMNBS** into human superoxide dismutase but showed background incorporation of natural amino acids.^53^ Endogenous amino acid recognition was rectified by adding a T252A mutation, which has shown to reduce tRNA acylation with leucine and isoleucine.^384,385^ This resulted in the development of DMNBSRS (Table 1), a highly selective synthetase for **DMNBS** over endogenous amino acids, as confirmed by mass spectrometry analysis of expressed proteins.^44^ DMNBSRS has also been used to encode photocaged cysteine and selenocysteine (see section 5).

**Figure 30:**
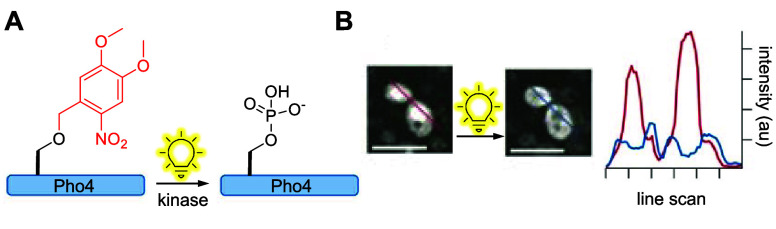
Photocaged serine for optical control of transcription factor function. (A) Optical control of a serine phosphorylation site of Pho4.^44^ (B) Fluorescence micrographs and corresponding line scans show nuclear inclusion of photocaged Pho4-GFP and loss of nuclear florescence after photolysis and kinase-mediated phosphorylation. Reproduced with permission from ref (44). Copyright 2007 Springer Nature.

An early application for **DMNBS** was the photoregulation of phosphorylation, first demonstrated using Pho4, a transcription factor in yeast (Figure 30A).^44^ Pho4 is shuttled between the cytoplasm and the nucleus based on phosphorylation at two sites: S114 and S128.^386^ Site-specific caging of each of these sites represents a clever approach to gain a deeper understanding of how differential phosphorylation controls Pho4 export from the nucleus.^44^ Incorporation of **DMNBS** into Pho4-GFP at each site blocked phosphorylation, resulting in GFP accumulation in the nucleus, validating that **DMNBS** is recognized as a nonphosphorylated serine in the cell (Figure 30B). Further, after decaging with 488 nm light, Pho4 was phosphorylated and completely transported out of the nucleus, showing near wild-type recovery. The export rates after phosphorylation at each site were compared, revealing that decaged Pho4-S114**DMNBS**-GFP had slower export kinetics than S128**DMNBS**. To further isolate the effects of phosphorylation at a single site, serine caging was paired with alanine mutations. This first application of **DMNBS** demonstrated that phosphorylation can be conditionally controlled in cells with this caged ncAA.

**Figure 31:**
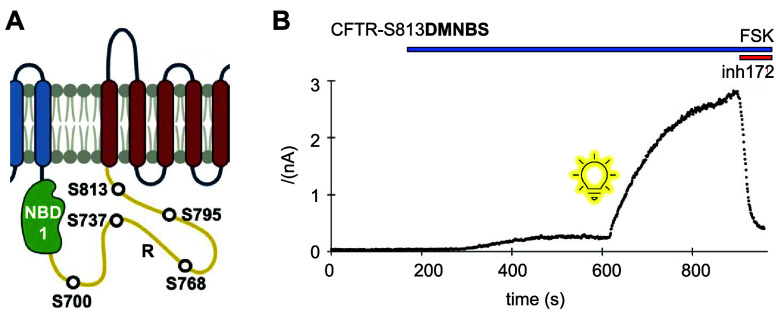
Photocontrol of CFTR phosphorylation. (A) Cartoon representation of CFTR with the R domain shown in yellow and all investigated phosphorylation sites highlighted. (B) Whole-cell current of CFTR-S813**DMNBS** over time. The blue bar represents addition of forskolin (FSK) to induce CFTR activity. Light activation is indicated by the yellow light bulb at 600 s. The red bar represents the addition of CFTR inhibitor 172 (inh172). Reproduced with permission from ref (119). Copyright 2023 Rockefeller University Press.

Recently, **DMNBS** was used to control phosphorylation at sites in cystic fibrosis transmembrane conductance regulator (CFTR), a protein which causes cystic fibrosis when mutated.^119,387^ CFTR is a transepithelial channel responsible for regulating Cl^–^ flow and plays important roles in fluid and electrolyte secretion, sweat gland and airway epithelia, and electrolyte absorption.^388^ Phosphorylation of CFTR occurs at multiple sites, making it difficult to discern their individual consequences on activity. One such region of heavy phosphorylation is the R domain of CFTR (Figure 31A).^389^ Photocaged serine is an attractive tool to study CFTR phosphorylation because data suggest that alanine mutations in the R domain may more closely mimic phosphorylated CFTR than not.^390^ Furthermore, PKA (protein kinase A), the kinase responsible for the phosphorylation of CFTR, can activate the channel by simply binding to the R domain without phosphorylation, and **DMNBS** may interrupt this binding through steric hindrance.^391^

The activity of CFTR was assessed by whole cell voltage.^392^ Alanine mutations previously revealed that mutating S700, S795, and S813 caused a decrease in channel opening, while mutations at S737 and S768 resulted in an increase in activity.^389^ An S813**DMNBS** mutation led to dramatically reduced channel activity (<10% of wild-type).^119^ Following decaging with a 250 ms pulse of 385 nm light, channel activity increased 12-fold over ∼3 min due to phosphorylation, supporting previously determined activation kinetics as seen in Figure 31B.^393^ Further, protein binding studies showed that **DMNBS** sterically hinders PKA binding.^119^ This suggested that both the lack of phosphorylation and inhibited binding of PKA may contribute to the silencing of CFTR when **DMNBS** is incorporated at S813.

The other previously mentioned sites (S700, S795, S737 and S768) were also mutated to TAG stop codons for the incorporation of **DMNBS**.^119^ Photocontrol of these sites revealed interplay between phosphorylation of different serines in CFTR activation and is an example of how photocaged serine can be used to better understand complex post-translational modification events. The techniques developed for CFTR photocaging can likely be applied to other membrane proteins with activities that have a strong connection to phosphorylation.

As well as being used to block phosphorylation, the bulky caging group of **DMNBS** lends its use to sterically hinder the active site of proteins. 5-Methylcytosine (5mC) is important for genomic regulation and both hyper- and hypomethylation is present in cancers.^394^ In mammalian cells, ten-11-translocation (TET) dioxygenases catalyze DNA demethylation.^395^ For example, TET2 is mutated in multiple cancers and alters the level of 5mC oxidation, affecting the availability of genes for expression. Understanding the downstream effects of TET mutation and its impact on differentiation and transformation to malignancy requires the precise control of protein function, which has previously been limited to the use of small molecule inhibitors, which presents challenges relating to selectivity.^111^

**Figure 32:**
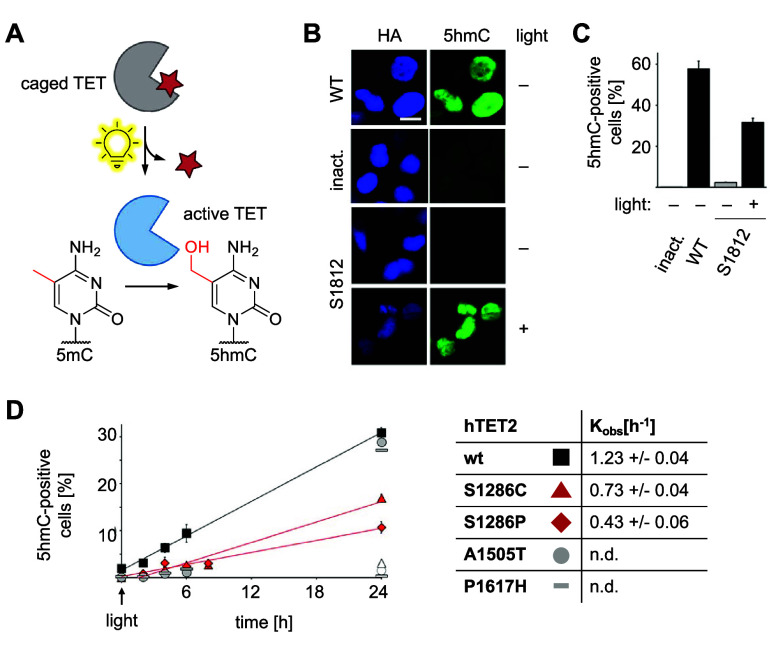
Light control of TET dioxygenase activity. (A) Optical control of 5mC oxidation through hTET2 photocaging with **DMNBS**. (B) Anti-HA and anti-5hmC coimmunostaining micrographs before and after photolysis of **DMNBS**, along with wild-type and inactive wild-type controls. (C) Analysis of 5hmC levels in cells with photocaged mTET2 by anti-5hmC immunostaining and flow cytometry before and after photolysis. (D) Kinetics of 5MC formation of selected hTET2 mutants after light activation. Panels (B,C,D) are reproduced with permission from ref (111). Copyright 2020 American Chemical Society.

To develop a light activatable TET2, different active site serine residues were replaced with **DMNBS** to inhibit catalysis and cofactor binding.^111^ This inactive TET would lead to limited 5hmC formation, and upon irradiation, the regenerated active TET would stimulate 5mC oxidation (Figure 32A). First, using murine TET2 (mTET2) as a model, S1812 was selected for mutation to **DMNBS** because, according to computational modeling experiments, it would completely displace alpha-ketoglutarate and alter the position of iron and 5mC in the active site. Interestingly, mTET2-S1812**DMNBS** had little to no background activity prior to irradiation (Figure 32B) and displayed the highest recovery of the different mutations made (55% of wild-type, Figure 32C), as read out by 5hmC immunostaining and flow cytometry.^111^

After caging and light activation of mTET2 was demonstrated, the investigation moved to human TET2 (hTET2). The respective TAG site was introduced at S1898 and **DMNBS** was encoded.^111^ After saturation and light activation of hTET2-S1898**DMNBS**, a linear formation of 5hmC over time was observed in HEK293T cells. Known malignant mutations were added and their effects on TET2 function were assessed after decaging (Figure 32D). Importantly, this investigation revealed the effects of multiple previously uncharacterized mutations on the activity of TET2 in cells. Because of the high control over background activity of TET2 afforded by **DMNBS**, previously unreported intricacies in the initial reaction rate of TET2 mutants were revealed. As 5mC is an important factor in chromatin regulation for control of DNA transcription, photocaged hTET2 was also used to monitor gene upregulation in response to light activation.^111^

Based on the previous success of photocaging of TET2, TET1 was also caged with **DMNBS**.^110^ Similarly to TET2, TET1 is important for the regulation of 5mC oxidation and relies on oxygen, iron, and alpha-ketoglutarate for activity.^110^ A second class of enzyme, methyl-CpG binding domain (MBD) containing proteins, act alongside TETs to read for cytosine methylation in DNA.^396^ The interactions between these proteins for chromatin regulation is an important process in cells, and understanding this interplay is vital for studying mechanisms related to diseases. Again, **DMNBS** was useful for turning on the activity of TET1 to measure the levels of oxidized 5mC after irradiation with no background and was used to elucidate the interplay of TET and MBD in 5mC oxidations.^110^

The development of a photocaged serine has contributed to a deeper understanding of biological processes, not only through blocking and monitoring of the kinetics and effects of phosphorylation, but also through steric hindrance at the active site of proteins to precisely control activity. Although the DMNB caging group has been shown to be effective, there is room for improvement. For example, the addition of an alpha methyl group would expedite decaging and yield a less reactive acetophenone byproduct.^68,143,169^ Decaging could be further facilitated through installation of a carbonate linker, which in turn would enable the use of a coumarin caging group with a further red-shifted absorption spectrum.^26^ It would be of interest to expand the scope of targets for photocaged serines to include enzymes such as serine proteases.

## 7 Histidine

**Figure 33:**
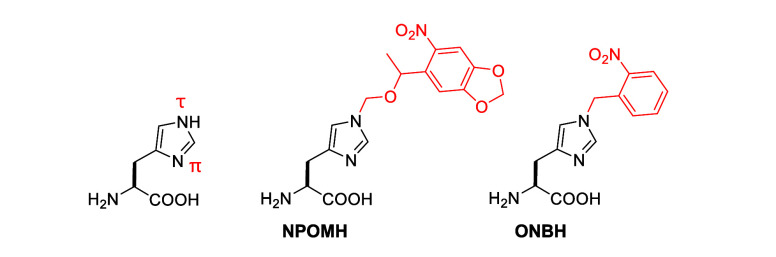
Histidine structure with nitrogen atoms labeled, and genetically encoded photocaged histidine structures with caging groups indicated in red.

Histidine is a vital amino acid in many proteases and metalloproteins and is important for a range of functions such as protein folding, protein–protein interactions, and is critical as a general base to catalyze biological reactions.^397^ Its diverse roles arise from its unique molecular structure, as the imidazole ring (Figure 33) is both aromatic and slightly acidic with a p*K*_a_ = 6.5. This aids its ability to interact with other amino acids, small molecules, and metal cations through hydrogen bond donor and acceptor relationships, π–π stacking, cation-π interactions, hydrogen-π interactions, and coordinate bond interactions.^397^ Surprisingly, the genetic incorporation of caged histidine has been sparse. Recently, two photocaged histidines have been successfully incorporated into proteins utilizing the 6-nitropiperonyloxymethyl (**NPOMH**) and *o*-nitrobenzyl (**ONBH**) caging groups attached at the τ nitrogen of the histidine ring (Figure 33).

The only photocaged histidine that has been incorporated into proteins using an engineered tRNA synthetase is **ONBH**, an ncAA with a protecting group that is not ideal for nitrogen heterocycles due to low decaging yields.^398^ Upon HPLC analysis prior to genetic incorporation, it was found that decaging of this molecule yields roughly 30% recovery of histidine after 30 min of irradiation with 365 nm light in aqueous solution.^89,399^ Although decaging was slow, an orthogonal synthetase was selected for **ONBH** through directed evolution of an *Mb*PylRS to randomize active site adjacent amino acids (N311, C313, V366, W382, and G386).^89^ After multiple rounds of double-sieve selections, an efficient synthetase for **ONBH** (ONBHRS, Table 1) was developed.

The synthetase was validated through expression of sfGFP-Y151**ONBH** and Ubiquitin-K11**ONBH** in *E. coli*. Incorporation and decaging of **ONBH** were confirmed by whole protein mass spectrometry. While studies in *E. coli* suggested high specificity of the synthetase for **ONBH**, incorporation experiments in mammalian cells using an mCherry-TAG-EGFP reporter revealed background incorporation of endogenous amino acids.^89^

Next, decaging of blue fluorescent protein (BFP) containing **ONBH** was tested. The BFP mutant H66**ONBH** was expressed, as previous experiments using ONB-caged tyrosine at the Y66 position in GFP resulted in quenched fluorescence likely due to steric interference with β-sheet packing and photon-induced electron transfer from the fluorophore to the ONB group.^113^ BFP-H66**ONBH** displayed reduced fluorescence until light-exposure, although activation reached only 10% of wild-type BFP fluorescence even after a 5 min irradiation at 365 nm, reflecting the limited decaging seen by HPLC.^89^ Longer irradiations resulted in photobleaching of BFP.

**Figure 34:**
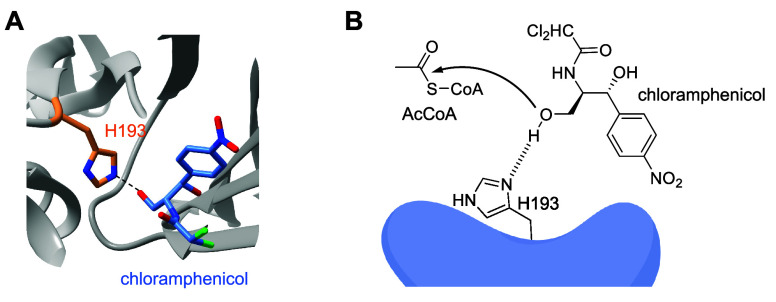
Photocaged histidine for optical activation of chloramphenicol acetyltransferase. (A) CAT_I_ active site H193 hydrogen bonding with the hydroxy group of the chloramphenicol substrate (blue; PBD 3U9F). (B) Light-activated acetyltransferase showed about a 12% recovery when reaction rates were compared to that of wild-type enzyme. Reproduced with permission from ref (89). Copyright 2023 John Wiley and Sons.

Although decaging yields of **ONBH** were low, enzymatic activity of type I chloramphenicol acetyltransferase (CAT_I_) was controlled by incorporating **ONBH** at the catalytic residue H193.^89^ This histidine acts as a general base and engages in important hydrogen bonding to stabilize the CAT_I_ substrate, chloramphenicol, in its binding pocket (Figure 34A).^400^ Caged CAT_I_ showed no background activity, importantly; however, upon decaging with 365 nm light for 5 min, the highest recovered activity was 12% that of the wild-type enzyme (Figure 34B).^89^ After 10 min of irradiation, the activity of the enzyme began to decrease, likely due to protein damage from extended exposure to UV light. Both experiments demonstrate the utility of caging a histidine residue to control side chain interactions such as hydrogen bonding and acid/base activity, which are main functions of histidine in proteins. However, optimization of the ncAA is needed as such a low decaging yield limits the control over enzyme activity. To apply this ncAA in mammalian cells, *Renilla* luciferase (Rluc) was investigated as a proof-of-principle target for photocaging experiments. When H285 was replaced with **ONBH**, Rluc activity was decreased to nearly zero and showed roughly a 3-fold increase in activity when irradiated with 365 nm light.^89^

While ONB showed limited applicability in the caging of His, the NPOM caging group exhibited highly favorable decaging yields of 97%, presumably due to the CH_2_O functionality being a better leaving group.^398,401^ Surprisingly, synthetase screens for the genetic encoding of **NPOMH** (Figure 33) have failed. This is likely due to steric restrictions of the synthetase binding pocket regarding alterations to an amino acid core.^402^ To circumvent the limitations of synthetase engineering, chemical acylation was used as an alternative method to incorporate **NPOMH** into proteins *in vivo*.^108^ Chemical acylation was introduced as the first method of *in vitro* ncAA mutagenesis in 1989 and relies on the synthesis of an amino-acylated tRNA, bypassing the need to develop a selective orthogonal synthetase.^403^ Chemically acylated tRNAs have been shown to be functional for cell-free synthesis, in cells, and in unfertilized frog oocytes.^108,404^ In 2023, it was demonstrated that chemically acylated tRNAs are active in zebrafish embryos and enabled the first incorporation of **NPOMH** into proteins *in vivo*.^108^

**Figure 35:**
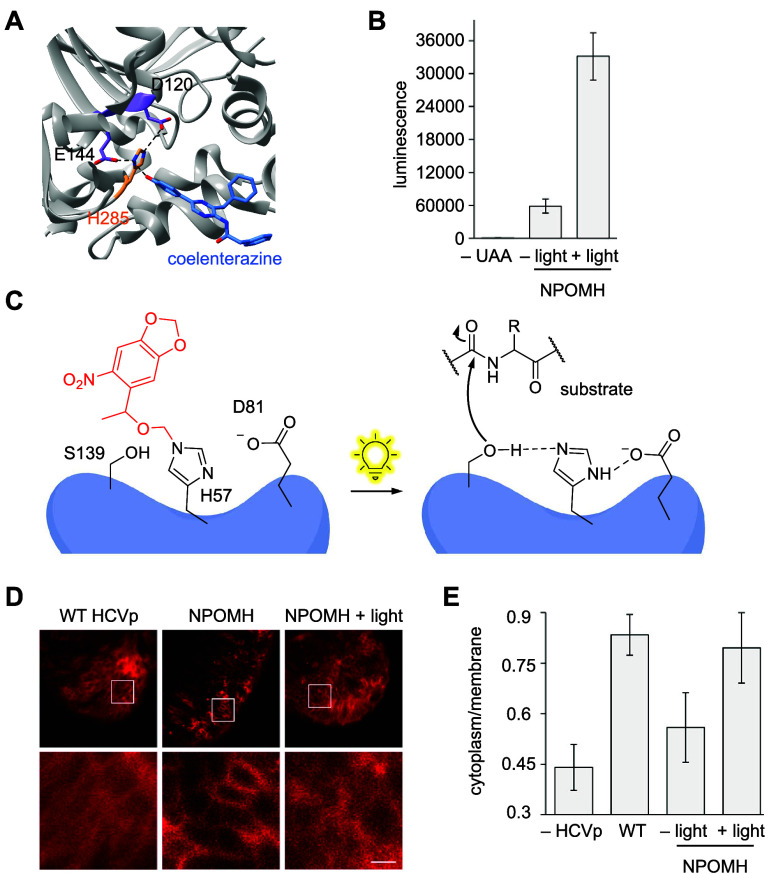
Optical control of protein activity though genetically encoded **NPOMH**. (A) Active site rendering of Rluc where H285 hydrogen bonds to the substrate coelenterazine (blue) and engages in a catalytic triad with E144 and D120 (purple) to assist with oxidative decarboxylation (PBD 2PSJ). (B) Quantification of photoactivation of Rluc in zebrafish embryos. (C) Photocontrol of a histidine within the catalytic triad of a protease active site. (D) Confocal images of zebrafish brain cells expressing HCVp and a membrane translocation reporter. (E) Quantification of the cytoplasm/membrane red florescence before and after light activation. Panels (B,D,E) are reproduced with permission from (108). Copyright 2023 American Chemical Society.

Using Rluc-H285**NPOMH**, optical control of protein function in zebrafish was demonstrated. Rluc oxidizes its coelenterazine substrate, leading to photon emission.^405^ In the Rluc active site, H285 acts as a general base to assist with oxidative decarboxylation, and has been shown to be essential for protein function based on alanine mutation.^405,406^ Further, the structure of the active site suggests that H285 plays a role in a catalytic triad with D120 and E144 (Figure 35A). Rluc-H285TAG mRNA was coinjected into zebrafish embryos with PylT^**NPOMH**^.^108^ Following 1 min of irradiation at 5 h post fertilization (hpf), Rluc showed a roughly 6-fold increase in activity when compared to nonirradiated samples (Figure 35B). The increase in Rluc activity following **NPOMH** decaging is roughly double the change in activity following **ONBH** decaging in the same reporter.^89^ Also, as was observed with Rluc-H285**ONBH**, there was notable background activity of the nonirradiated, caged Rluc-H285**NPOMH** when compared to the control without ncAA, likely due to incomplete deactivation of Rluc when H285 is caged.^108^

As a second application of **NPOMH** for light control of protein activity in zebrafish, an active site histidine (H289) within Cre recombinase was mutated.^108^ H289 in Cre recombinase has been shown to be unable to accommodate bulky aromatic residues, making it an attractive choice for **NPOMH** introduction to demonstrate the utility of the ncAA to block protein activity due to steric hindrance imposed on an active site.^182^ When active, Cre recombinase excises the EGFP and stop codon from a Cre-stoplight reporter, resulting in exclusive expression of mCherry (discussed in more detail in section 3, Figure 5A). Nonirradiated samples of Cre recombinase-H289**NPOMH** showed high green fluorescence and no red florescence, as expected.^108^ After irradiation with 405 nm light at 5 hpf, mCherry fluorescence and some retained green florescence (due to EGFP production prior to irradiation) was observed, indicating successful off-to-on switching of DNA recombination.

Proteases are an attractive target for histidine caging as many rely on histidine as a general base, including serine proteases like hepatitis C virus protease (HCVp).^407^ HCVp was placed under optical control *in vivo* by replacing the active site histidine (H57) with **NPOMH** (Figure 35C) in zebrafish embryos.^108^ HCVp-H57 is involved in a catalytic triad with D81 and S139, where D81 forms a hydrogen bond with H57, which in turn deprotonates S139 for nucleophilic attack of the protein substrate.^408^ To measure the activity of HCVp-H57**NPOMH**, a fluorescent membrane translocation reporter bearing an HCVp-specific cut site between the membrane and mCherry was used.^108^ Active HCVp results in homogeneous distribution of mCherry florescence in cells. At 24 hpf, expression of the reporter peaked, and embryos were irradiated with 405 nm light followed by a 3-h incubation. Embryo brain and eye cells were imaged, and the cytoplasm-to-membrane (C/M) ratio was determined. As seen in Figure 35D,E, prior to light exposure there was no HCVp activity, and after irradiation near complete protein activity was recovered.

The NPOM caging group showed significantly improved decaging for histidine in contrast to ONB. However, because selections have not yet yielded a synthetase specific for **NPOMH**, chemical tRNA synthesis was instead used to encode this ncAA. This represented the first application of a chemically acylated tRNA for genetic code expansion in a multicellular model organism, the zebrafish embryo. Chemically acylated tRNAs, however, are not as easily delivered into mammalian or bacterial cells.^409,410^ Photocaged histidines have been used to control protein activity by masking hydrogen bonding capabilities as well as acid–base chemistry. There are additional avenues of controlling histidine function that have not been explored, such as histidine phosphorylation and histidine metal chelation.^411,412^

## 8 Glutamate/Aspartate

**Figure 36:**
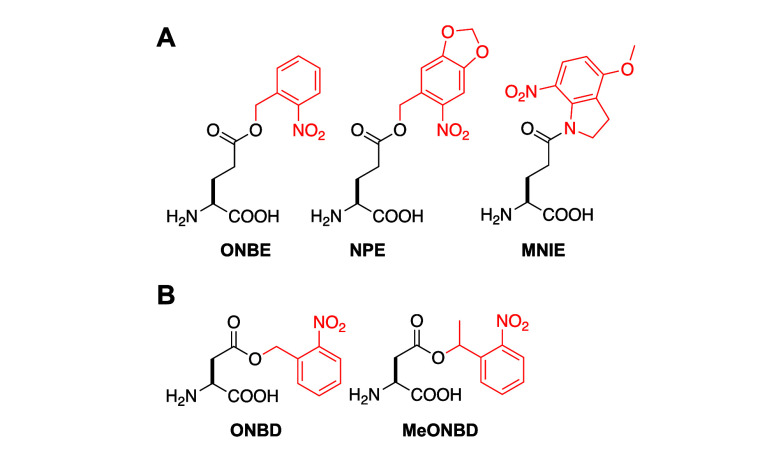
Structures of genetically encoded photocaged (A) glutamate and (B) aspartate with caging groups indicated in red.

Aspartic and glutamic acid are critical residues in many enzymes, as their negatively charged side chains play key roles in proton transfer and substrate binding through cation stabilization.^99,413^ Interestingly, the genetic incorporation of photocaged glutamate and aspartate for optical control of enzyme function was not achieved until 2023. One approach utilized the *o*-nitrobenzyl and nitropiperonyl caging groups to synthesize the photocaged glutamates **ONBE** and **NPE**, respectively (Figure 36A).^99^ In another approach, commercially available 4-methoxy-7-nitroindolinyl caged glutamate (**MNIE**, Figure 36A), which was adopted from neurochemical applications of the same light-activated glutamate, was genetically encoded.^100^ The nitroindoline moiety of **MNIE** is known for its two-photon photolysis mechanism for improved spatiotemporal activation and is preferred for the caging of carboxylic acids due to its stability toward hydrolysis.^26,414,415^ Two different caging groups were installed on aspartate, *o*-nitrobenzyl (**ONBD**) and methyl-*o*-nitrobenzyl (**MeONBD**, Figure 36B),^101^ which might be susceptible to esterases in the same way as **ONBE** and **NPE**.^416^

To encode **ONBE** and **NPE**, five active site residues of *Mb*PylRS were randomized and subjected to a double-sieve selection.^99^ An efficient synthetase for **ONBE** was identified through this process (ONBERS, Table 1), but no hits were identified for **NPE**. Luckily, when ONBERS was tested with **NPE**, ncAA-dependent expression was observed, yielding a synthetase with excellent selectivity for **ONBE** or **NPE** over endogenous amino acids. ONBERS selectivity was validated through mass spectrometric analysis of sfGFP-Y151ncAA expressed in bacteria and EGFP-Y39ncAA expressed in mammalian cells, where negligible detection of canonical glutamic acid was observed.

**Figure 37:**
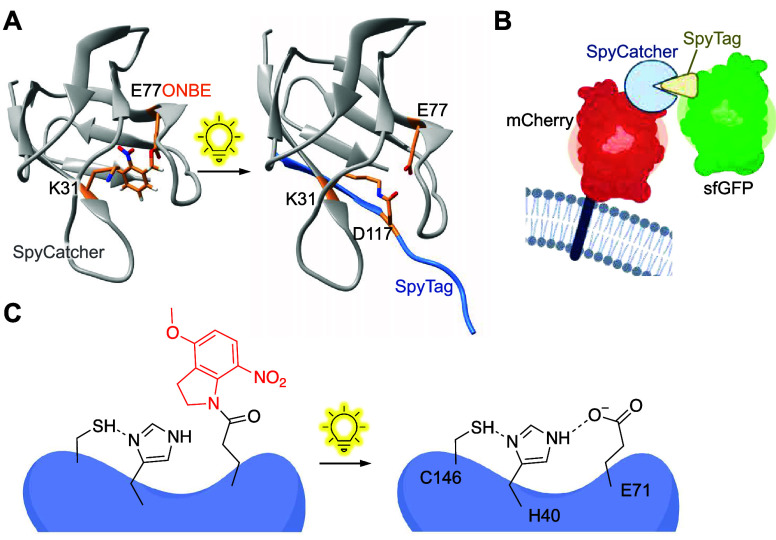
Applications of caged glutamates. (A) Optical control of SpyCatcher binding to SpyTag via mutation of the key residue E77 to **ONBE** (PDB 4MLI, with **ONBE** generated in UCSF Chimera). (B) Photoactivatable SpyCatcher fused to membrane-bound mCherry was selectively labeled by sfGFP-SpyTag only after **ONBE** decaging. (C) Incorporation of **MNIE** into the catalytic triad of HRV3C for optical control of protease activity.^99^

Further, **ONBE** and **NPE** were applied for the optical control of SpyCatcher binding to SpyTag. The SpyCatcher-SpyTag system allows for covalent protein conjugation through isopeptide bond formation between K31 of SpyCatcher and D117 of SpyTag, catalyzed by E77 of SpyCatcher (see Figure 14A in section 3).^417^ In this version of a photoactivatable SpyCatcher, the essential glutamate E77 was replaced with **ONBE** or **NPE** (Figure 37A) for complete inhibition of covalent modification. Irradiation of recombinant caged-SpyCatcher with 365 nm light for an extended period of 60 min yielded ∼60% active SpyCatcher, as measured via gel shift assay after treatment with SpyTag. Further, a membrane-bound mCherry-photoactivatable-SpyCatcher fusion was used for fluorescent labeling with SpyTag-sfGFP (Figure 37B). In the absence of light, only red fluorescence was detected at the cell membrane. UV irradiation (30–60 min) yielded the active SpyCatcher for localization of SpyTag-sfGFP to the cell surface. Despite unusually long irradiation times, the precise and light-controlled fusion of two proteins in mammalian cells was achieved. A photoactivated SpyCatcher was also generated by caging K31 (see section 3, Figure 14) and in a complementary approach, the carboxy group could possibly be substituted with a caged aspartate (see below).

The synthetase encoding **MNIE**, MNIERS (Table 1), was evolved using the *Ec*LeuRS/tRNA system.^100^ Four positions within the LeuRS binding pocket were randomized and subjected to positive and negative selection rounds using URA3 and HIS3 reporters in yeast auxotrophs.^418^ Incorporation of **MNIE** into EGFP-Y39TAG showed minimal background from incorporation of endogenous amino acids in bacterial cells expressing the MNIERS/LeuT pair, and efficient decaging with 5 min of 365 nm irradiation was observed by mass spectrometry.

**MNIE** was further applied for the optical control of enzyme function by incorporation into the catalytic triad (H40-E71-C146) of human rhinovirus 3C protease (HRV3C protease, Figure 37C). A catalytic triad hydrolyzes or transfers a substrate and requires 1) a nucleophile (C, S, or T), 2) a general base (H, K, or S), and 3) a general acid (H, E, or D). In the HRV3C protease triad, the negative charge of E71 is key for the orientation of H40 in the transition state.^413,419^ Activity of photoactivatable HRV3C protease was measured through fusion to a self-assembling split-GFP (Flip-GFP) separated by an HRV3C protease-cleavable linker. In the absence of light, the steric encumbrance and neutral charge of **MNIE** in the catalytic triad was sufficient to block protease activity. Irradiation with 365 nm light for 10–30 s converted **MNIE** to glutamate and activated 50% of HRV3C.^100^ This represents another intriguing solution to the generation of light-activated proteases, in addition to the use of caged tyrosine, caged cysteine and caged histidine discussed in sections 4, 5, and 7, respectively.

Two photocaged aspartates have been genetically encoded for optical control of protein function, **ONBD** and **MeONBD** (Figure 36B). The additional methyl group of **MeONBD** improved quantum yield,^26^ thus improving decaging efficiency, with complete photolysis occurring after only 1 min of UV irradiation, instead of 5 min for **ONBD**.

For genetic incorporation of **ONBD** and **MeONBD**, computational analysis of **ONBD** binding by *Mb*PylRS determined four residues to be involved in **ONBD** recognition. A synthetase known for encoding aspartic acid benzyl ester (BnDRS) was then randomized at those four positions to create a library of enzymes that were passed through positive selections with **ONBD** and **MeONBD**. The resultant synthetase, ONBDRS (Table 1), was highly selective for both **ONBD** and **MeONBD** over endogenous amino acids when coexpressed with TAG-containing fluorescent reporters in both bacterial and mammalian cells.^101^

**Figure 38:**
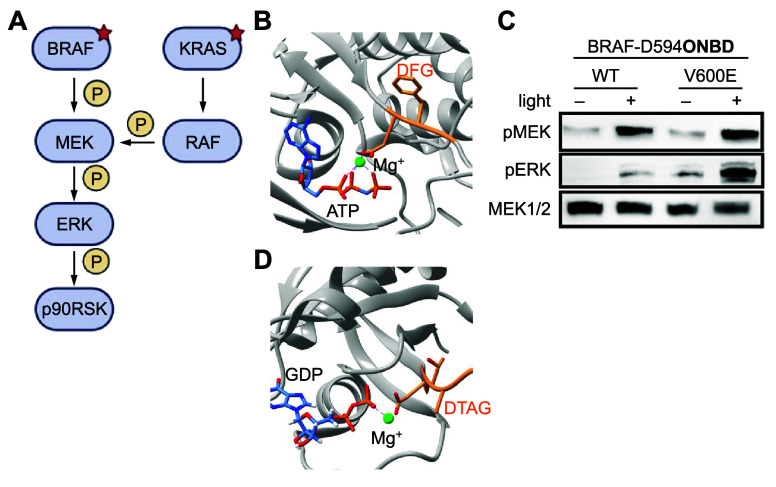
Photocontrol over the Ras/MAPK pathway using photocaged aspartate. (A) Signaling pathway of BRAF and KRAS where the red star indicates a photocaged aspartate. (B) Crystal structure of the DFG motif in BRAF (PDB 6U2G). (C) Optical activation of BRAF signaling for both wild-type BRAF-D594**ONBD** and the V600E oncogenic mutant. Reproduced with permission from ref (101). Copyright 2023 American Chemical Society. (D) Crystal structure of the DTAG motif in KRAS (PDB 4LDJ).

Light-activated aspartate was then used to engineer optical control of cell signaling. Two enzymes of the Ras/MAPK pathway (Figure 38A), the kinase BRAF (Figure 38B) and the GTPase KRAS (Figure 38D), have key aspartate residues in their highly conserved, activity-regulating DFG and DTAG motifs, respectively. In both cases, aspartate coordinates magnesium, which is essential for respective GTP and ATP binding and the regulation of enzyme activity.^420,421^ Through caging of these aspartates, downstream target phosphorylation was controlled with excellent temporal resolution, as MEK, ERK (Figure 38C), and 90RSK phosphorylation were absent until irradiation.

Further comparison of wild-type versus oncogenic mutants of BRAF and KRAS was enabled through acute optical activation. Oncogenic BRAF-V600E resulted in increased levels of pERK relative to wild-type (Figure 38C), matching previous reports.^422^ In the side-by-side comparison of multiple oncogenic mutants (G12C, G12V, G12D, and G13D) of KRAS-D57**MeONBD**, each showed faster phosphorylation cascades than that of wild-type KRAS-D57**MeONBD**. Additionally, the G13D mutant, which is less frequent than the G12 mutants in cancer patients, showed the highest level of downstream phosphorylation in this study.^423^ Modulating enzyme activity by optically controlling magnesium binding may be broadly applicable to other metal binding aspartates. Another GTPase, NRAS, has also been optically controlled in mammalian cells and zebrafish embryos through caging of K16 with **ACK** (section 3, Figure 9C–D).^70^

**Figure 39:**
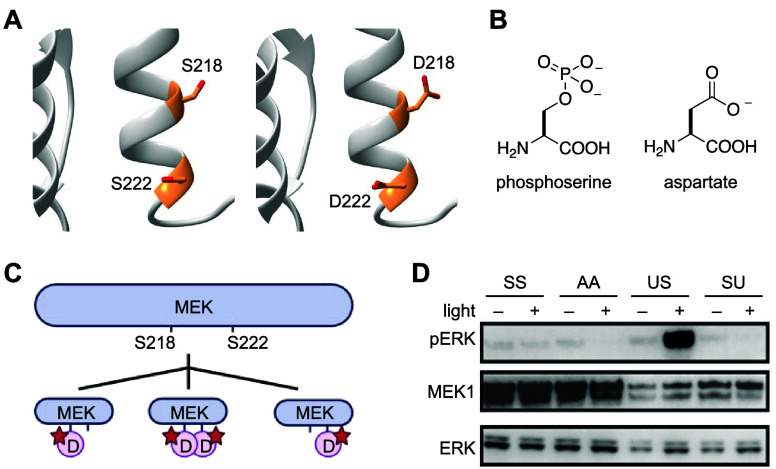
Photocaged aspartate acts as a phosphorylation mimic when decaged. (A) Crystal structure of MEK1-S218-S222 (left; PDB 7F2X) versus MEK1-S218D-S222D (right). (B) Structural similarity of phosphoserine and aspartate. (C) Mimicry of MEK phosphorylation at positions S218, S222, or both through incorporation of ONBD to investigate the dual phosphorylation mechanism of MEK. (D) Western blot results of different MEK1 mutants on downstream ERK phosphorylation (SS = wild-type; AA = S218A/S222A; US = S218**ONDB**; SU = S222**ONDB**). Reproduced with permission from ref (101). Copyright 2023 American Chemical Society.

In addition to upstream regulation of the Ras/MAPK pathway, photocaged aspartate was utilized for its structural and electronic similarities to phosphorylated serine/threonine after decaging (Figure 39B),^424^ with the goal of further elucidating the dual phosphorylation mechanism of MEK. Native signal transduction from RAF yields phosphorylation at S218 and S222 of MEK1 (Figure 39A). The double aspartate mutation (S218D and S222D) mimics MEK1 phosphorylation and produces a constitutively active MEK1.^425^ Through incorporation of the caged aspartates **ONBD** or **MeONBD** at either or both S218 and S222, time-resolved control of “phosphorylation” at each position was possible, enabling comparison of its effect on MEK1 activity (Figure 39C). Decaging of MEK1-S218**ONBD** yielded substantial ERK phosphorylation, while decaging of MEK1-S222**ONBD** yielded negligible activity comparable to both the wild-type and double alanine (S218A/S222A) negative control mutants (Figure 39D). In combination with the known requirement of dual phosphorylation for MEK1 activation,^426^ these results suggest that phosphorylation at S218 may induce phosphorylation at S222.

At the time of this review, optical control of protein function using photocaged glutamate and aspartate was just recently reported. As additional caging groups are explored for these two amino acids, the decaging efficiencies may be improved for fast activation in increasingly complex environments. With the prevalence of glutamate and aspartate in catalytic triads and their roles in cation coordination, protein solubility, and stabilizing functions like salt bridges and hydrogen bonding, these photocaged ncAAs are expected to be important tools for the future study of a wide range of protein functions with spatial and temporal precision.

## 9 Phenylalanine

**Figure 40:**
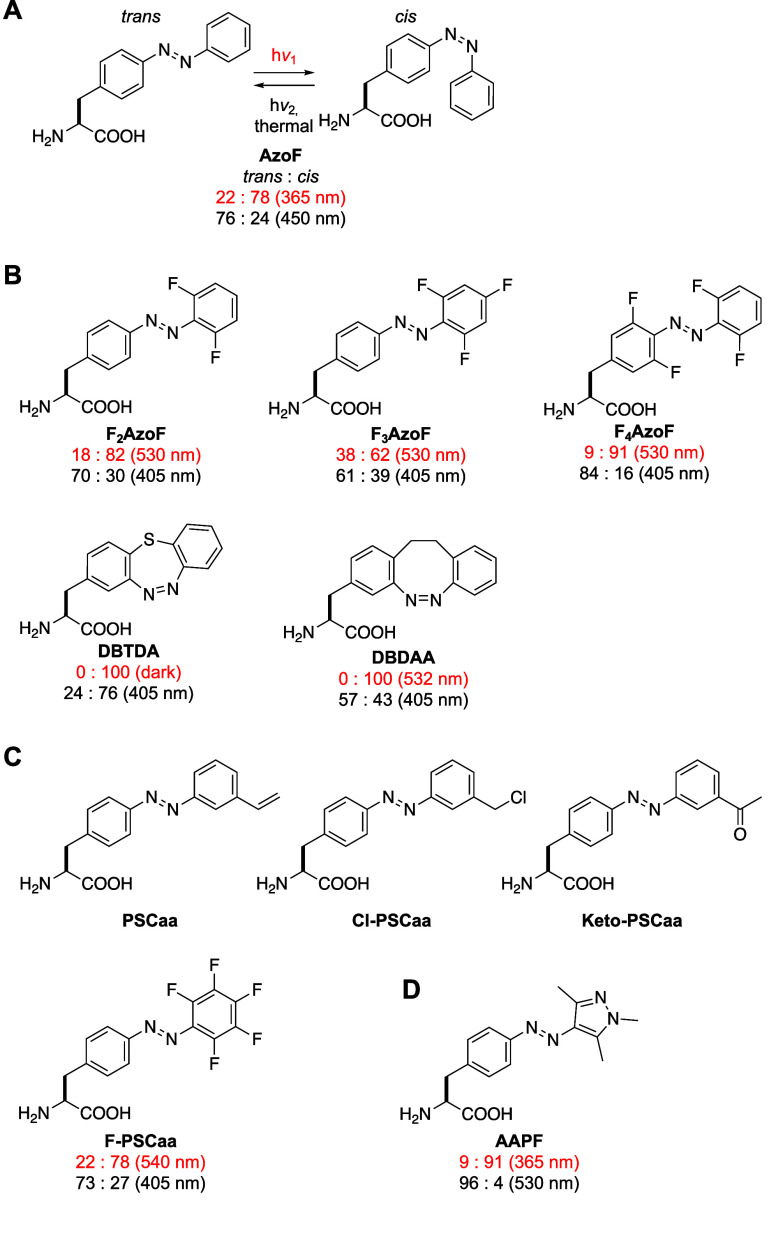
Genetically encoded photoswitchable phenylalanine derivatives. (A) Reversible isomerization of **AzoF** to its *cis* and *trans* isomers using two distinct wavelengths. (B) Changes to the structure of **AzoF** for improved photophysical properties. (C) Photoswitchable ncAAs bearing additional cross-linking capabilities. (D) Azo-heterocycle ncAAs. Experimentally determined PSS values listed below each ncAA as the ratio of *trans* to *cis* isomers after irradiation with h*v*_1_ (red) or h*v*_2_, with wavelengths or thermal relaxation specified in parentheses.

Most optically controlled ncAAs contain a photolabile caging group which exclusively allows for a one-directional light response, typically as an irreversible on-switch upon decaging. While photodecaging is broadly applicable to optically control protein function, the reversible control provided via photoswitchable ncAAs offers multiple advantages. Similar to the reversible nature provided by optogenetics,^427^ photoswitchable ncAAs benefit from 1) improved spatial control as activated proteins revert back to their inactive dark state when diffusing away from the light beam, 2) pulsed enzymatic function triggering systems in a nonsustained, transient fashion, and 3) mimicry of native biological processes, which are often reversible. The chemistry of photoswitchable ncAAs also benefits from not producing byproducts upon irradiation and shows greater tunability for activating wavelengths.^428^ In order to provide reversible off-to-on and on-to-off switching of protein function, azobenzene-containing amino acids, or azo-ncAAs, such as azobenzene phenylalanine (**AzoF**, Figure 40A) have been developed and genetically encoded.^90^

Azobenzenes undergo characteristic switching between *cis* and *trans* isomers (Figure 40A), which each have distinct geometries and dipole moments.^429^ Azobenzenes have two absorbance maxima, with a strong band representing the π–π* transition and a red-shifted band representing the n-π* transition.^429^ Irradiation with the wavelength corresponding to the π–π* transition induces rotation about the N = N bond to form the *cis*-isomer, while either irradiation with the longer wavelength corresponding to the n-π* transition or thermal relaxation yields reversion back to the *trans*-isomer.^429,430^ Sufficient irradiation leads to the establishment of a photostationary state (PSS). Ideally, the PSS of an azo-ncAA would reach 100% of the specified isomer with irradiation at nondamaging, tissue-permeable wavelengths.^429,431^ The thermal stability of the higher energy isomer is important, and different stabilities can be desired depending on the goal of an experiment. For applications in which the high energy isomer must persist but has a short thermal half-life, for example, prolonged irradiation would be necessary. Additionally, azobenzenes have been extensively applied in photopharmacology, providing optical control of drug activity.^3,10,11^ Through direct incorporation of the azobenzene functionality onto proteins via genetic code expansion, reversible optical control of protein function has been studied.

Depending on the intended effect of irradiation on protein activity, namely photoactivation or photodeactivation, the photochemical properties of azo-ncAAs are necessary to consider. When utilizing photoswitchable ncAAs for photodeactivation, the biologically inactive isomer should be achievable in high yields to avoid background activity. Conversely, near complete photoswitching is less important in photoactivation applications if the lower energy (dark state) isomer is biologically inactive.^428^ The original **AzoF** achieved switching to 78% *cis* after 365 nm irradiation, meaning any protein-based optical OFF-switch with AzoF would be limited to that PSS.^90,432^ To improve the photophysical properties, including both PSS and thermal stability, of azobenzene chromophores and azo-ncAAs, *ortho* substituents such as fluorine, chlorine, or other electron withdrawing groups can be added. To red-shift the n-π* absorption of the original **AzoF** scaffold and increase the half-life of the *cis*-isomer, *ortho*-fluorination was employed (**F**_**2**_**AzoF**, **F**_**4**_**AzoF**, Figure 40B).^92,431^ Fluorine substituents further benefit from their small size, making them amenable to synthetase evolution.^91^

To selectively encode **AzoF**, six residues in the tyrosine binding site of *Mj*TyrRS were randomized and the resulting library was subjected to three positive and two negative rounds of selection.^90^ The resultant synthetase, termed AzoFRS (Table 1), has been used to encode **AzoF** into a catabolite activator protein^90^ and sfGFP,^432^ both expressed in *E. coli*. Concurrent with the design of newer **AzoF** analogues, additional synthetases were also identified for these ncAAs, the engineering of which is discussed below.

**Figure 41:**
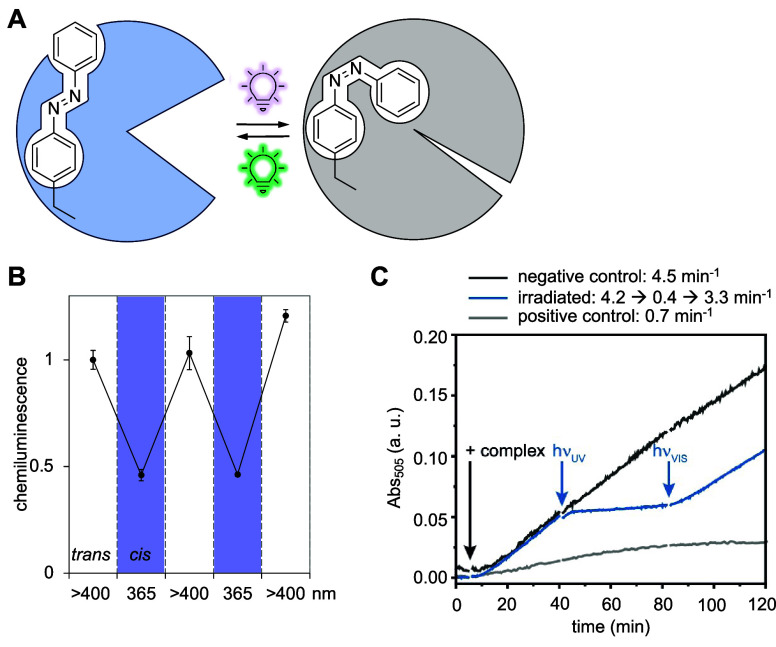
Light regulation for allosteric control of enzymatic function. (A) Cartoon depiction of allosterically placed azo-ncAAs in which isomerization converts the active *trans* state to the inactive *cis*. Reproduced with permission from ref (433). Copyright 2019 Elsevier Ltd. (B) Reversibility of the activity of firefly luciferase bearing **F**_**4**_**AzoF** using either 405 or 365 nm irradiations. Reproduced with permission from ref (91). Copyright 2018 Wiley-VCH Verlag GmbH & Co. KGaA, Weinheim. (C) Photoswitching of ImGPS activity. The negative control (black line) is nonirradiated HisF-**AzoF**(*trans*), the positive control (light gray line) is preirradiated HisF-**AzoF**, and the blue line demonstrates reversible activation of HisH complexation through isomerization of **AzoF**. Reproduced with permission from ref (154). Copyright 2019 Elsevier B.V.

As discussed in previous sections, optical control of enzyme function is most commonly achieved through installation of caging groups at active site residues. Interestingly, only a few enzymes have been optically controlled through genetic incorporation of photoresponsive amino acids at allosteric positions (Figure 41A).^433^ As allosteric enzyme regulation is prevalent in biology, minimally invasive tools for probing enzyme allostery are important. In addition to the optical control of allostery demonstrated by caging tyrosine in tryptophan synthetase (see section 4), photocontrolled enzyme allostery was also achieved with azo-ncAA encoding in mammalian cells.

Reversible regulation of the chemiluminescence of firefly luciferase (Fluc) was accomplished via the introduction of **AzoF**, **F**_**2**_**AzoF**, and **F**_**4**_**AzoF** (Figure 41B) in mammalian cells.^91^ A mammalian optimized synthetase selective for these azo-ncAA analogues, AzoFRS2 (Table 1,) was identified by screening a library of *Mb*PylRS mutants with random mutations at L270, Y271, L274, N311, C313, and Y349 with a fluorescent reporter.^91^ Reversible allosteric control of Fluc seeks to destabilize the protein when the ncAA is in one configuration such that the necessary protein conformational changes for the bioluminescence reaction are inhibited. Photoswitching then allows the protein to regain activity. Experimental and computational validation demonstrated decreased favorability for the d-luciferyl-AMP substrate when Fluc-W417**AzoF** was in its *cis* configuration and improved bioluminescence when in its *trans* configuration. Further, as observed with azobenzene chromophores,^434,435^ the introduction of two or four *ortho*-fluorines to the azo-ncAA provided a red-shift of the wavelength necessary for *trans*-to-*cis* isomerization and improved its overall PSS and thermostability (Figure 40B).

The third application of optically controlled allostery utilized the bienzyme complex of imidazole glycerol phosphate synthase (ImGPS).^154^ In a bacterial system, both photocleavable (see section 4) and photoswitchable ncAAs were encoded into HisF, the cyclase subunit of ImGPS which allosterically activates the glutaminase HisH. Three HisF regions, namely loop 1, the ammonia channel, and the hinge region, were selected for ncAA incorporation based on their known roles in HisF allostery and distance from the HisH active site. Following characterization of HisF-**AzoF** mutants for stability, structure, and function, light regulation of enzymatic function was explored. When **AzoF** was in its *trans* configuration, the HisF-**AzoF** enzyme complex was active. Irradiation and subsequent isomerization of **AzoF** to its *cis* geometry prevented HisF from recognizing its substrate, thus blocking enzymatic function. Finally, irradiation with visible light reverted **AzoF** back to the *trans* state for reversible control of the enzyme function with light (Figure 41C). Further analysis of HisF-**AzoF** with molecular dynamic studies revealed the importance of HisH residue H178 for glutaminase activity, challenging a previously accepted mechanism of action.

While both approaches relied on crystal structures for computational predictions of allosteric control, thus limiting the scope of available targets, two applications of genetically encoded azo-ncAAs have provided reversible optical control of enzyme allostery for different systems.

**Figure 42:**
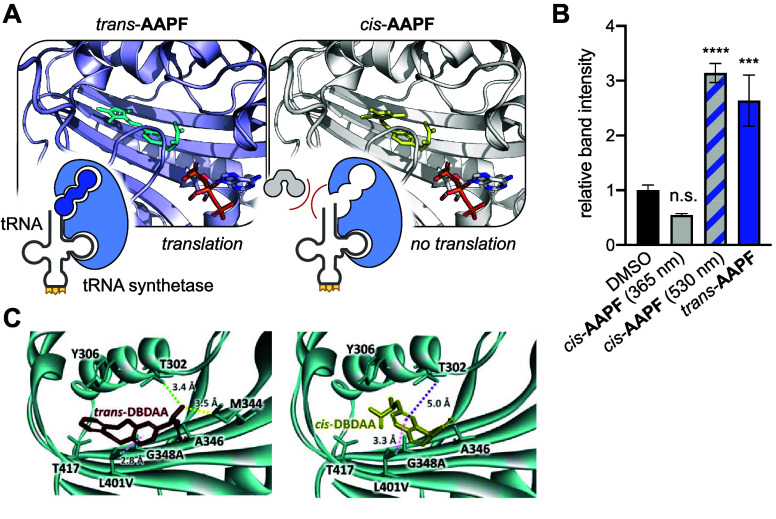
Optical control of translation through genetic code expansion. (A) Computational rendering of *trans*- versus *cis*-**AAPF** recognition by AzoFRS2.^97^ (B) Western blot quantification of sfGFP-Y151**AAPF** irradiated with either 365 nm, 365 nm then 530 nm (*trans*), or solely 530 nm (*trans*) light. Panels (A,B) are reproduced with permission from ref (97). Copyright 2023 American Chemical Society. (C) Simulated *trans*-DBDAARS active site binding either *trans* (red) or *cis***DBDAA**. Reproduced with permission from ref (94). Copyright 2023 Royal Society of Chemistry.

Another application of photoswitchable ncAAs was toward optical control of translation using two unique analogues of **AzoF**, arylazopyrazole phenylalanine (**AAPF**) and dibenzo[*c,g*][1,2]diazocine alanine (**DBDAA**). The first instance of a photoswitchable ncAA bearing an azo-heterocycle functionality (**AAPF**), specifically arylazopyrazole (Figure 40D), demonstrated an improved PSS and longer thermal stability (91% *cis* at 365 nm, 96% *trans* at 530 nm, *t*_1/2_ ∼ 36 h) compared to **AzoF** (78% *cis* at 365 nm, 76% *trans* at 450 nm, *t*_1/2_ ∼ 13 h).^435,436^ Through screening of previously utilized tRNA synthetases, **AAPF** was found to be encoded by AzoFRS2^91^ (Table 1) with only minor background incorporation of canonical amino acids. Interestingly, in an attempt to encode *cis*-**AAPF**, selectivity for *trans*-**AAPF** was observed.^97^ As supported by computational analysis of AzoFRS2 binding energies to *cis* versus *trans*-**AAPF** (Figure 42A), the experimental bacterial expression of sfGFP-Y151**AAPF** was not possible when cells were persistently irradiated with 365 nm light, as *cis*-**AAPF** was maintained and the isomer was not a substrate for the synthetase. However, isomerization from *cis* back to *trans*-**AAPF** yielded sfGFP production (Figure 42B). While this provided a unique approach for the optical control of translation, the poor stability of *cis*-**AAPF** in media containing glutathione (known to reduce azobenzenes and force *cis* to *trans* isomerization^437^) limits its applicability for the optical control of translation in live cells.

Another azo-ncAA approach for the optical control of translation utilized dibenzodiazocine (**DBDAA**, Figure 40B), an eight-membered cyclic chromophore with a rigid structure such that the ground state is the *cis* isomer.^94^ To selectively encode *cis*-**DBDAA**, a semi-rationally designed pool of synthetases was subjected to three rounds of selection, identifying *cis-*DBDAARS (Table 1).^94^ Notably, irradiation of *cis*-**DBDAA** with 405 nm light produced *trans*-**DBDAA** and decreased expressed protein yields by 48%. Synthetase screening for *trans*-**DBDAA** identified a unique *Mm*PylRS mutant, *trans-*DBDAARS, with improved specificity for the *trans* isomer. Molecular modeling depicted a planarized binding cavity in *trans-*DBDAARS, which better fit the crown structure of *trans*-**DBDAA** (Figure 42C); however, some synthetase leakiness was observed, as protein was expressed in both no ncAA and *cis*-**DBDAA** conditions. The encoding of *trans*-**DBDAA** was further confirmed by the photoclick labeling with sydnone-Cy3 for validation via in-gel fluorescence. Through evolution of two distinct synthetases specific to either the *cis* or *trans* isomers of **DBDAA**, optical control of genetic code expansion was achieved.^94^

Similar to the ground-state *cis* isomer in the case of **DBDAA**, a dibenzothiadiazepine chromophore was employed in the photoswitchable **DBDTA** (Figure 40B).^93^ The dibenzothiadiazepine of **DBDTA** acts as a dipolarophile for cycloaddition with nitrile imines formed from the photolysis of diarylsydnone (DASyd) for photoclick labeling of proteins containing **DBDTA**. Further, isomerization of **DBDTA** to its *trans* isomer increases its ring strain and accelerates *in situ* labeling. To encode **DBDTA**, DBTDARS (Table 1) was evolved from a library of *Mm*PylRS mutants rationally designed from the synthetase for *p*-benzoyl-phenylalanine. Bacterial expression of sfGFP bearing **DBTDA** at three sites (K3, N149, and Q204) allowed for subsequent photoclick chemistry between **DBTDA** and the DASyd-Cy3 for in-gel and live cell fluorescent labeling.

In addition to optical control of enzyme allostery and translation, photoswitchable ncAAs have also been genetically encoded for applications in light-responsive materials.^438^ Elastin-like proteins (ELPs) are a class of protein-based biomaterials bearing a repeating VPGXG motif, which can be functionalized at X with a desired natural or noncanonical amino acid.^439^ ELPs undergo a reversible, soluble-to-insoluble phase transition at their lower critical transition temperature (Tt), which is dependent on ELP composition and hydrophobicity.^439−441^ The distinct dipole moments and geometries of *cis* and *trans* azo-ncAAs presented a unique approach for optical control of ELP turbidity.^98,438^ Additionally, the repeating nature of ELPs presents the opportunity for multiple instances of ncAA placement and thus an amplified response to optical switching; however, it also requires a system for efficient multisite genetic code expansion, which is nontrivial.^40,55,442^ The efficient encoding of 3–30 instances of a ncAA per protein was made possible through genome-wide recoding of an *E. coli* strain to remove all native UAG codons and the associated release factor, RF-1.^55^

**Figure 43:**
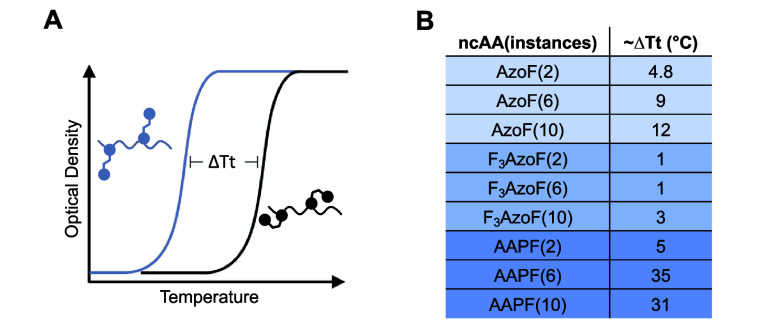
Optical control of elastin-like polypeptides with azo-ncAAs. (A) Schematic of the effect of azobenzene isomerization on the turbidity of ELPs bearing two or more instances of **AzoF**, **F**_**3**_**AzoF**, or **AAPF**. Reproduced with permission from ref (438). Copyright 2021 John Wiley & Sons, Inc. (B) Effects of structure and increasing instances of azo-ncAAs on the turbidity profile of ELPs, as represented by the differences in lower critical transition temperature (Δ*T*t).^98,438^

To characterize the effect of multiple hydrophobic azo-ncAAs on the Tt of ELPs, a hydrophilic ELP bearing alternating glycine and alanine residues at the X position was selected. Four ELP variants, each with 60 pentapeptides, were designed with either 0, 2, 6, or 10 TAG codons and expressed with either **AzoF**, **F**_**3**_**AzoF**, **F**_**4**_**AzoF**, or **AAPF** (Figure 40**A, B, and D**).^98,438^ The effect of isomerization on ELP turbidity profiles was then determined (Figure 43A). Switching to the more hydrophilic *cis* isomer (365 nm) led to a higher Tt than that of ELPs bearing the more hydrophobic *trans* isomer (405 nm), with 10 instances of AAPF demonstrating the largest Δ*T*t when applied to a glycine-modified ELP (Figure 43B).^98^ The increased presence of multiple ncAAs correlated to a larger shift in Δ*T*t, demonstrating the amplified effect of photoisomerization on the solubility of the ELP. Optical control of ELPs provides a promising springboard for the development of novel, tunable, and customizable light-responsive biomaterials.

**Figure 44:**
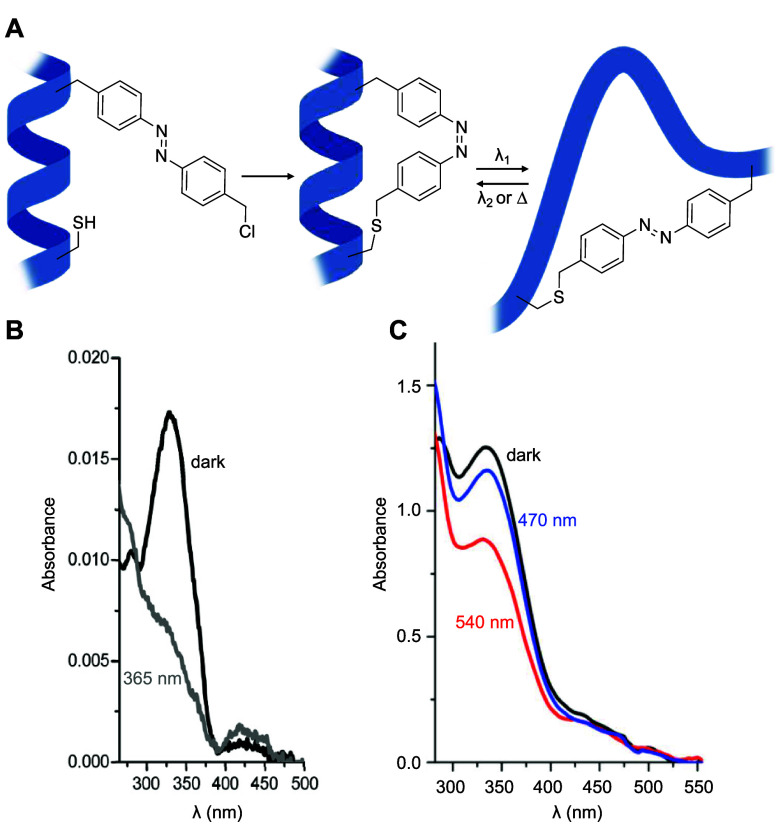
Genetically encoded peptide staple. (A) **Cl-PSCaa** reacting with cysteine via S_N_2 reaction. Irradiation of the stapled protein melts the α helix. UV–vis spectra of CaM stapled by (B) **Cl-PSCaa** or (C) **F-PSCaa** before and after irradiation. Panel (B) reproduced with permission from ref (95). Copyright 2014 Wiley-VCH Verlag GmbH & Co. KGaA, Weinheim. Panel (C) reproduced with permission from ref (96). Copyright 2015 American Chemical Society.

Azo-ncAAs were further modified to include reactive handles for stapling to cysteine such that a covalent, photoswitchable bridge is formed in the protein of interest for an amplified effect on conformational changes after irradiation (Figure 40C).^95,96^ Cross-linking azobenzenes have been used extensively for the optical control of synthetic peptides, providing noninvasive structure manipulation for studies of function.^443−445^ Symmetric, bifunctional cross-linkers require strategically placed cysteines for nucleophilic attack onto the ligand, such that isomerization of the azo-bridge induces a large change in helical content, dependent on the positioning of the two cysteines.^446−448^ The applicability of bifunctional cross-linkers can be limited by poor water solubility and diminished impact on protein conformation.^448^ Additionally, bifunctional azobenzenes cannot staple peptides in cells due to the high and promiscuous reactivity of their electrophilic groups,^449,450^ an issue that has been elegantly addressed through genetic encoding of thiol-reactive azo-ncAAs (Figure 44A). This enables the use of a less reactive electrophile due to the proximity of the cross-linking cysteine and minimizes the flexibility of the staple, as fewer rotational bonds are present, thus magnifying the effect of isomerization and expanding photoswitchable control into live cell systems. To date, four cross-linking azo-ncAAs, also called photoswitchable click amino acids or PSCaas, have been reported: **PSCaa** containing a reactive vinyl, the ketone **Keto-PSCaa**, the benzyl chloride **Cl-PSCaa**, and the pentafluorobenzene **F-PSCaa** (Figure 40C). This approach has been utilized to demonstrate optical control of protein folding in live cells.^95,96^

As the added reactive handles necessary for cysteine reactivity elongated the structure of each ncAA, new synthetases needed to be evolved. Using the *o*-methyl-tyrosine synthetase from *M. mazei* as a guide, residues that interacted with **PSCaa** were computationally determined and semi-rationally randomized to build a library with 4 × 10^7^ mutants. The library was passed through negative and positive rounds of selection to yield PSCaaRS, which encoded **PSCaa**, **Cl-PSCaa**, and **F-PSCaa**, and Keto-PSCaaRS, which encoded **Keto-PSCaa** (Table 1).^95^ The fidelity and specificity of these synthetases was confirmed via bacterial expression of myoglobin and mammalian expression of GFP. Further, **PSCaa** encoded with PSCaaRS was investigated as a photoswitchable ncAA in the NMDA receptor in mammalian cells, but it was not used for stapling with cysteine.^375^ A conserved proline residue (P532) in the agonist-binding domain of GluN1 was mutated to **PSCaa** to control receptor activity at the interface with GluN2.^375^

As PSCaaRS was also functional for **Cl-PSCaa**, the chlorinated ncAA was genetically incorporated into the α helix of calmodulin (CaM), a small protein with a well-characterized helical structure that expresses well in *E. coli*.^95^ CaM was further modified with a cysteine at the i+7 position, allowing *in situ* azo-bridge formation by **Cl-PSCaa** (Figure 44B), which was quantitative as measured by mass spectrometry. While the PSS and thermal stability values of **PSCaa**, **Keto-PSCaa**, and **Cl-PSCaa** ncAAs alone were not reported, the absorbance of CaM stapled with **Cl-PSCaa** indicated efficient isomerization from the *trans* to the *cis* isomer upon irradiation with 365 nm light. However, no stapling was reported for **Keto-PSCaa**, nor was reverse isomerization from *cis* to *trans* of proteins stapled with **PSCaa** or **Cl-PSCaa**.

Further evolution of the photoswitchable and cross-linking azobenzene-ncAA provided additional insight on the applicability of reversible control of protein structure. The second generation **PSCaa** was a pentafluoro analogue of **AzoF**, **F-PSCaa** (Figure 40C), which undergoes nucleophilic aromatic substitution with cysteine.^96^ Reversible switching was demonstrated for **F-PSCaa** stapled CaM using successive irradiations with 540 and 470 nm light (Figure 44C). Additionally, the fewer number of rotational, single bonds in **F-PSCaa** produced a more rigid staple, which might be beneficial in the optical control of protein secondary structure.

Although not through direct incorporation of a photoswitchable ncAA, genetic code expansion and azobenzenes have been combined to achieve reversible optical control over kinase activity.^15^ A bioorthogonal ncAA, a class of ncAAs which have been reviewed elsewhere,^451−453^ was encoded into the kinase MEK1/2 for site-specific bioconjugation to a ligand that was linked to an inhibitor for MEK1/2 via an azobenzene derivative. Through irradiation of the photoswitchable inhibitor bound MEK, the activity of the protein was reversibly controlled, as demonstrated by a decrease in downstream phosphorylation. The bioorthogonal addition of a photoswitchable moiety to a protein of interest provides an interesting avenue for further exploration, as this strategic design can be used to develop either cis-ON or cis-OFF applications for a wide array of protein targets. Importantly, as enzyme specificity is conveyed through the bioconjugation reaction, promiscuous enzyme inhibitors can be used for this purpose.

Because of the differences in shape, size, and dipole moment of *cis* versus *trans* azobenzenes, the strategic placement of the photoswitchable chromophore into proteins has been used to directly impact protein folding through allosteric means. Photoswitchable ncAAs have also been used to optically control translation, utilizing the structural variation of *cis* and *trans* configurations of azo-ncAAs for differential synthetase recognition. Further, enhanced structure manipulation was achieved through isomerization of azo-bridges formed via ncAA-cysteine staples, amplifying the structural changes achieved through *cis/trans* isomerization. As photoswitchable ncAAs continue to evolve, there remains ample room for new applications. Azo-ncAAs have not yet been applied to sterically block active sites when in one configuration, nor have clickable azo-ncAAs been used to staple proteins *in cellulo*. The ability to genetically encode small biorthogonal handles for attachment of photoswitchable and photocleavable chromophores has generally been underexplored, and small conjugation handles might be particularly useful for this.^124,454,455^ The reversible control afforded with photoswitchable ncAAs provides high spatial accuracy and allows for flexible and repeated protein activation and deactivation to mimic the reversibility of many biological processes.

## 10 Miscellaneous

In addition to the photocaging of native amino acids and near-natural amino acids, entirely non-native amino acids have also been photocaged. These include 2,3-diaminopropionic acid (**DAP**) and citrulline (**CIT**).

**Figure 45:**
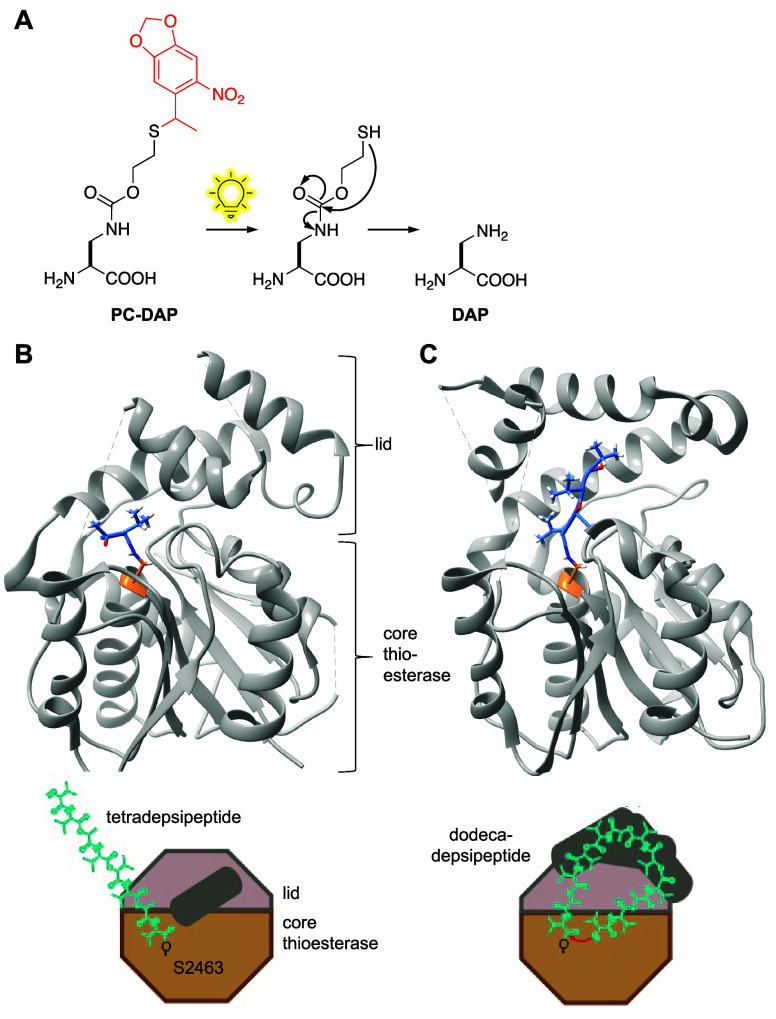
Valinomycin synthetase structures were solved using **DAP** trapping. (A) Structure of **PC-DAP**, which is photolyzed to the thiol intermediate for self-immolation to yield **DAP**. (B) The lid conformation of the synthetase remains mostly unchanged relative to wild-type apo-TE (not shown, PDB 6ECB) when the tetradepsipeptide is bound to the protein (PDB 6ECD).^102^ (C) A large conformational change occurs when the dodecadepsipeptide is in the active site, such that the lid sterically forces the depsipeptide to cyclize for valinomycin formation (PDB 6ECF). Panels (B,C) are reproduced with permission from ref (102). Copyright 2019 Springer Nature Limited.

Hydrolytic enzymes, such as proteases or esterases, containing active site serine or cysteine residues often utilize ester or thioester intermediates, respectively. As the half-lives of these intermediates are short (minutes to hours),^456^ the enzyme substrates cannot be captured for further analysis. In an effort to trap these intermediates, active site serine/cysteine residues were mutated to 2,3-diaminopropionic acid (**DAP**), yielding an amine side chain that reacts with the substrate of the enzyme to form an amide bond.^102^ In order to encode **DAP** into proteins via genetic code expansion, a protected analogue was required to provide a structural handle for synthetase engineering. A series of five protected **DAP** analogues was screened, with only one identified to reach sufficient incorporation concentrations in *E. coli*. The selected ncAA, termed here as photocaged-**DAP** or **PC-DAP**, was designed such that photolysis of the 6-nitropiperonal caging group yields a self-immolative thio-ethoxy intermediate which fragments to yield **DAP** (Figure 45A). An *Mb*PylRS synthetase specific for **PC-DAP** was evolved through five rounds of positive and negative selection (PC-DAPRS, Table 1) and proof of specificity was demonstrated by encoding **PC-DAP** into sfGFP in *E. coli.* Decaging of sfGFP-150**PC-DAP** was further confirmed by mass spectrometry.^102^

The trapping capacity of **DAP** was first demonstrated with the tobacco etch virus (TEV) protease by replacing the catalytically active cysteine residue with **DAP**, covalently trapping the substrate due to the inability of the enzyme to then hydrolyze an amide (in contrast to the natural thioester), thereby enabling pull-down and mass spectrometry analysis. In a second example, the function of the thioesterase domain of valinomycin synthetase was investigated. This enzyme oligomerizes tetradepsipeptides into a dodecadepsipeptide for cyclization and production of valinomycin, a potassium ionophore known for its antimicrobial and cytotoxic properties.^457^ As a secondary-metabolite-producing nonribosomal peptide synthetase, the catalytic cycle of valinomycin synthetase utilizes many acyl-enzyme intermediates that are too unstable for classical structure charaterization.^102^ To generate high-resolution structures of valinomycin synthetase intermediates, specifically in the thioesterase domain, the active site serine (S2463) was mutated to **DAP** to trap the first and last acyl-enzyme intermediates in this biosynthetic pathway. Successful trapping of these intermediates with **DAP** led to structure elucidation using X-ray diffraction (Figure 45B–C). The structure of the tetradepsipeptide intermediate closely matched that of the substrate-free thioesterase, where the “lid” region is highly ordered and does not interact with the tetradepsipeptide (Figure 45B). Upon elongation to the dodecadepsipeptide, the lid undergoes a significant conformational change (Figure 45C) such that the dodecadepsipeptide is forced back on itself, rather than extending out, which likely facilitates the necessary cyclization step to yield valinomycin. While the photocaging properties of **PC-DAP** conveying spatial and temporal control are not used here, this application of **PC-DAP** yielded key mechanistic insight into a previously illusive biosynthetic pathway. This approach provides an interesting adaptation of photocaged ncAAs and will hopefully be used to add temporal control to capturing other unique targets.

In addition to the several post-translational modifications mentioned in this review, citrullination is a noteworthy modification, wherein arginine is hydrolyzed to a neutral urea to produce citrulline (**CIT**).^277^ The deimination of arginine impacts protein function, protein–protein interactions, and hydrogen-bonding ability,^458^ thus greatly affecting the protein phenotype. Native citrullination is catalyzed by protein arginine deiminases (PADs), which regulate many cellular processes.^459^ Aberrant PADs correlate with several autoimmune diseases and cancers.^103,458,459^

**Figure 46:**
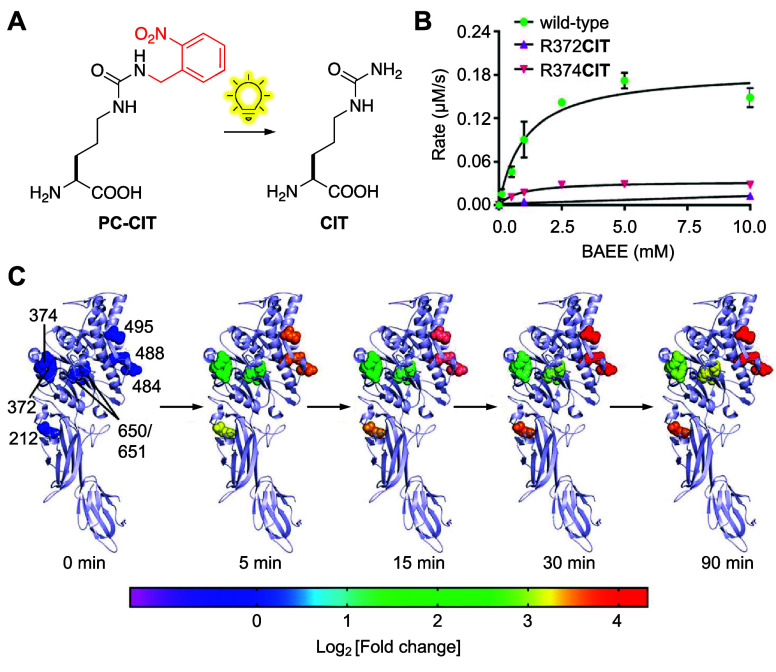
Site-specific citrullination versus autocitrullination on the effect of PAD4 activity. (A) Structure of *o-*nitrobenzyl citrulline, which is photolyzed to citrulline with UV light. (B) Michaelis–Menten kinetics of wild-type, R372**CIT**, and R374**CIT** mutants of PAD4.^103^ (C) Quantitative proteomics of PAD4 autocitrullination showed preferential citrullination at arginines 484, 488, and 495. Panels (B,C) are reproduced with permission from ref (103). Copyright 2021 The Author(s) under CC-BY 4.0 International license.

Prior to genetic code expansion techniques, experimental citrullination of proteins was achieved through treatment with PADs. While efficient, this approach inferred global, rather than site-specific citrullination.^103^ With intentions like those employed for encoding **PC-DAP** above and fluoro-tyrosines in section 4, a photocaging approach was used to mask this near-natural amino acid and to generate a hydrophobic handle for the synthetase. Thus, an *o*-nitrobenzyl caging group was installed to yield the photocaged citrulline (**PC–CIT**, Figure 46A).

Genetic encoding of **PC–CIT** was possible with an *Ec*LeuRS mutant (PC–CITRS, Table 1) that was identified through a screen of preexisting synthetase mutants.^103^ Following the robust, selective expression of EGFP-39**PC–CIT** in mammalian cells with PC–CITRS, **PC–CIT** was incorporated into PAD4 to investigate a previously debated effect of citrullination on PAD4 activity.^460,461^ As native citrullination by PAD4 is calcium dependent, the effect on activity due to individual citrullination events on PAD4, now possible with this genetic code expansion approach, could be studied in a calcium-free environment. Incorporation of **PC–CIT** at R372 and R374 in PAD4, followed by irradiation and decaging, represented the first example of site-specific citrullination. The aforementioned sites were selected for their role in autocitrullination (self-deimination) and for their location within the active site of PAD4. Site-specific citrullination of these active site residues resulted in a decrease in substrate conversion (Figure 46B), likely due to the conformational changes that occurred upon loss of the positive charge of arginine. This result contrasted the change in activity that is observed when PAD4 is citrullinated at multiple sites (generated through calcium treatment to initiate autocitrulliantion). Autocitrullinated PAD4 maintains activity levels similar to noncitrullinated PAD4, thus indicating that citrullination at R372 and R374 may not be the main driving forces of activity.^103^ An additional quantitative proteomics analysis of autocitrullinated PAD4 validated that R372 and R374 are indeed autocitrullinated, but other arginines (212/218, 484/488/495, and 650/651) undergo citrullination at a higher rate (Figure 46C).

Though not applied for the standard means of optical control, the photocaged analogues of **DAP** and **CIT** were used as incorporation handles to expand the genetic code with near-natural amino acids. These applications provided valuable insight into the mechanism of valinomycin synthetase oligomerization and cyclization^102^ and yielded the first study of site-specific versus global citrullination of the deiminase PAD4.^103^ With the ability to incorporate small, reactive ncAAs, the scope of genetic code expansion is greatly widened and will continue to prove a valuable tool for the investigation of many biological processes. Future works are also expected to take advantage of the temporal control provided with these photolabile motifs.

## 11 Conclusions and Future Perspectives

Optical control of protein function provides precise, temporal control of protein activation in response to a noninvasive light stimulus for investigation independent of other cellular factors. Interactions between proteins and their regulators and substrates are highly complex and can lead to a number of possible cellular responses, making the investigation of the exact roles played by individual proteins a historically difficult task. Moreover, protein functions and their interactions are highly dynamic and are often regulated in a temporal and spatial fashion. A single protein involved in cell signaling, for example, is a member of a highly complex network whose activity can have implications on the up- or down-regulation of dozens of other signaling molecules in numerous other pathways, and this activation and deactivation can depend on the state of the cell cycle and the localization of the protein within the cell. Light represents an ideal tool to dissect these mechanisms, as it can be readily controlled temporally and spatially. It triggers biological processes with unrivaled speed and provides tunability based on intensity and wavelength. The ability to selectively turn on the activity of a given protein without perturbing the cell in any other way allows for the precise investigation into the effects of the function of an individual protein on cellular behavior, providing insights into the natural and disease function of complex cellular mechanisms.

In this review, we discussed the genetic encoding and applications of the different photocaged amino acids used to date to achieve optical control of protein function, often through rational design. Caging groups are light-removable protecting groups that are strategically placed on biologically active molecules, such as proteins, to block their function until the caging group is removed through exposure to light. In addition, photoswitchable amino acids have been developed that provide reversible light-control of protein function. Photolabile caging groups and photoswitchable motifs have been used to optically regulate lysine, tyrosine, cysteine, serine, histidine, glutamate, aspartate, and phenylalanine derivatives. The evolution of orthogonal tRNA and aminoacyl tRNA synthetases to selectively encode these noncanonical amino acids has allowed for expansion of the genetic code in bacteria, mammalian cells, yeast, zebrafish, frogs, mice, and nematodes, and thus generation of light-activated proteins directly in these biological systems.

The introduction of caging groups onto the side chains of critical amino acid residues can introduce steric blocking and provide control over varying molecular interactions, including 1) key electrostatic interactions with neighboring residues within an active site, 2) hydrogen bonding with an enzyme substrate or cofactor, 3) metal ion coordination, 4) functions such as nucleophilicity, basicity, or acidity, or 5) protein folding. Because of the wide range of functionalities that can be controlled via caging groups, many proteins have been placed under optical control, including those involved in cell signaling pathways, regulators of gene expression, protein localization sequences, post-translational modification writers, nanobodies, and more (see Table 2).

The site-specific placement of photoresponsive amino acids provides distinct advantages compared to other methods of protein control. The genetically defined caging group placement afforded by genetic code expansion allows for rational design of light-triggered proteins, as key residues can be blocked and their function investigated. Further, the small size of the caging group relative to larger protein fusions used in classic optogenetic approaches yields minimal perturbation of protein folding, thus improving the translation of the observed effects to native systems. A related advantage is the generation of the wild-type protein with no scarring or sequence alterations upon photolytic cleavage of the caging group. An additional layer of photocaged amino acid tunability is available in the decaging wavelengths for each synthetic caging group. Some caging groups are most efficiently activated through irradiation with 365 nm light, which allows handling of the caged protein under ambient light with no specific precautions. Many photocaging groups can be cleaved with blue light (405 nm), which enables use of standard confocal imaging lasers for protein activation with exquisite spatial resolution. In general, the caging groups used in noncanonical amino acid mutagenesis are stable to excitation wavelengths used in the imaging of standard fluorescent reporters, such as EGFP, mCherry, and DsRed. Further, while beyond the scope of this review, the design principles for generating light-activated proteins can be directly utilized in the generation of small molecule-activated proteins, simply by utilizing amino acids that carry small molecule-cleavable groups in conjunction with their matching tRNA/tRNA synthetase machinery.^462−467^

These benefits render genetic code expansion complementary to other optogenetic approaches. However, limitations exist as well. While a wide range of caged amino acids have been genetically encoded, the evolution of selective aminoacyl tRNA synthetases for new amino acids may not always succeed. The resultant expression of ncAA-containing proteins may also require optimization, depending on sequence, caging group installation site, and organism. Further, while the photochemistry of optically activated amino acids is continuously being improved, the susceptibility of nitro groups to reduction in bacterial cells can limit their applicability in certain contexts.

While biological insights have been gained from genetic code expansion with photocaged amino acids, in particular in cell signaling, the field has not yet made the transition from methodology development to hypothesis-driven studies. As genetic code expansion materials become increasingly accessible, we foresee a plethora of novel applications expanding our collective knowledge of the dynamics and spatiotemporal intricacies of protein function. Additionally, we expect computational design of photocaged proteins and synthetases to play an increasing role in the future. With the broad range of ncAAs available for genetic code expansion, the introduction of optical control to any protein will provide the spatiotemporal control necessary for precise investigation of protein function and will inevitably lead to substantial discoveries in biological processes relevant to human physiology and disease.
